# Metal-Free Aminohalogenation of Quinones With Alkylamines and NXS at Room Temperature

**DOI:** 10.3389/fchem.2022.917371

**Published:** 2022-05-20

**Authors:** Jia Li, Yu-An Li, Ge Wu, Xu Zhang

**Affiliations:** ^1^ Department of Neurology, First Affiliated Hospital of Wenzhou Medical University, Wenzhou, China; ^2^ Department of Orthopaedics Surgery, The Second Affiliated Hospital and Yuying Children’s Hospital of Wenzhou Medical University, Wenzhou, China; ^3^ School of Pharmaceutical Sciences, Wenzhou Medical University, Wenzhou, China

**Keywords:** metal-free, aminohalogenation, quinones, NXS, radical reactions

## Abstract

A simple and practical strategy for intermolecular aminohalogenation of quinone with alkyl amines and NXS was developed, in which haloamines generated *in situ* were employed as bifunctional reagents. The reaction system is reliable, efficient and wide in substrate range, which is suitable for the two-fold aminochlorination of 1, 4-benzoquinones, large-scale reaction and late-stage modification of pharmaceuticals.

## Introduction

2-amino-3-halogenated naphthoquinones are of great academic and industrial value with widespread applications in medicinal chemistry areas (Calil et al., 2019). Some of these compounds possess low cytotoxicity and tumor growth ability in multiple myeloma (Prachayasittikul et al., 2014). They are used as multipotent agents for treating Alzheimer’s disease and potential new drug for treating leber’s hereditary optic neuropathy (Varricchio et al., 2020). Historically, the developed synthetic approaches relied on dihalogenation reaction/nucleophilic substitution step-wise strategy (Shvartsberg et al., 2009; Sieveking et al., 2014) and halogenation of 2-amino naphthoquinones (Vaidya et al., 2020). Despite the success of these reliable methods, the access of 2-amino-3-iodinated naphthoquinones is still an impalpable assignment. The associated issues related to substrate pre-functionalization, poor chemical selectivity (Shi et al., 1996) and step economy would limit their potential applications in the design and development of new naphthoquinone drugs. Therefore, the development of novel synthetic approach toward highly functionalized 2-amino-3-halogenated naphthoquinones derivatives based on direct aminohalogenation, would be a more promising and attractive protocol.

**SCHEME 1:**
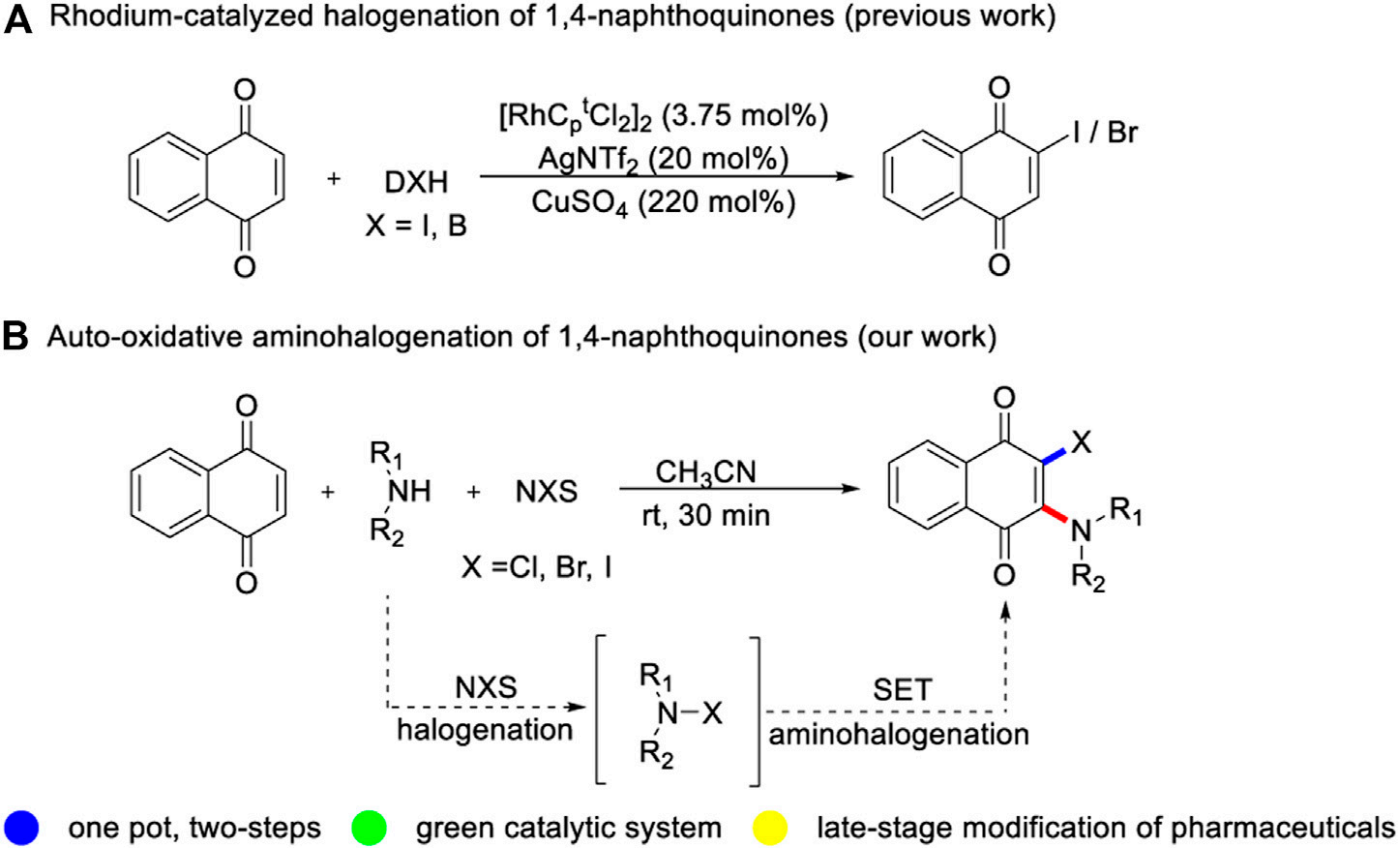
Motivation and aminohalogenation of quinones.

In the past decade, transition-metal catalyzed C-H functionalization of naphthoquinone with different cross-coupling partners has made incredible progress mainly exploiting its’ high electrophilic properties (Fujiwara et al., 2011; Lisboa et al., 2011; Ilangovan et al., 2013; Galloway et al., 2017; Yu et al., 2018; Liu et al., 2019; Dong et al., 2020; Zhu et al., 2020). In contrast, the cross-coupling of naphthoquinone with electrophiles is still challenging but significant, and only scattered cases have been reported (Zhang et al., 2015). Wang and Bi group described a copper-catalyzed radical trifluoromethylation reaction between naphthoquinone and Togni reagent (Wang et al., 2013; Fang et al., 2014). Bower and coworkers developed a rhodium-catalyzed bromination and iodination of naphthoquinone, using electrophilic halogenation reagent (Jardim et al., 2016a; Jardim et al., 2016b) (Scheme 1A). These studies indicate that cheap and readily available naphthoquinone has broad application prospects, and monofunctional naphthoquinone derivatives can be facilely synthesized by two-component reaction. Nevertheless, there is still a great demand for developing novel multi-component cascade reaction to strealmine the variety of 2,3-difunctionalized quinones without transition metal catalysis (Zeng et al., 2019). Herein, we disclose a metal-free auto-oxidative aminohalogenation of quinones with a series of alkyl amines and NXS (X = Cl, Br, I) at room temperature (Scheme 1B). This synthetic strategy provides a concise and efficient strategy for the preparation of structurally diverse 2-amino-3-halogenated quinones derivatives with the formation of C-N and C-X bonds. In addition, the practical utility of current transformation was applied in large-scale reaction, late-stage functionalization of atomoxetine and two-fold aminochlorination of benzoquinone.

## Results and Discussion

We began our studies using 1,4-naphthoquinone **1a**, morpholine **2a** and NCS **3a** as model substrates to test the feasibility of multi-component aminohalogenation reactions (Table 1). To our delight, when the model reaction was catalyzed by CuCl in toluene at 50 °C under N_2_ atmosphere for 30 min, the desired 2-chloro-3-morpholino naphthoquinone was isolated in 97% yield (entry 1). It is noteworthy that the transformation efficiency was not profound affected by the choice of different copper catalysts (entries 1–4). To our surprise, the target product 4a was still obtained in excellent yield, even if the reaction was performed in the absence of copper salt (entry 5). This result clearly indicates that the copper salts only worked as a promoter rather than a catalyst. Further solvent screening showed that the high efficiency and high yields relied on polarity of solvent, and the reaction was not effective in polar solvent (entries 6–9). Remarkably, the yield of **4a** is almost unaffected by lowering the reaction temperature to room temperature (entry 10). In addition, it is worth mentioning that this transformation is insensitive to reaction atmosphere, whether it is air or oxygen (entries 11–12).

**TABLE 1:** Reaction optimization.^a^.

Entry	Catalyst	Solvent	t (°C)	Yield (%)^b^
1	CuCl	toluene	50	97
2	CuI	toluene	50	92
3	Cu(OAc)_2_	toluene	50	91
4	CuBr	toluene	50	95
5		toluene	50	90
6		THF	50	72
7		DCE	50	80
8		CH_3_CN	50	94
9		DMSO	50	31
10		CH_3_CN	25	95
11^c^		CH_3_CN	25	90
12^d^		CH_3_CN	25	92

^a)^ Reaction conditions unless specified otherwise: **1a** (0.2 mmol), **2a** (0.6 mmol), **3a** (0.6 mmol) and catalyst (0.02 mmol) in solvent (2.0 ml) were stirred at 50°C under N_2_ for 30 min ^b)^ Isolated yield. ^c)^ Under an O_2_ atm ^d)^ Under an air atmosphere.

**SCHEME 2:**
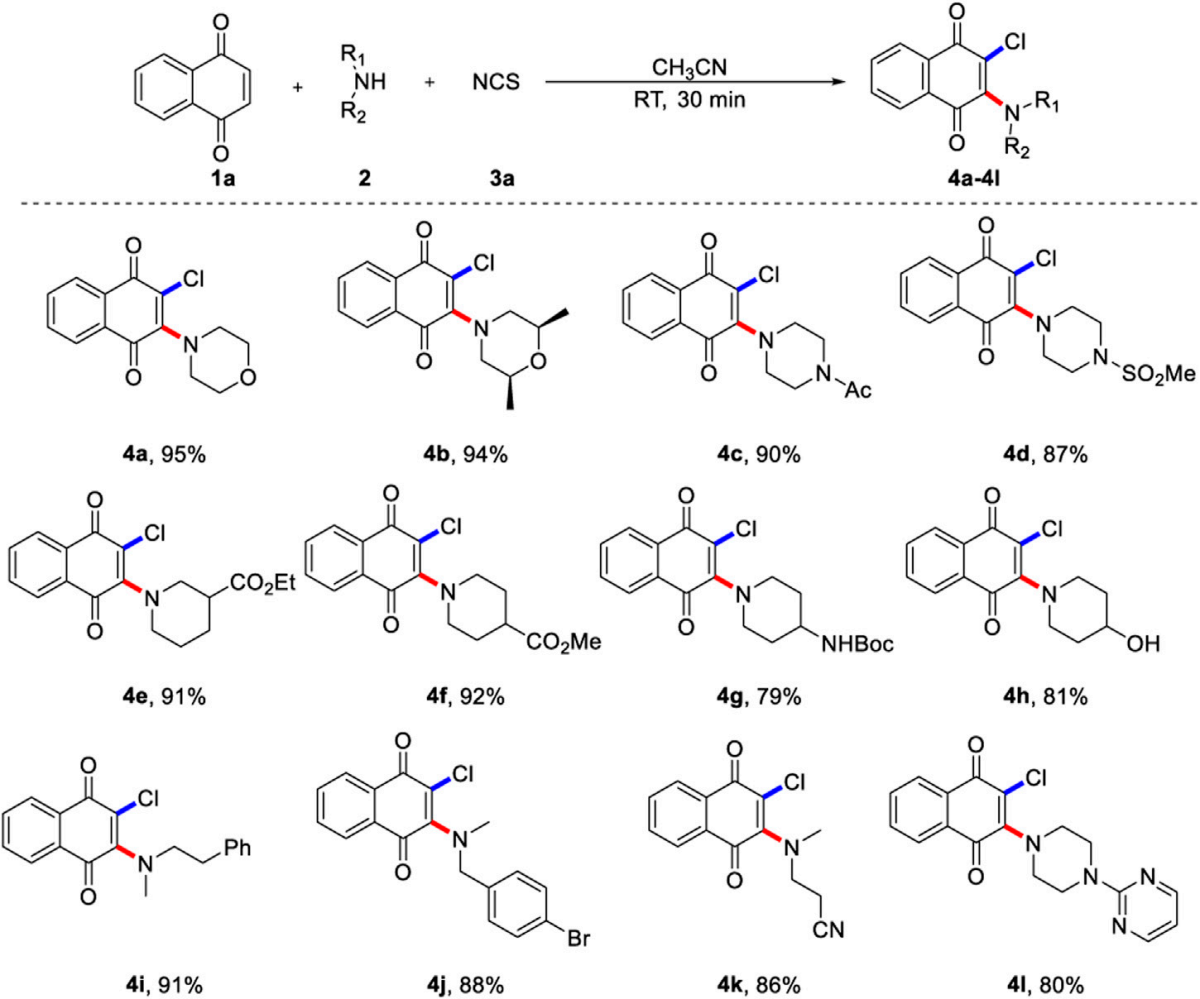
Scope of 1,4-naphthoquinone aminochlorinationa. a) Reaction conditions unless specified otherwise: 1a (0.2 mmol), 2 (0.6 mmol) and NCS (0.6 mmol) in CH3CN (2.0 ml) were stirred at room temperature under N2 for 30 min, isolated yields are given.

With the optimized reaction condition in hand, an array of alkylamines was examined for current three-component oxidative aminochlorination (Scheme 2). It was found that various cyclic secondary amines (**2a**-**2h**, **2l**) reacted smoothly, and the corresponding product (**4a**-**4h**, **4l**) were obtained with moderate to excellent yields. Many important synthetic functional groups, such as methyl, acyl, sulfonyl, ester, hydroxyl, cyano, and halogen, are well compatible under the standard reaction condition. It is worth noting that base-sensitive substrates **2c** and **2e**-**2g** are also competent coupling partners, and they are commonly fragile in strongly basic medium. Apart from cyclic organic amines, chain organic amines (**2i**-**2k**) are also suitable for vinylchlorination, showing diversity of substrate range. In addition, *N*-Methyl-4-bromobenzylamine (**2k**) is feasible and could be utilized for further transformation by classical cross-coupling reactions. Importantly, aromatic heterocycles such as pyrimidine was tolerated and afforded the desired product in 80% yield.

**SCHEME 3:**
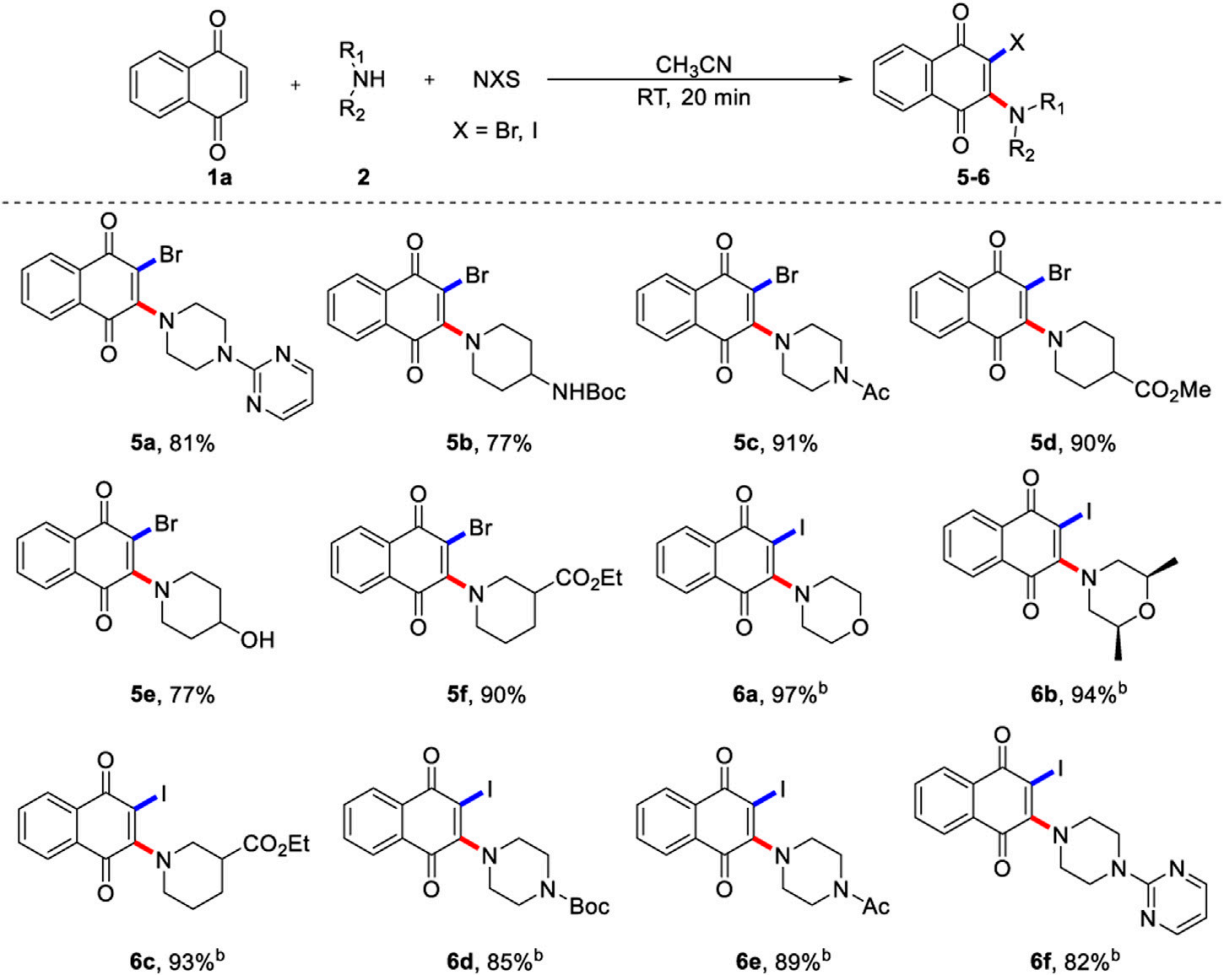
Scope of 1,4-naphthoquinone aminobromination and aminoiodinationa. a) Reaction conditions unless specified otherwise: 1a (0.2 mmol), 2 (0.6 mmol) and NBS (0.6 mmol) in CH3CN (2.0 ml) were stirred at room temperature under N2 for 20 min, isolated yields are given. b) Using 0.6 mmol NIS react for 2 min, isolated yields are given.

Encouraged by the versatility of the aminochlorination of 1,4-naphthoquinone, we turned our attention to evaluating the feasibility of aminobromination and aminoiodination (Scheme 3), because these compounds could not be accessed by conventional methods. In these cases, morpholine (**6a**, **6b**), piperidine (**5b**, **5d**-**5f**, **6c**) and piperazine (**5a**, **5c**, **6d**-**6f**) all showed good tolerance, and the corresponding products could be obtained in excellent yields. Interestingly, the aminobromination and aminoiodination of 1,4-naphthoquinone displayed a similar reaction trends to that of aminochlorination reaction. Surprisingly, the aminoiodination of 1,4-naphthoquinone can be completed quickly in a short reaction time, which only takes 2 min, and the TLC silica gel plate was very clean. It is possible that the reactivity of N-X is enhanced by increasing the radius of halogen atom.

Of course, current three-component aminohalogenation is particularly practical and useful because *N*-iodoamine is too unstable to be separated as an initial reactant. In addition, we also investigated the capacity of different alkenes, such as styrene, cinnamonitrile, 2-benzylidenemalononitrile and acrylic ester, and no aminohalogenation products were detected, which suggested that the autoxidation and unique reactivity of 1,4-naphthoquinone was the essential for this transformation.

**SCHEME 4:**
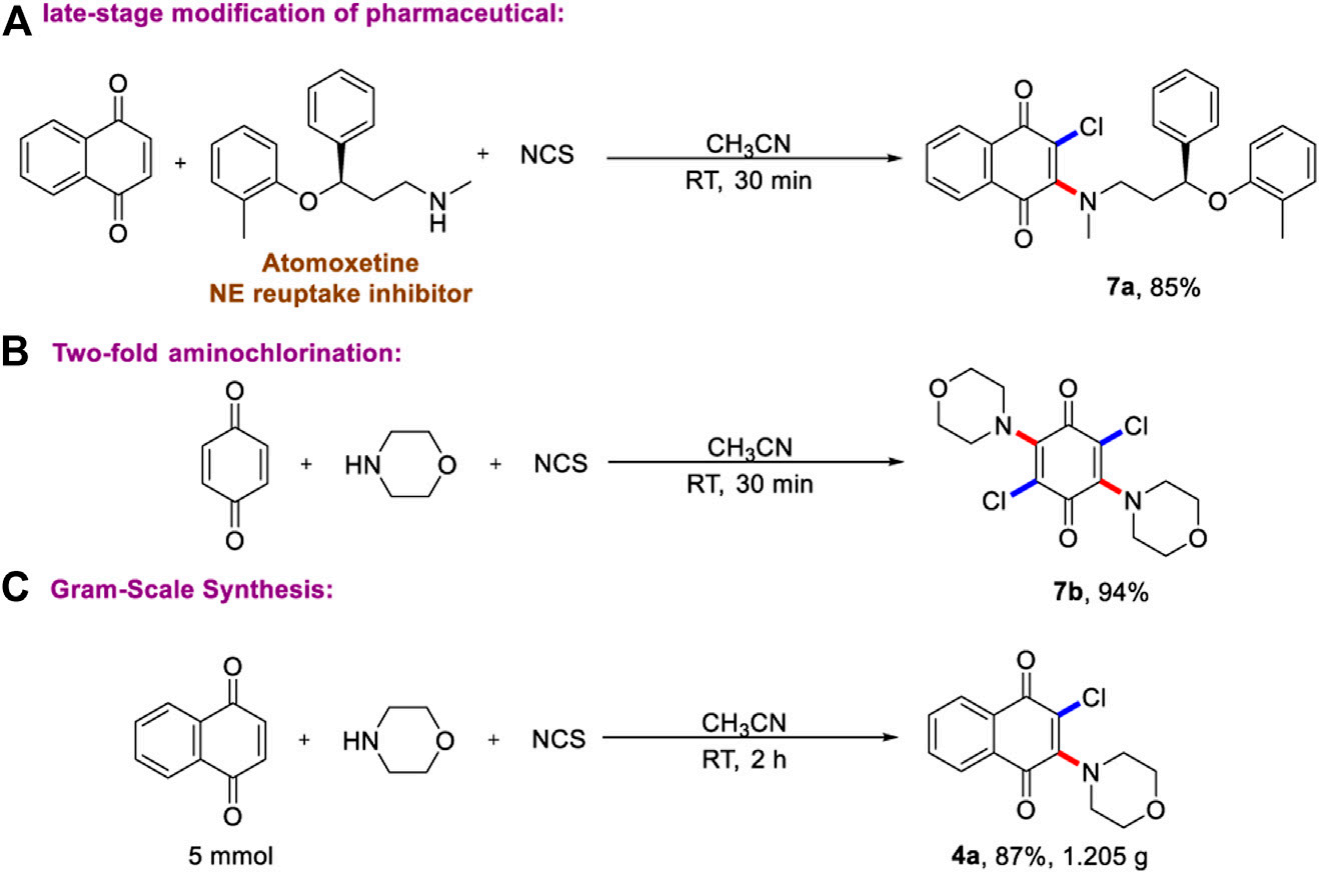
Synthetic application

Given the operational simplicity of this protocol and practical application, we next prepared the drug molecular analogs using late-stage modification of pharmaceuticals. As described in Scheme 4A, good chemo-selective vinylchlorination of atomoxetine (trade name: Strattera) on N-Me group has been achieved, and the corresponding product has been obtained in excellent yield. Most importantly, the two-fold aminochlorination of benzoquinone has been accomplished (Scheme 4B), and the molecular symmetric product was produced in 94% yield. This result highlights the power of the current multi-component aminohalogenation reaction, which could not be realized by any known synthetic methods. Finally, as shown in Scheme 4C, the gram-scale reaction proves the reliability and repeatability of two-step one-pot aminohalogenation reaction.

**SCHEME 5:**
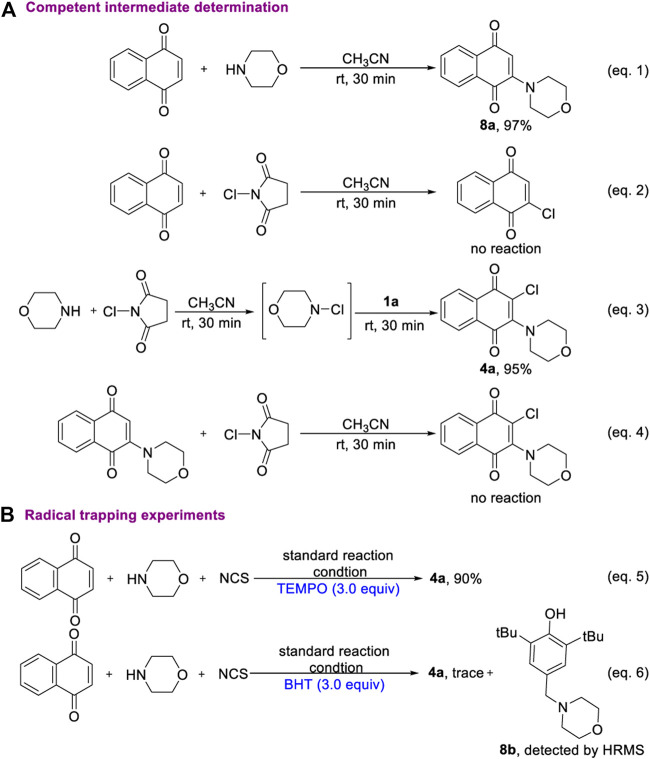
Mechanism consideration and control experiments

To shed light on the mechanism of oxidative aminohalogenation of 1,4-naphthoquinone, some consideration and control experiments were designed and performed (Scheme 5). First, the reaction of 1,4-naphthoquinone with morpholine produces a completely converted oxidative amination product **8a** (eq. 1). In contrast, there was no reaction between 1,4-naphthoquinone and NCS (eq. 2). This result suggests that the amination of 1,4-naphthoquinone initiated a mutli-component reaction owing to the nucleophilicity of nitrogen. Next, we aim to elucidate the chlorination of the intermediate **8a**. Two blank experiments were conducted, in which the mixture of morpholine and NCS was stirred in CH_3_CN for 30 min, and then 1,4-naphthoquinone was added to furnish the corresponding product with excellent yield (eq. 3). This result clearly shows that three-component aminohalogenation reaction is initiated by the formation of N-X bonds. However, the electrophilic chlorination reaction of enaminone **8a** did not occur (eq. 4). Based on the experimental result, the progress of an initial oxidative amination followed by elctrophilic capture of the resulting enaminone **8a** with electrophilic chlorination reagent could be excluded. A possible intermolecular radical cascade mechanism includes hemolytic cleavage of the nitrogen-halogen bonds and addition to electron-deficient 1,4-naphthoquinone. Interestingly, the aminochlorination of 1,4-naphthoquinone was hardly affected when 3.0 equivalent TEMPO was added (eq. 5). In addition, the BHT-captured product was detected by HRMS, which indicated that *N*-radical species might be generated during the transformation process of current reaction (eq. 6).

**SCHEME 6:**
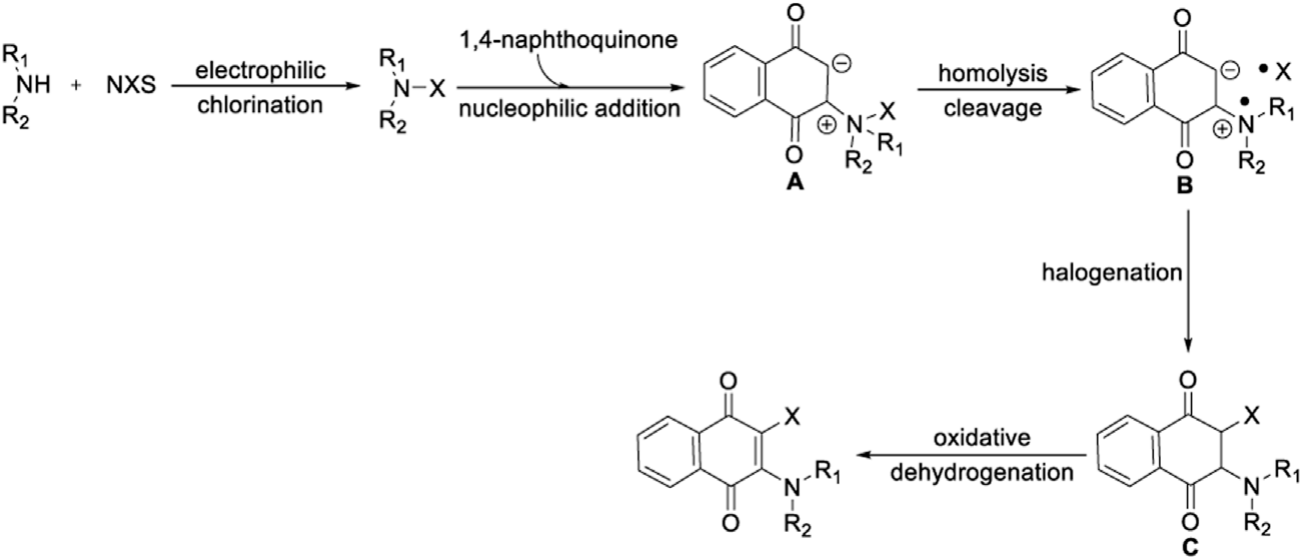
Proposed reaction mechanism

According to these experimental results and related literature, a reasonable reaction mechanism of 1,4-naphthoquinone oxidative aminohalogenation is proposed in Scheme 6. The initial electrophilic halogenation of alkylamine with NXS generated the N-X reagents (Ruffoni et al., 2019). Then, the resulting N-X and electron-deficient 1,4-naphthoquinone undergo nucleophilic addition (Yang et al., 2018) to produce intermediate **A**, and followed by homolytically cleaving to generate aminium and halide radical **B** (Hendrick and Wang, 2015), Finally, the key substance **C** is formed by rapid halogenation, and the corresponding products are obtained by oxidative dehydrogenation (Deng et al., 2015).

## Conclusion

In summary, we have developed a straightforward and feasible method of oxidative aminohalogenation of quinones with alkyamines and NXS. The prominent feature of multi-component reactions is that *N*-haloamines are used as bifunctional reagents, the reaction conditions are mild and simple, the reaction time is short, and the functional group are excellent tolerated. Furthermore, the two-fold aminochlorination and late-stage functionalization of pharmaceuticals demonstrated the highly synthetic value and great potential in the field of medicinal chemistry and discovery of novel drugs.

## Data Availability

The original contributions presented in the study are included in the article/Supplementary Material, further inquiries can be directed to the corresponding author.
