# Supplementary material for: Metal-Free Aminohalogenation of Quinones With Alkylamines and NXS at Room Temperature
Source: Front Chem. 2022 May 20;10:917371. doi: 10.3389/fchem.2022.917371 (PMC9189915; doi:10.3389/fchem.2022.917371)
Supplement: Supplementary file 1 [file DataSheet1.docx]

Supplementary Material

**Metal-Free Aminohalogenation of Quinones with Alkylamines and NXS at Room Temperature**

Jia Li^1^ Yu-An Li^3^ Ge Wu^2^*

^1^Department of Neurology, First Affiliated Hospital of Wenzhou Medical University, Wenzhou 325000, People’s Republic of China

^2^School of Pharmaceutical Sciences, Wenzhou Medical University, Wenzhou 325035, People’s Republic of China

^3^Department of Orthopaedics Surgery, The Second Affiliated Hospital and Yuying Children’s Hospital of Wenzhou Medical University, Wenzhou 325000, People’s Republic of China

***Correspondence:** Wu Ge wuge@wmu.edu.cn

**Table of Contents**

**(1) General considerations, experimental data………….……S2-S18**

**(2) ^1^H, ^13^C and ^19^F NMR spectra of products……………..…S19-S54**

**General Procedure of Metal-Free Aminochlorination of Quinones with Alkylamines and NCS：**

Pay attention to mixing procedure:

A 25 mL Schlenk tube equipped with a stir bar was charged with 1,4-naphthoquinone (0.2 mmol), NCS (0.6 mmol) and 2 mL CH_3_CN, then, the addition of alkylamines (0.6 mmol) using a pipette, the tube was fitted with a rubber septum, and then it was evacuated and refilled with N_2_ three times, then the septum was replaced by a Teflon screwcap under oxygen flow. The reaction mixture was stirred at room temperature for 30 min. The reaction mixture was diluted with 10 mL of ethyl ether, filtered through a pad of silica gel, followed by washing the pad of the silica gel with the same solvent (20 mL), concentrated under reduced pressure. The residue was then purified by flash chromatography on silica gel to provide the corresponding product.

**General Procedure of Metal-Free Aminobromination of Quinones with Alkylamines and NBS：**

Pay attention to mixing procedure:

A 25 mL Schlenk tube equipped with a stir bar was charged with 1,4-naphthoquinone (0.2 mmol), NBS (0.6 mmol) and 2 mL CH_3_CN, then, the addition of alkylamines (0.6 mmol) using a pipette, the tube was fitted with a rubber septum, and then it was evacuated and refilled with N_2_ three times, then the septum was replaced by a Teflon screwcap under oxygen flow. The reaction mixture was stirred at room temperature for 30 min. The reaction mixture was diluted with 10 mL of ethyl ether, filtered through a pad of silica gel, followed by washing the pad of the silica gel with the same solvent (20 mL), concentrated under reduced pressure. The residue was then purified by flash chromatography on silica gel to provide the corresponding product.

**General Procedure of Metal-Free Aminoiodination of Quinones with Alkylamines and NIS：**

Pay attention to mixing procedure:

A 25 mL Schlenk tube equipped with a stir bar was charged with 1,4-naphthoquinone (0.2 mmol), NIS (0.6 mmol) and 2 mL CH_3_CN, then, the addition of alkylamines (0.6 mmol) using a pipette, the tube was fitted with a rubber septum, and then it was evacuated and refilled with N_2_ three times, then the septum was replaced by a Teflon screwcap under oxygen flow. The reaction mixture was stirred at room temperature for 2 min. the reaction mixture was diluted with 10 mL of ethyl ether, filtered through a pad of silica gel, followed by washing the pad of the silica gel with the same solvent (20 mL), concentrated under reduced pressure. The residue was then purified by flash chromatography on silica gel to provide the corresponding product.

**Mechanism investigation:**

A 25 mL Schlenk tube equipped with a stir bar was charged with 1,4-naphthoquinone (0.2 mmol), morpholine (0.6 mmol) dissolved in CH_3_CN (2.0mL). The reaction mixture was stirred at room temperature for 30 min. The reaction mixture was diluted with 10 mL of ethyl ether, filtered through a pad of silica gel, followed by washing the pad of the silica gel with the same solvent (20 mL), concentrated under reduced pressure. The residue was then purified by flash chromatography on silica gel to provide the corresponding product 8a (isolated 97% yield).

A 25 mL Schlenk tube equipped with a stir bar was charged with 1,4-naphthoquinone (0.2 mmol), NCS (0.6 mmol) dissolved in CH_3_CN (2.0mL). The reaction mixture was stirred at room temperature for 30 min. The reaction was filtered through a pad of Celite and diluted with ethyl acetate (10 mL), no chlorination product was detected by GC-MS.

A 25 mL Schlenk tube equipped with a stir bar was charged with morpholine (0.6 mmol), NCS (0.6 mmol) dissolved in CH_3_CN (2.0mL), this mixture was stirred for 30 min at room temperature. Then the addition of 1,4-naphthoquinone (0.2 mmol), then it was stirred for 30 min. The residue was then purified by flash chromatography on silica gel to provide the corresponding product 4a (isolated 95% yield).

A 25 mL Schlenk tube equipped with a stir bar was charged with 8a (0.2 mmol), NCS (0.6 mmol) dissolved in CH_3_CN (2.0mL), this mixture was stirred for 30 min at room temperature. The reaction was filtered through a pad of Celite and diluted with ethyl acetate (10 mL), no chlorination product was detected by GC-MS.

A 25 mL Schlenk tube equipped with a stir bar was charged with 1,4-naphthoquinone (0.2 mmol), NCS (0.6 mmol), TEMPO (0.6 mmol) and 2 mL CH_3_CN, then, the addition of morpholine (0.6 mmol) using a pipette, the tube was fitted with a rubber septum, and then it was evacuated and refilled with N_2_ three times, then the septum was replaced by a Teflon screwcap under oxygen flow. The reaction mixture was stirred at room temperature for 30 min. The reaction mixture was diluted with 10 mL of ethyl ether, filtered through a pad of silica gel, followed by washing the pad of the silica gel with the same solvent (20 mL), concentrated under reduced pressure. The residue was then purified by flash chromatography on silica gel to provide the corresponding product.

A 25 mL Schlenk tube equipped with a stir bar was charged with 1,4-naphthoquinone (0.2 mmol), NCS (0.6 mmol), BHT (0.6 mmol) and 2 mL CH_3_CN, then, the addition of morpholine (0.6 mmol) using a pipette, the tube was fitted with a rubber septum, and then it was evacuated and refilled with N_2_ three times, then the septum was replaced by a Teflon screwcap under oxygen flow. The reaction mixture was stirred at room temperature for 30 min. The reaction mixture was diluted with 10 mL of ethyl ether, filtered through a pad of silica gel, BHT-trapped product was detected by GC-MS, which suggest the amine free-radical was generated under the current reaction condition.


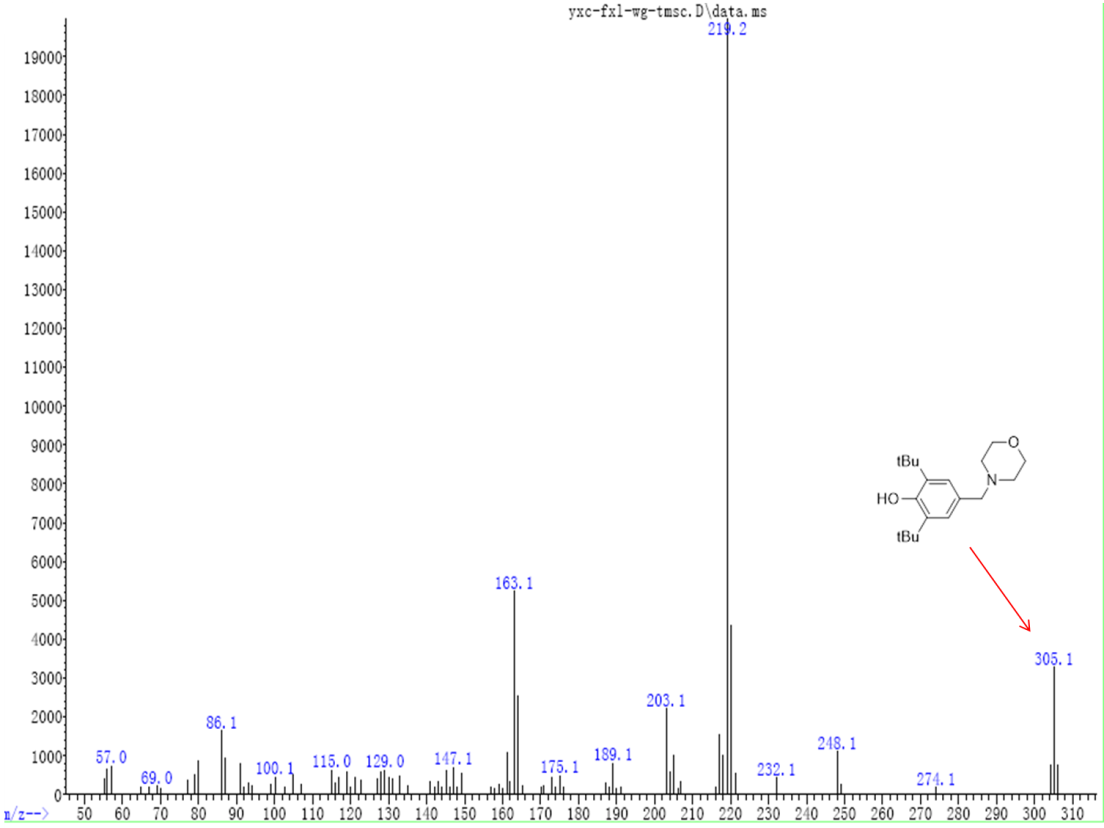


**Characterization of Products in Details:**

**2-chloro-3-morpholinonaphthalene-1,4-dione**

Following the general procedure, using (petroleum ether : EtOAc = 9 : 1) as the eluant afforded a dark violet solid (52.6 mg, 95% yield), Mp = 85-86℃. **^1^H NMR** (500 MHz, CDCl_3_): δ 8.12 (dd, *J* = 7.3, 1.7 Hz, 1H), 8.01 (dd, *J* = 7.3, 1.7 Hz, 1H), 7.70 (pd, *J* = 7.4, 1.6 Hz, 2H), 3.88 (t, *J* = 4.5 Hz, 4H), 3.64 (t, *J* = 4.5 Hz, 4H); **^13^C NMR** (125MHz, CDCl_3_): δ 181.7, 178.0, 149.6, 134.2, 133.2, 131.5, 131.3, 126.9, 126.6, 123.2, 67.6, 51.8; **HRMS** (ESI): calcd for C_14_H_13_NO_3_Cl [M + H]^+^ 278.0584, found 278.0580.

**2-chloro-3-((2S,6R)-2,6-dimethylmorpholino)naphthalene-1,4-dione**

Following the general procedure, using (petroleum ether : EtOAc = 9 : 1) as the eluant afforded a dark violet solid (57.3 mg, 94% yield), Mp = 127-128℃. **^1^H NMR** (500 MHz, CDCl_3_): δ 8.16 (dd, *J* = 7.1, 1.8 Hz, 1H), 8.05 (dd, *J* = 7.1, 1.8 Hz, 1H), 7.75 (pd, *J* = 7.4, 1.6 Hz, 2H), 3.92 (dqd, *J* = 12.4, 6.2, 1.9 Hz, 2H), 3.71 (d, J = 13.2 Hz, 2H), 3.11 (dd, *J* = 13.1, 10.2 Hz, 2H), 1.26 (d, *J* = 6.3 Hz, 6H); **^13^C NMR** (125MHz, CDCl_3_): δ 181.9, 178.1, 149.5, 134.2, 133.2, 131.7, 131.4, 126.9, 126.7, 122.9, 72.5, 56.8, 18.7; **HRMS** (ESI): calcd for C_16_H_17_NO_3_Cl [M + H]^+^ 306.0897, found 306.0894.

2-(4-acetylpiperazin-1-yl)-3-chloronaphthalene-1,4-dione

Following the general procedure, using (petroleum ether : EtOAc = 9 : 1) as the eluant afforded a dark violet liquid (57.2 mg, 90% yield). **^1^H NMR** (500 MHz, CDCl_3_): δ 8.17 (dd, *J* = 7.1, 1.8 Hz, 1H), 8.07 (dd, *J* = 7.1, 1.8 Hz, 1H), 7.75 (pd, *J* = 7.4, 1.6 Hz, 2H), 3.82-3.61 (m, 8H), 2.20 (s, 3H); **^13^C NMR** (125MHz, CDCl_3_): δ 181.8, 178.1, 169.3, 149.8, 134.3, 133.4, 131.5, 131.4, 127.0, 126.7, 124.7, 51.20, 47.1, 42.3, 21.4; **HRMS** (ESI): calcd for C_16_H_15_N_2_O_3_NaCl [M + Na]^+^ 341.0669, found 341.0668.

2-chloro-3-(4-(methylsulfonyl)piperazin-1-yl)naphthalene-1,4-dione

Following the general procedure, using (petroleum ether : EtOAc = 9 : 1) as the eluant afforded a dark violet solid (61.6 mg, 87% yield), Mp = 84-85℃. **^1^H NMR** (500 MHz, CDCl_3_): δ 8.15 (d, *J* = 7.0 Hz, 1H), 8.05 (d, *J* = 8.5 Hz, 1H), 7.74 (pd, *J* = 7.4, 1.6 Hz, 2H), 3.70 (t, *J* = 4.8 Hz, 4H), 3.44 (t, *J* = 4.8 Hz, 4H), 2.87 (s, 3H); **^13^C NMR** (125MHz, CDCl_3_): δ 181.8, 178.1, 149.7, 134.4, 133.6, 131.5, 131.3, 127.1, 126.8, 125.5, 50.8, 46.5, 34.8; **HRMS** (ESI): calcd for C_15_H_15_N_2_O_4_NaSCl [M + Na]^+^ 377.0339, found 377.0336.

ethyl 1-(3-chloro-1,4-dioxo-1,4-dihydronaphthalen-2-yl)piperidine-3-carboxylate

Following the general procedure, using (petroleum ether : EtOAc = 9 : 1) as the eluant afforded a dark violet liquid (63.1 mg, 91% yield). **^1^H NMR** (500 MHz, CDCl_3_): δ 8.14 (dd, *J* = 7.3, 1.7 Hz, 1H), 8.04 (dd, *J* = 7.1, 1.9 Hz, 1H), 7.71 (pd, *J* = 7.4, 1.6 Hz, 2H), 4.18 (q, *J* = 7.1 Hz, 2H), 3.97-3.93 (m, 1H), 3.70-3.65 (m, 1H), 3.48 (dd, *J* = 13.2, 9.8 Hz, 1H), 3.38 (ddd, *J* = 13.4, 10.0, 3.2 Hz, 1H), 2.85 (tt, *J* = 9.9, 3.9 Hz, 1H), 2.18 (dt, *J* = 8.7, 4.7 Hz, 1H), 1.96-1.91 (m, 1H), 1.80 (tdd, *J* = 10.6, 6.5, 3.0 Hz, 2H), 1.27 (t, *J* = 7.1 Hz, 3H); **^13^C NMR** (125MHz, CDCl_3_): δ 181.8, 178.2, 173.2, 150.6, 134.1, 133.2, 131.6, 131.5, 126.9, 126.6, 123.9, 60.7, 53.4, 52.0, 42.3, 26.9, 25.3, 14.2; **HRMS** (ESI): calcd for C_18_H_18_NO_4_NaCl [M + Na]^+^ 370.0822, found 370.0815.

methyl 1-(3-chloro-1,4-dioxo-1,4-dihydronaphthalen-2-yl)piperidine-4-carboxylate

Following the general procedure, using (petroleum ether : EtOAc = 9 : 1) as the eluant afforded a dark violet liquid (61.3 mg, 92% yield). **^1^H NMR** (500 MHz, CDCl_3_): δ 8.14 (dd, *J* = 7.3, 1.7 Hz, 1H), 8.04 (dd, *J* = 7.1, 1.9 Hz, 1H), 7.72 (pd, *J* = 7.4, 1.6 Hz, 2H), 3.87-3.81 (m, 2H), 3.76 (s, 1H), 3.38 (ddd, *J* = 13.7, 10.9, 3.1 Hz, 1H), 2.65 (tt, *J* = 10.7, 4.2 Hz, 0H), 2.12-1.96 (m, 4H); **^13^C NMR** (125MHz, CDCl_3_): δ 181.9, 178.1, 174.8, 150.5, 134.1, 133.1, 131.6, 131.5, 126.9, 126.6, 123.6, 51.9, 51.1, 40.6, 28.9; **HRMS** (ESI): calcd for C_17_H_16_NO_4_NaCl [M + Na]^+^ 356.0666, found 356.0666.

tert-butyl (1-(3-chloro-1,4-dioxo-1,4-dihydronaphthalen-2-yl)piperidin-4-yl)carbamate

Following the general procedure, using (petroleum ether : EtOAc = 9 : 1) as the eluant afforded a dark violet solid (61.6 mg, 79% yield), Mp = 109-110℃. **^1^H NMR** (500 MHz, CDCl_3_): δ 8.14 (dd, *J* = 7.3, 1.7 Hz, 1H), 8.04 (dd, *J* = 7.1, 1.9 Hz, 1H), 7.71 (pd, *J* = 7.4, 1.6 Hz, 2H), 4.59 (brs, 1H), 3.85-3.77 (m, 3H), 3.40 (ddd, *J* = 13.8, 11.3, 2.6 Hz, 2H), 2.11 (dd, *J* = 12.8, 3.7 Hz, 2H), 1.72-1.62 (m, 2H), 1.49 (s, 9H); **^13^C NMR** (125MHz, CDCl_3_): δ 181.9, 178.1, 155.1, 150.5, 134.1, 133.1, 131.6, 131.5, 126.9, 126.6, 123.6, 79.5, 67.9, 60.4, 50.6, 47.4, 33.4, 28.4; **HRMS** (ESI): calcd for C_20_H_23_N_2_O_4_NaCl [M + Na]^+^ 413.1244, found 413.1247.

2-chloro-3-(4-hydroxypiperidin-1-yl)naphthalene-1,4-dione

Following the general procedure, using (petroleum ether : EtOAc = 9 : 1) as the eluant afforded a dark violet solid (47.1 mg, 81% yield), Mp = 88-89℃. **^1^H NMR** (500 MHz, CDCl_3_): δ 8.14 (dd, *J* = 7.3, 1.7 Hz, 1H), 8.04 (dd, *J* = 7.1, 1.9 Hz, 1H), 7.71 (pd, *J* = 7.4, 1.6 Hz, 2H), 4.03 (tt, *J* = 8.3, 3.9 Hz, 1H), 3.84 (dt, *J* = 13.6, 4.5 Hz, 2H), 3.43 (ddd, *J* = 13.0, 9.2, 3.2 Hz, 2H), 2.11 (ddt, *J* = 13.6, 7.1, 4.0 Hz, 2H), 1.81 (dtd, *J* = 13.2, 9.0, 4.0 Hz, 3H); **^13^C NMR** (125MHz, CDCl_3_): δ 182.0, 178.2, 150.6, 134.1, 133.2, 131.7, 131.5, 126.9, 126.6, 123.3, 67.2, 49.1, 35.3; **HRMS** (ESI): calcd for C_15_H_14_NO_3_NaCl [M + Na]^+^ 314.0560, found 314.0561.

2-chloro-3-(methyl(phenethyl)amino)naphthalene-1,4-dione

Following the general procedure, using (petroleum ether : EtOAc = 9 : 1) as the eluant afforded a dark violet liquid (59.1 mg, 91% yield). **^1^H NMR** (500 MHz, CDCl_3_): δ 8.12 (dd, *J* = 7.2, 1.7 Hz, 1H), 8.01 (dd, *J* = 7.4, 1.7 Hz, 1H), 7.70 (dtd, *J* = 16.3, 7.4, 1.6 Hz, 2H), 7.29-7.23 (m, 4H), 7.19-7.14 (m, 1H), 3.85 (t, *J* = 7.5 Hz, 2H), 3.29 (s, 3H), 3.01 (t, *J* = 7.5 Hz, 2H); **^13^C NMR** (125MHz, CDCl_3_): δ 182.2, 178.0, 151.3, 138.6, 134.0, 133.0, 131.6, 128.9, 128.6, 126.9, 126.6, 126.4, 122.3, 57.1, 42.5, 35.1; **HRMS** (ESI): calcd for C_19_H_16_NO_2_NaCl [M + Na]^+^ 348.0767, found 348.0766.

2-((4-bromobenzyl)(methyl)amino)-3-chloronaphthalene-1,4-dione

Following the general procedure, using (petroleum ether : EtOAc = 9 : 1) as the eluant afforded a dark violet liquid (68.4 mg, 88% yield). **^1^H NMR** (500 MHz, CDCl_3_): δ 8.16 (dd, *J* = 7.3, 1.7 Hz, 1H), 8.06 (dd, *J* = 7.1, 1.9 Hz, 1H), 7.73 (pd, *J* = 7.4, 1.6 Hz, 2H), 7.51 (d, *J* = 8.4 Hz, 2H), 7.25 (d, *J* = 8.3 Hz, 2H), 4.70 (s, 2H), 3.10 (s, 3H); **^13^C NMR** (125MHz, CDCl_3_): δ 182.1, 178.2, 151.3, 136.6, 134.1, 133.3, 131.8, 131.6, 130.9, 129.5, 127.0, 126.6, 124.4, 131.5, 58.6, 41.9; **HRMS** (ESI): calcd for C_18_H_13_NO_2_NaClBr [M + Na]^+^ 411.9716, found 411.9723.

3-((3-chloro-1,4-dioxo-1,4-dihydronaphthalen-2-yl)(methyl)amino)propanenitrile

Following the general procedure, using (petroleum ether : EtOAc = 9 : 1) as the eluant afforded a dark violet solid (47.1 mg, 86% yield), Mp = 119-120℃. **^1^H NMR** (500 MHz, CDCl_3_): δ 8.15 (dd, *J* = 7.3, 1.7 Hz, 1H), 8.07 (dd, *J* = 7.1, 1.9 Hz, 1H), 7.75 (pd, *J* = 7.4, 1.6 Hz, 2H), 3.83 (t, *J* = 6.9 Hz, 2H), 3.24 (s, 3H), 2.84 (t, *J* = 6.9 Hz, 2H); **^13^C NMR** (125MHz, CDCl_3_): δ 182.0, 178.3, 150.5, 134.4, 133.6, 131.4, 131.4, 127.1, 126.8, 117.9, 50.8, 41.7, 17.5; **HRMS** (ESI): calcd for C_14_H_11_N_2_O_2_NaCl [M + Na]^+^ 297.0407, found 297.0407.

2-chloro-3-(4-(pyrimidin-2-yl)piperazin-1-yl)naphthalene-1,4-dione

Following the general procedure, using (petroleum ether : EtOAc = 9 : 1) as the eluant afforded a dark violet liquid (56.6 mg, 80% yield). **^1^H NMR** (500 MHz, CDCl_3_): δ 8.41 (d, *J* = 4.8 Hz, 2H), 8.18 (dd, *J* = 7.3, 1.7 Hz, 1H), 8.08 (dd, *J* = 7.1, 1.9 Hz, 1H), 7.75 (pd, *J* = 7.4, 1.6 Hz, 2H), 6.62 (t, *J* = 4.8 Hz, 1H), 4.11 (t, *J* = 4.7 Hz, 4H), 3.72 (t, *J* = 4.7 Hz, 4H); **^13^C NMR** (125MHz, CDCl_3_): δ 182.0, 178.1, 161.5, 157.9, 150.2, 134.2, 133.3, 131.7, 131.5, 127.0, 126.7, 123.8, 110.4, 51.3, 44.7; **HRMS** (ESI): calcd for C_18_H_16_N_4_O_2_Cl [M + H]^+^ 355.0962, found 355.0953.

2-bromo-3-(4-(pyrimidin-2-yl)piperazin-1-yl)naphthalene-1,4-dione

Following the general procedure, using (petroleum ether : EtOAc = 9 : 1) as the eluant afforded a dark violet solid (64.5 mg, 81% yield), Mp = 131-132℃. **^1^H NMR** (500 MHz, CDCl_3_): δ 8.36 (d, *J* = 4.8 Hz, 2H), 8.13 (d, *J* = 7.0 Hz, 1H), 8.04 (d, *J* = 8.5 Hz, 1H), 7.73-7.69 (m, 2H), 6.56 (t, *J* = 4.8 Hz, 1H), 4.05 (q, *J* = 4.7 Hz, 4H), 3.70-3.67 (m, 4H); **^13^C NMR** (125MHz, CDCl_3_): δ 181.6, 178.2, 161.6, 157.9, 152.9, 134.2, 133.2, 131.5, 131.4, 127.1, 127.0, 117.9, 110.4, 51.7, 44.7; **HRMS** (ESI): calcd for C_18_H_16_N_4_O_2_Br [M + H]^+^ 399.0457, found 399.0453.

tert-butyl (1-(3-bromo-1,4-dioxo-1,4-dihydronaphthalen-2-yl)piperidin-4-yl)carbamate

Following the general procedure, using (petroleum ether : EtOAc = 9 : 1) as the eluant afforded a dark violet solid (66.8 mg, 77% yield), Mp = 113-114℃. **^1^H NMR** (500 MHz, CDCl_3_): δ 8.14 (dd, *J* = 7.3, 1.7 Hz, 1H), 8.03 (dd, *J* = 7.1, 1.9 Hz, 1H), 7.71 (pd, *J* = 7.4, 1.6 Hz, 2H), 4.58 (brs, 1H), 3.84-3.78 (m, 3H), 3.39 (ddd, *J* = 13.8, 11.3, 2.6 Hz, 2H), 2.11 (dd, *J* = 13.5, 3.4 Hz, 2H), 1.71-1.63 (m, 2H), 1.49 (s, 9H); **^13^C NMR** (125MHz, CDCl_3_): δ 182.0, 178.1, 155.2, 150.5, 134.1, 133.2, 131.7, 131.5, 126.9, 126.8, 126.7, 50.6, 48.3, 33.5, 32.3, 28.5; **HRMS** (ESI): calcd for C_20_H_23_N_2_O_4_NaBr [M + Na]^+^ 457.0739, found 457.0732.

2-(4-acetylpiperazin-1-yl)-3-bromonaphthalene-1,4-dione

Following the general procedure, using (petroleum ether : EtOAc = 9 : 1) as the eluant afforded a dark violet solid (65.9 mg, 91% yield), Mp = 70-71℃. **^1^H NMR** (500 MHz, CDCl_3_): δ 8.17 (dd, *J* = 7.3, 1.7 Hz, 1H), 8.07 (dd, *J* = 7.1, 1.9 Hz, 1H), 7.74 (pd, *J* = 7.4, 1.6 Hz, 2H), 3.84-3.61 (m, 8H), 2.20 (s, 3H); **^13^C NMR** (125MHz, CDCl_3_): δ 181.6, 178.3, 169.3, 152.7, 134.3, 133.5, 131.3, 131.2, 127.1, 120.0, 119.4, 51.6, 42.3, 21.4; **HRMS** (ESI): calcd for C_16_H_15_N_2_O_3_NaBr [M + Na]^+^ 385.0164, found 385.0155.

methyl 1-(3-bromo-1,4-dioxo-1,4-dihydronaphthalen-2-yl)piperidine-4-carboxylate

Following the general procedure, using (petroleum ether : EtOAc = 9 : 1) as the eluant afforded a dark violet liquid (67.9 mg, 90% yield). **^1^H NMR** (500 MHz, CDCl_3_): δ 8.14 (dd, *J* = 7.3, 1.7 Hz, 1H), 8.02 (dd, *J* = 7.1, 1.9 Hz, 1H), 7.70 (pd, *J* = 7.4, 1.6 Hz, 2H), 3.83 (dd, *J* = 13.3, 4.1 Hz, 2H), 3.75 (s, 3H), 3.39 (ddd, *J* = 13.5, 10.5, 3.2 Hz, 2H), 2.64 (tt, *J* = 10.0, 4.4 Hz, 1H), 2.05 (pd, *J* = 10.4, 9.8, 3.6 Hz, 4H); **^13^C NMR** (125MHz, CDCl_3_): δ 181.7, 178.3, 174.9, 153.3, 134.1, 133.2, 131.5, 131.4, 127.0, 126.9, 117.8, 51.9, 51.5, 40.6, 28.9; **HRMS** (ESI): calcd for C_17_H_16_NO_4_NaBr [M + Na]^+^ 400.0160, found 400.0154.

2-bromo-3-(4-hydroxypiperidin-1-yl)naphthalene-1,4-dione

Following the general procedure, using (petroleum ether : EtOAc = 9 : 1) as the eluant afforded a dark violet solid (51.6 mg, 77% yield), Mp = 115-116℃. **^1^H NMR** (400 MHz, CDCl_3_): δ 8.11 (dd, *J* = 6.8, 2.1 Hz, 1H), 8.01 (dd, *J* = 6.7, 2.1 Hz, 1H), 7.69 (tt, *J* = 7.4, 5.7 Hz, 2H), 4.03 (tt, *J* = 8.3, 3.9 Hz, 1H), 3.83 (dt, *J* = 13.6, 4.7 Hz, 2H), 3.44 (ddd, *J* = 13.0, 9.2, 3.2 Hz, 2H), 2.14-2.06 (m, 3H), 1.83 (dtd, *J* = 12.7, 8.9, 3.7 Hz, 2H); **^13^C NMR** (100 MHz, CDCl_3_): δ 181.7, 178.3, 153.4, 134.1, 133.1, 131.5, 131.4, 127.0, 126.9, 117.1, 67.1, 49.5, 35.2; **HRMS** (ESI): calcd for C_15_H_14_NO_3_NaBr [M + Na]^+^ 358.0055, found 358.0061.

ethyl 1-(3-bromo-1,4-dioxo-1,4-dihydronaphthalen-2-yl)piperidine-3-carboxylate

Following the general procedure, using (petroleum ether : EtOAc = 9 : 1) as the eluant afforded a dark violet liquid (70.4 mg, 90% yield). **^1^H NMR** (400 MHz, CDCl_3_): δ 8.15-8.13 (m, 1H), 8.05-8.02 (m, 1H), 7.74-7.67 (m, 2H), 4.17 (q, *J* = 7.1 Hz, 2H), 3.97-3.92 (m, 1H), 3.68-3.63 (m, 1H), 3.47 (dd, *J* = 13.2, 9.8 Hz, 1H), 3.39 (ddd, *J* = 13.4, 10.2, 3.3 Hz, 1H), 2.86 (ddd, *J* = 10.0, 5.9, 3.9 Hz, 1H), 2.18-2.16 (m, 1H), 1.95-1.92 (m, 1H), 1.81 (ddd, *J* = 11.0, 6.4, 2.8 Hz, 2H), 1.26 (t, *J* = 7.1 Hz, 3H); **^13^C NMR** (100 MHz, CDCl_3_): δ 181.6, 178.3, 173.2, 153.4, 134.1, 133.2, 131.5, 131.4, 127.1, 126.9, 118.3, 60.7, 53.8, 52.5, 42.3, 26.9, 25.3, 14.3; **HRMS** (ESI): calcd for C_18_H_18_NO_4_NaBr [M + Na]^+^ 414.0317, found 414.0311.

2-iodo-3-morpholinonaphthalene-1,4-dione

Following the general procedure, using (petroleum ether : EtOAc = 9 : 1) as the eluant afforded a dark violet solid (71.6 mg, 97% yield), Mp = 90-91℃. **^1^H NMR** (400 MHz, CDCl_3_): δ 8.12 (dd, *J* = 7.3, 1.7 Hz, 1H), 8.02 (dd, *J* = 7.3, 1.7 Hz, 1H), 7.69 (pd, *J* = 7.4, 1.6 Hz, 2H), 3.91 (t, *J* = 4.5 Hz, 4H), 3.65 (t, *J* = 4.5 Hz, 4H); **^13^C NMR** (100 MHz, CDCl_3_): δ 180.8, 179.7, 157.9, 134.0, 133.3, 131.4, 130.1, 127.5, 127.1, 102.5, 67.6, 52.7; **HRMS** (ESI): calcd for C_14_H_13_NO_3_I [M + H]^+^ 369.9940, found 369.9941.

2-((2S,6R)-2,6-dimethylmorpholino)-3-iodonaphthalene-1,4-dione

Following the general procedure, using (petroleum ether : EtOAc = 9 : 1) as the eluant afforded a dark violet solid (74.6 mg, 94% yield), Mp = 126-127℃. **^1^H NMR** (400 MHz, CDCl_3_): δ 8.12 (dd, *J* = 7.3, 1.7 Hz, 1H), 8.02 (dd, *J* = 7.3, 1.7 Hz, 1H), 7.69 (pd, *J* = 7.4, 1.6 Hz, 2H), 3.96 (ddt, *J* = 12.6, 6.3, 3.2 Hz, 2H), 3.65 (d, *J* = 12.9 Hz, 2H), 3.16 (dd, *J* = 13.0, 10.2 Hz, 2H), 1.24 (d, *J* = 6.3 Hz, 6H); **^13^C NMR** (100 MHz, CDCl_3_): δ 180.9, 179.7, 157.5, 134.0, 133.2, 131.4, 130.1, 127.5, 127.1, 101.4, 72.4, 57.7, 18.6; **HRMS** (ESI): calcd for C_16_H_16_NO_3_NaI [M + Na]^+^ 420.0073, found 420.0079.

ethyl 1-(3-iodo-1,4-dioxo-1,4-dihydronaphthalen-2-yl)piperidine-3-carboxylate

Following the general procedure, using (petroleum ether : EtOAc = 9 : 1) as the eluant afforded a dark violet liquid (81.6 mg, 93% yield). **^1^H NMR** (400 MHz, CDCl_3_): δ 8.08 (dd, *J* = 7.3, 1.7 Hz, 1H), 8.00 (dd, *J* = 7.3, 1.7 Hz, 1H), 7.67 (pd, *J* = 7.4, 1.6 Hz, 2H), 4.15 (q, *J* = 7.1 Hz, 2H), 3.92-3.88 (m, 1H), 3.59 (dt, *J* = 13.4, 4.0 Hz, 1H), 3.51-3.37 (m, 2H), 2.88 (ddt, *J* = 14.0, 9.3, 4.0 Hz, 1H), 2.14 (dd, *J* = 9.3, 5.0 Hz, 1H), 1.94 (dq, *J* = 11.0, 6.7, 5.1 Hz, 1H), 1.80 (t, *J* = 9.3 Hz, 2H), 1.25 (t, *J* = 7.1 Hz, 3H); **^13^C NMR** (100 MHz, CDCl_3_): δ 180.7, 179.7, 173.2, 158.9, 133.9, 133.2, 131.5, 130.1, 127.4, 127.1, 103.3, 60.7, 54.3, 53.2, 42.2, 26.8, 25.2, 14.3; **HRMS** (ESI): calcd for C_18_H_18_NO_4_NaI [M + Na]^+^ 462.0178, found 462.0180.

tert-butyl 4-(3-iodo-1,4-dioxo-1,4-dihydronaphthalen-2-yl)piperazine-1-carboxylate

Following the general procedure, using (petroleum ether : EtOAc = 9 : 1) as the eluant afforded a dark violet liquid (79.5 mg, 85% yield). **^1^H NMR** (400 MHz, CDCl_3_): δ 8.10 (dd, *J* = 7.3, 1.7 Hz, 1H), 8.01 (dd, *J* = 7.3, 1.7 Hz, 1H), 7.68 (pd, *J* = 7.4, 1.6 Hz, 2H), 3.66 (t, *J* = 4.7 Hz, 4H), 3.56 (t, *J* = 4.7 Hz, 4H), 1.51 (s, 9H); **^13^C NMR** (100 MHz, CDCl_3_): δ 180.7, 179.7, 158.3, 154.8, 134.0, 133.3, 131.4, 130.0, 127.4, 127.2, 103.8, 80.2, 52.1, 28.5, 26.9; **HRMS** (ESI): calcd for C_19_H_21_N_2_O_4_NaI [M + Na]^+^ 491.0444, found 491.0443.

2-(4-acetylpiperazin-1-yl)-3-iodonaphthalene-1,4-dione

Following the general procedure, using (petroleum ether : EtOAc = 9 : 1) as the eluant afforded a dark violet liquid (73.0 mg, 89% yield). **^1^H NMR** (400 MHz, CDCl_3_): δ 8.08 (dd, *J* = 7.3, 1.7 Hz, 1H), 8.00 (dd, *J* = 7.3, 1.7 Hz, 1H), 7.68 (pd, *J* = 7.4, 1.6 Hz, 2H), 3.83 (dd, *J* = 6.5, 3.7 Hz, 2H), 3.69 (dd, *J* = 6.5, 3.4 Hz, 2H), 3.59 (dd, *J* = 6.3, 3.4 Hz, 2H), 3.54 (dd, *J* = 6.1, 3.8 Hz, 2H), 2.16 (s, 3H); **^13^C NMR** (100 MHz, CDCl_3_): δ 180.6, 179.6, 169.2, 158.2, 134.1, 133.5, 131.4, 130.0, 127.5, 127.2, 105.1, 52.1, 52.0, 47.1, 42.2, 21.5; **HRMS** (ESI): calcd for C_16_H_15_N_2_O_3_NaI [M + Na]^+^ 433.0025, found 433.0031.

2-iodo-3-(4-(pyrimidin-2-yl)piperazin-1-yl)naphthalene-1,4-dione

Following the general procedure, using (petroleum ether : EtOAc = 9 : 1) as the eluant afforded a dark violet liquid (73.1 mg, 82% yield). **^1^H NMR** (400 MHz, CDCl_3_): δ 8.38 (d, *J* = 4.8 Hz, 2H), 8.14 (dd, *J* = 7.3, 1.7 Hz, 1H), 8.06 (dd, *J* = 7.3, 1.7 Hz, 1H), 7.70 (pd, *J* = 7.4, 1.6 Hz, 2H), 6.58 (t, *J* = 4.8 Hz, 1H), 4.10 (t, *J* = 4.7 Hz, 4H), 3.70 (t, *J* = 4.7 Hz, 4H); **^13^C NMR** (100 MHz, CDCl_3_): δ 180.8, 179.7, 161.4, 158.4, 157.8, 134.0, 133.3, 131.5, 130.1, 127.5, 127.2, 110.3, 103.1, 52.2, 44.7; **HRMS** (ESI): calcd for C_18_H_16_N_4_O_2_I [M + H]^+^ 447.0318, found 447.0324.

(S)-2-chloro-3-(methyl(3-phenyl-3-(o-tolyloxy)propyl)amino)naphthalene-1,4-dione

Following the general procedure, using (petroleum ether : EtOAc = 9 : 1) as the eluant afforded a dark violet liquid (75.6 mg, 85% yield). **^1^H NMR** (500 MHz, CDCl_3_): δ7.99 (dd, *J* = 7.3, 1.7 Hz, 1H), 7.92 (dd, *J* = 7.3, 1.7 Hz, 1H), 7.64 (pd, *J* = 7.4, 1.6 Hz, 2H), 7.38-7.25 (m, 5H), 6.76-6.71 (m, 2H), 6.46 (d, *J* = 7.7 Hz, 2H), 5.24 (dd, *J* = 9.7, 3.8 Hz, 1H), 4.15 (ddd, *J* = 15.0, 9.3, 6.3 Hz, 1H), 3.63 (ddd, *J* = 14.2, 6.4, 3.7 Hz, 1H), 3.27 (s, 3H), 2.31-2.21 (m, 5H); **^13^C NMR** (125MHz, CDCl_3_): δ 181.9, 178.0, 155.5, 151.5, 141.7, 133.7, 132.8, 131.5, 130.4, 128.9, 128.8, 127.7, 126.7, 126.5,126.4, 126.2, 125.6, 123.2, 120.3, 112.0, 76.2, 52.0, 42.1, 37.4, 16.4; **HRMS** (ESI): calcd for C_27_H_24_NO_3_NaCl [M + Na]^+^ 468.1342, found 468.1347.

2,5-dichloro-3,6-dimorpholinocyclohexa-2,5-diene-1,4-dione

Following the general procedure, using (petroleum ether : EtOAc = 9 : 1) as the eluant afforded a dark violet solid (65.0 mg, 94% yield), Mp = 110-111℃. **^1^H NMR** (500 MHz, CDCl_3_): δ 3.86 (t, *J* = 4.5 Hz, 8H), 3.63 (t, *J* = 4.5 Hz, 8H); **^13^C NMR** (125MHz, CDCl_3_): δ 176.0, 148.1, 116.4, 67.6, 52.2; **HRMS** (ESI): calcd for C_14_H_16_N_2_O_4_NaCl_2_ [M + Na]^+^ 369.0385, found 369.0380.

**^1^H and ^13^C NMR spectra of products**

**
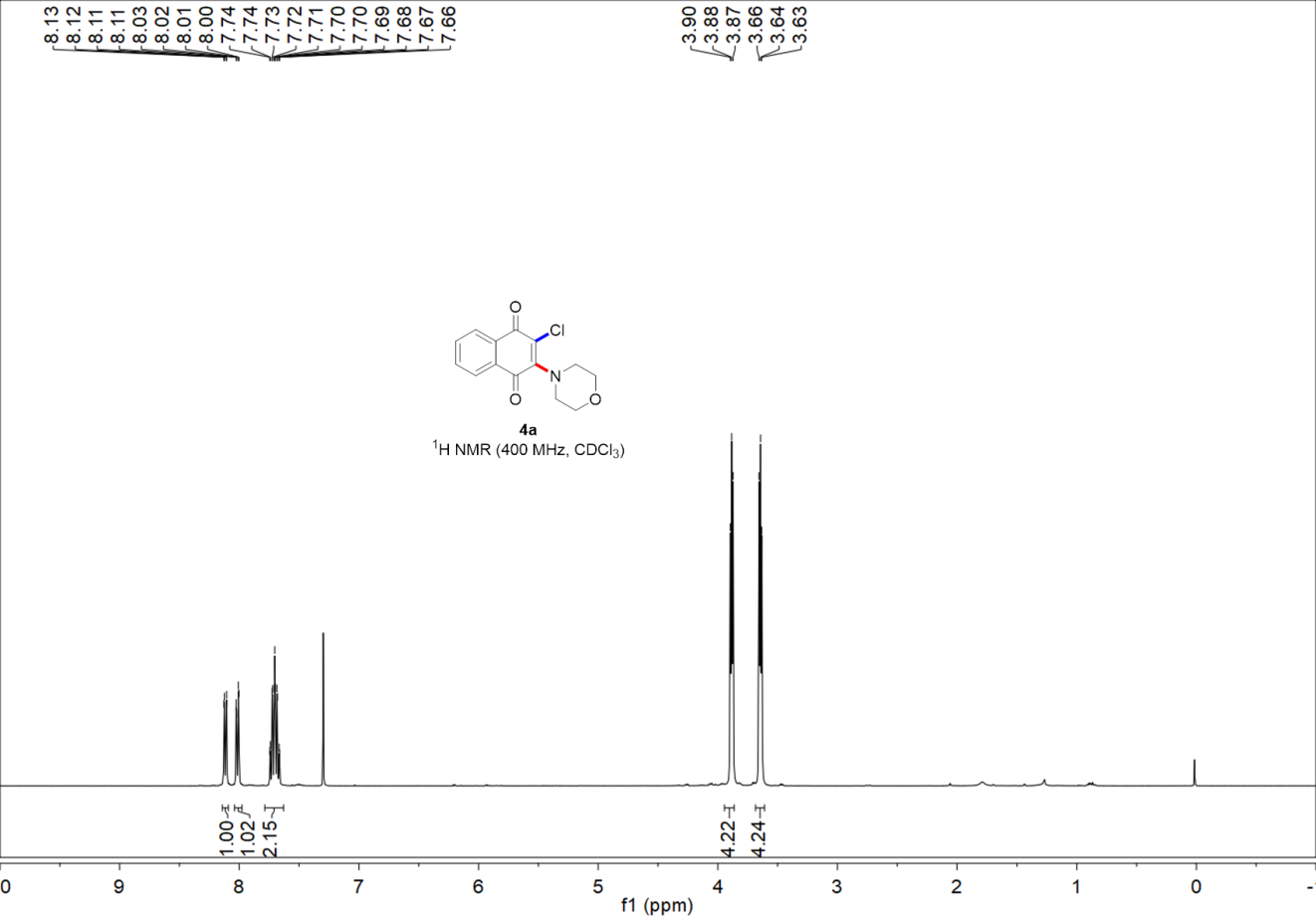
**

**
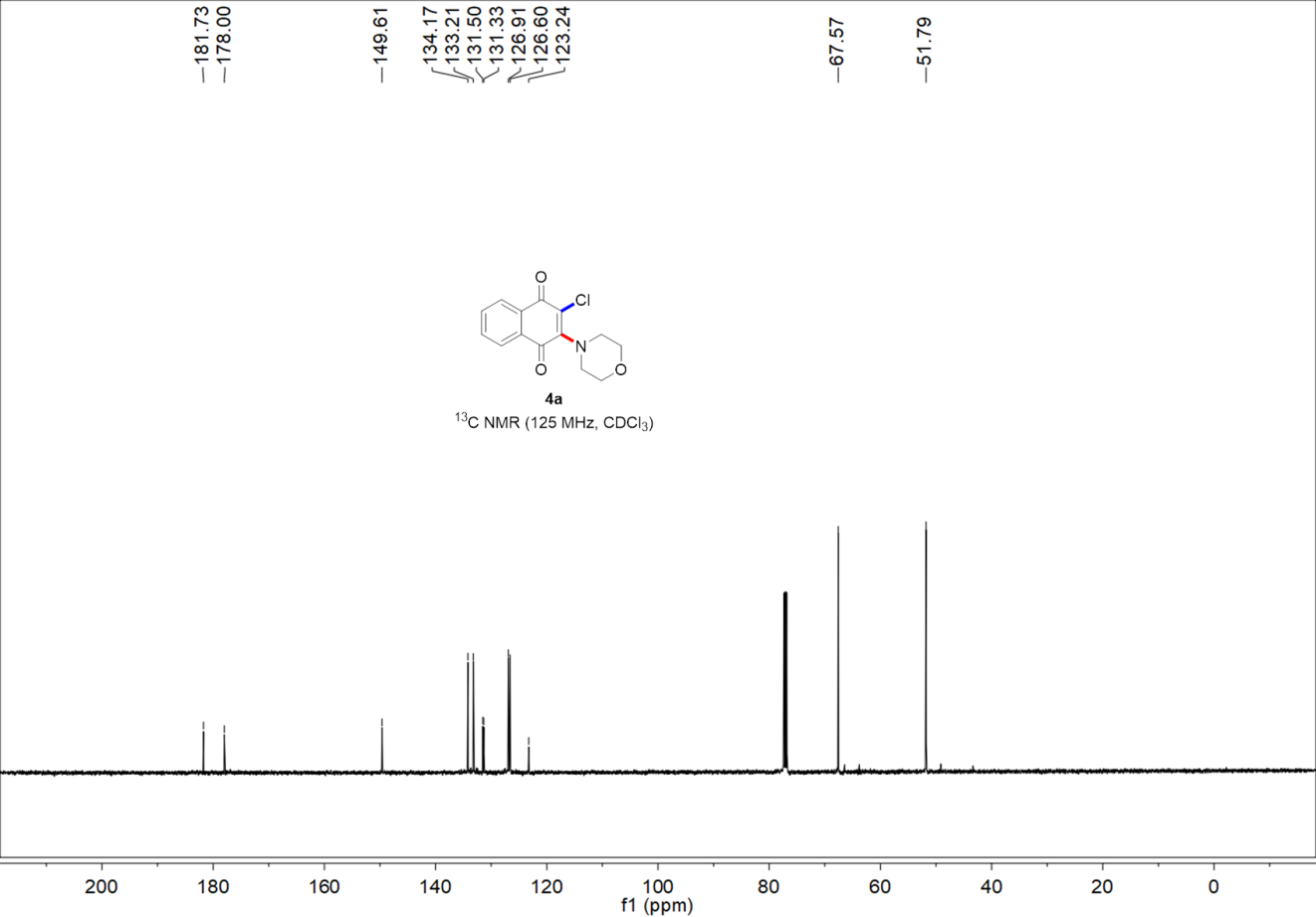
**

**
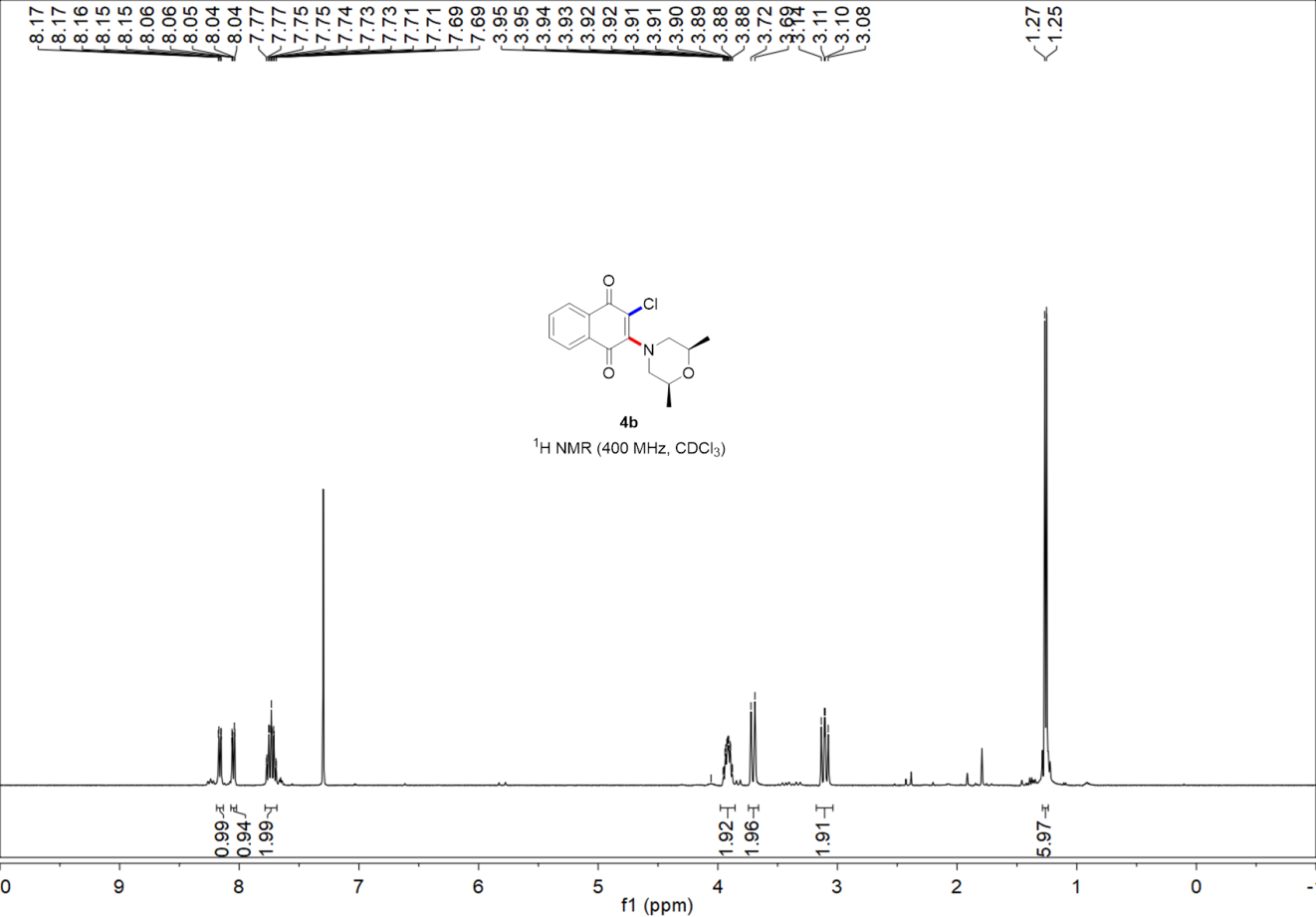
**

**
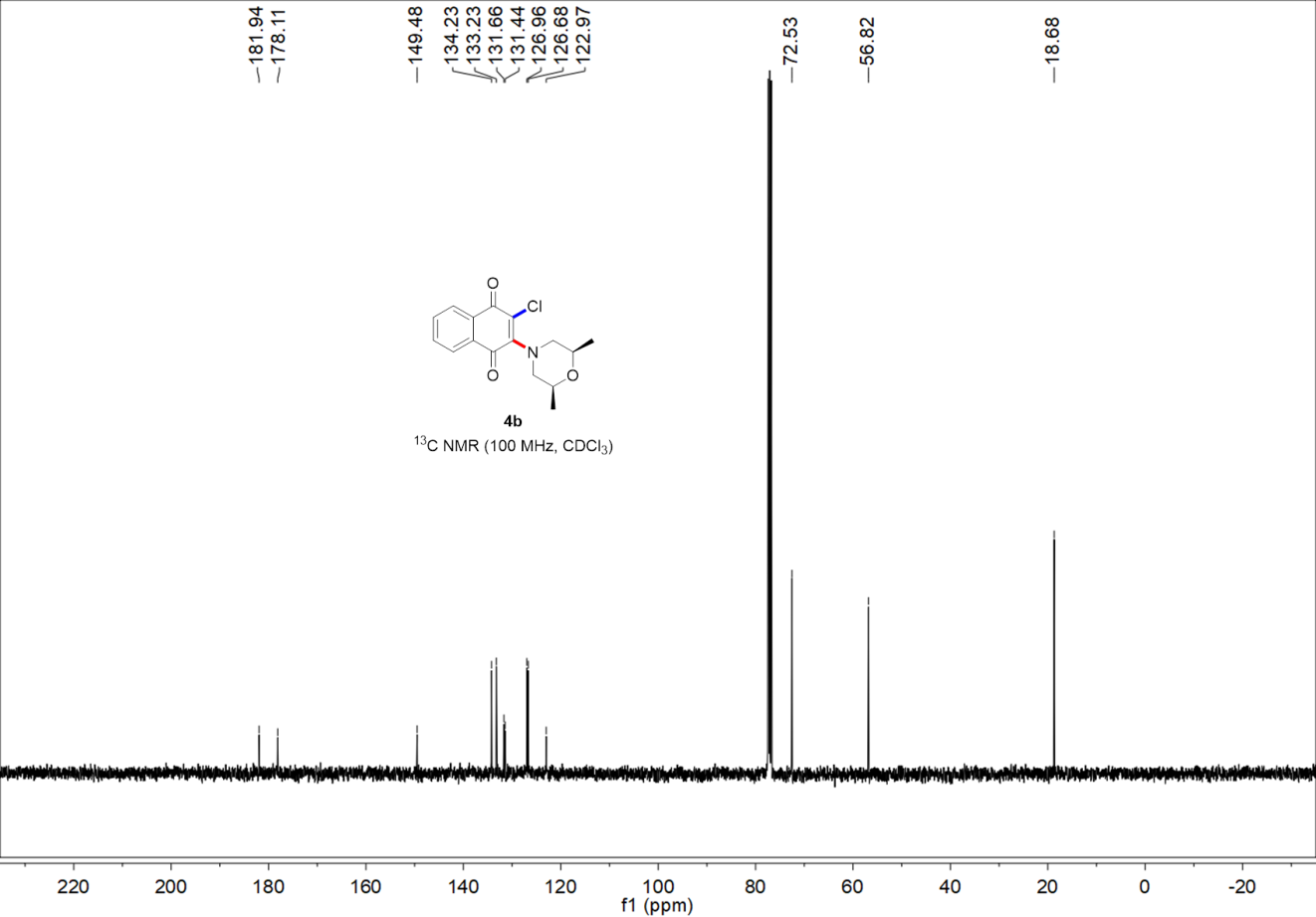
**

**
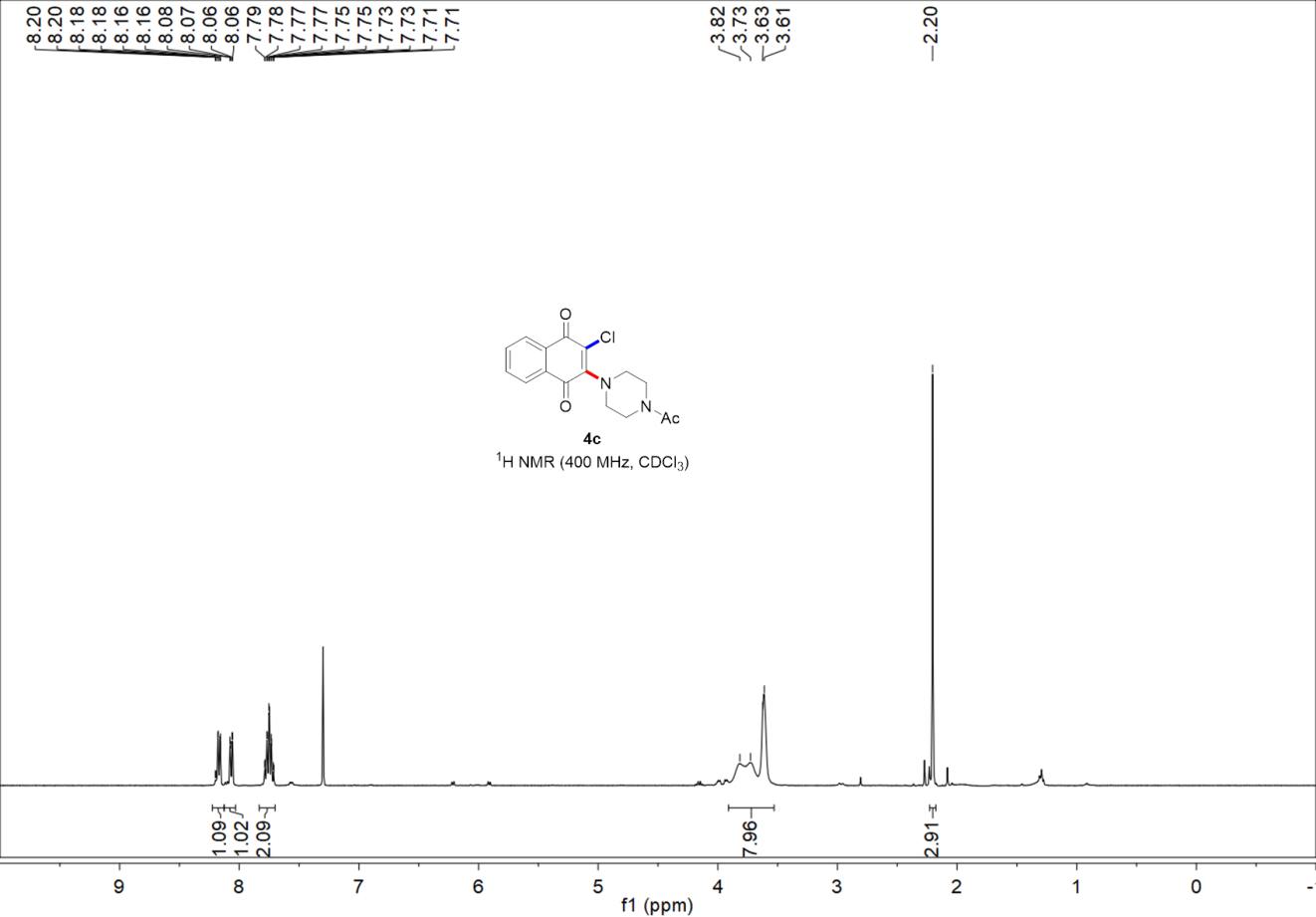
**

**
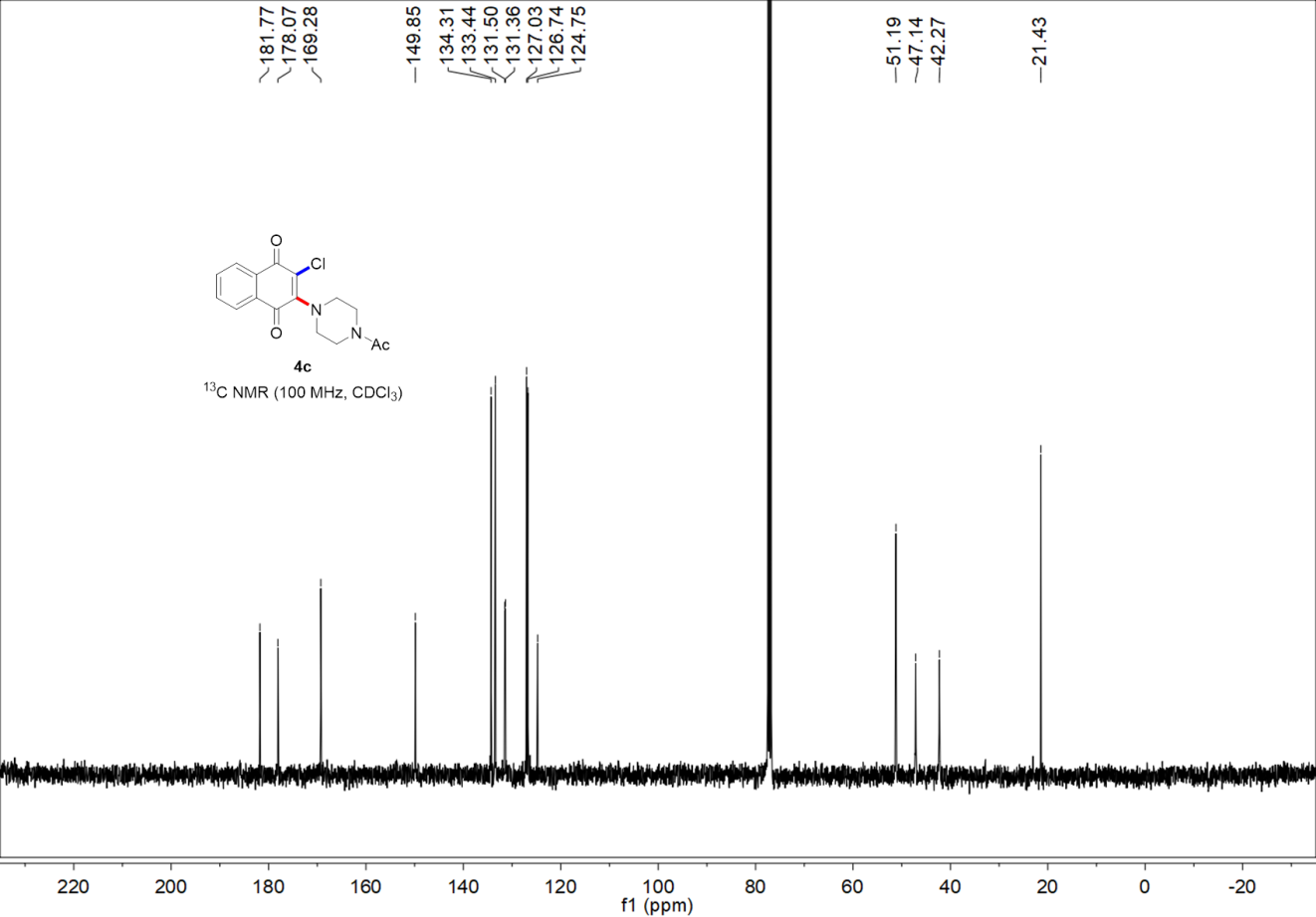
**

**
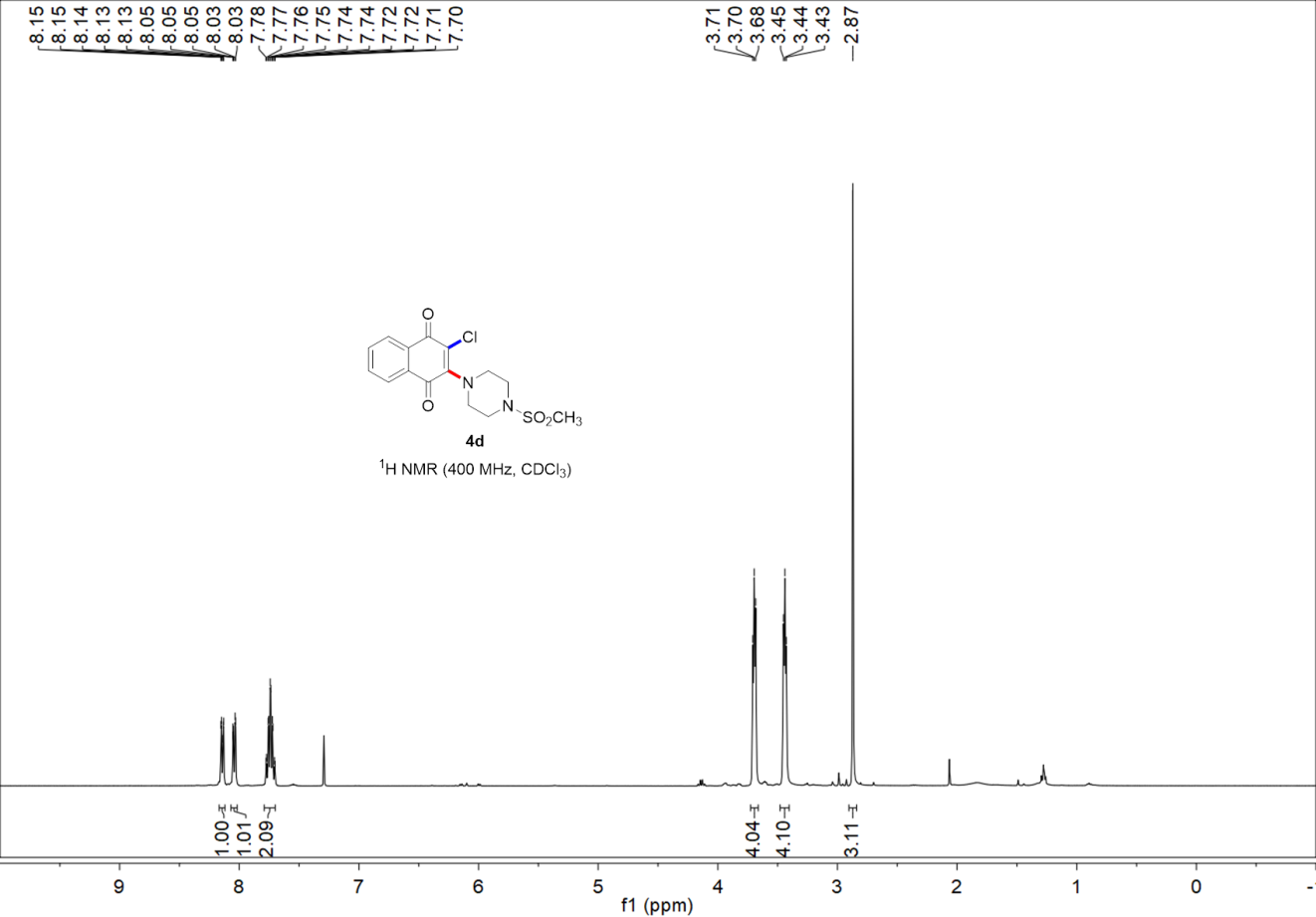
**

**
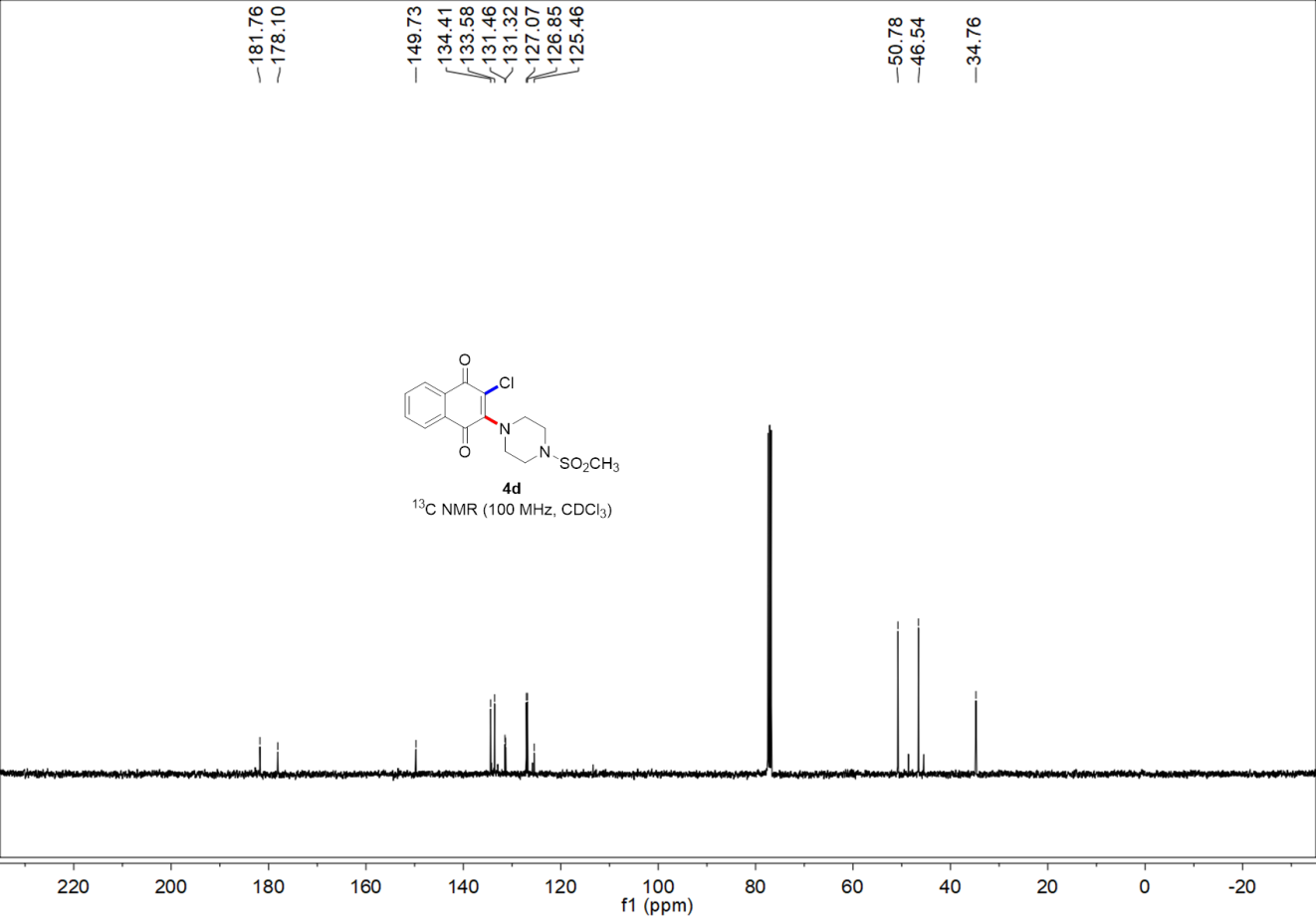
**

**
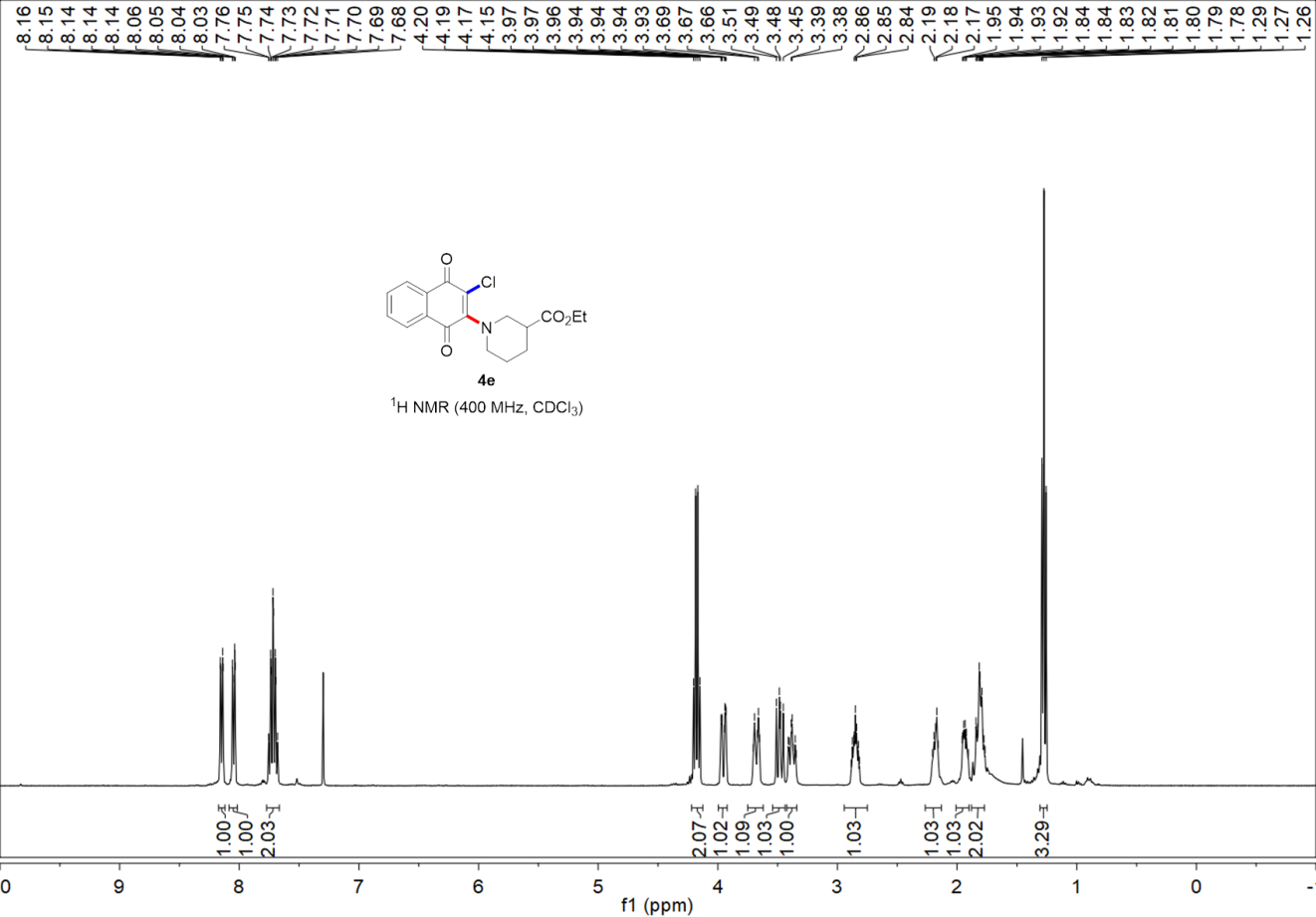
**

**
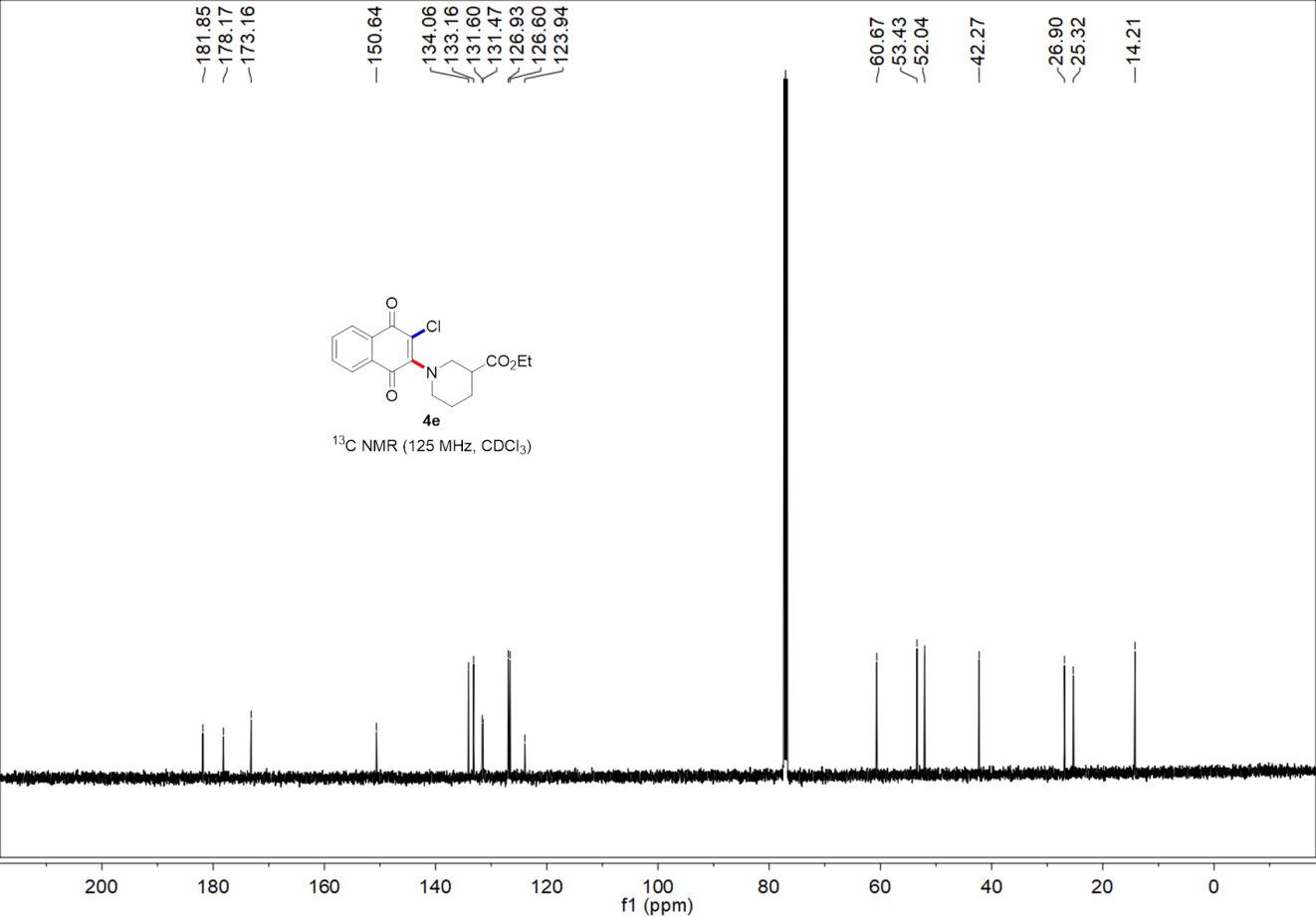
**

**
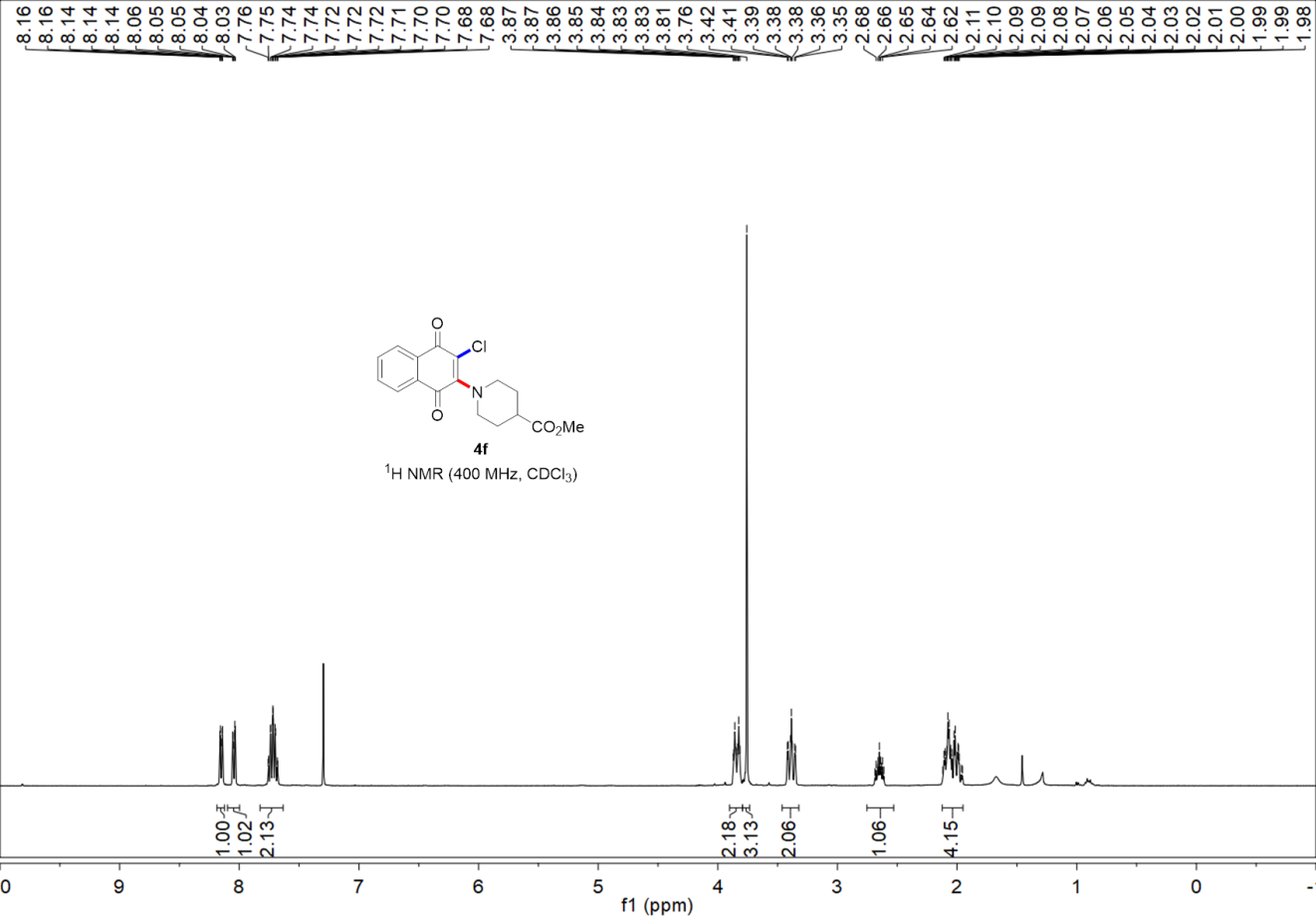
**

**
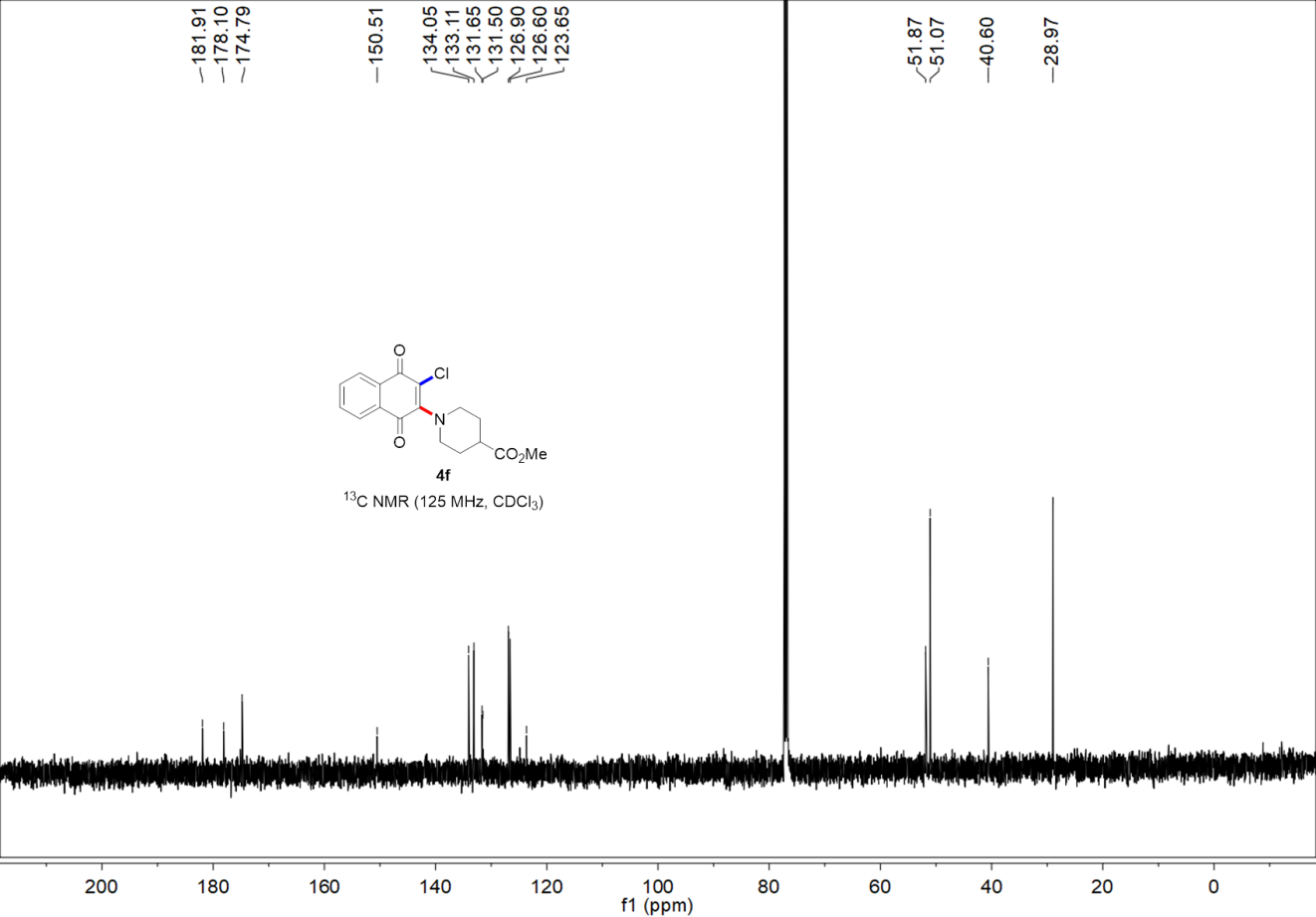
**

**
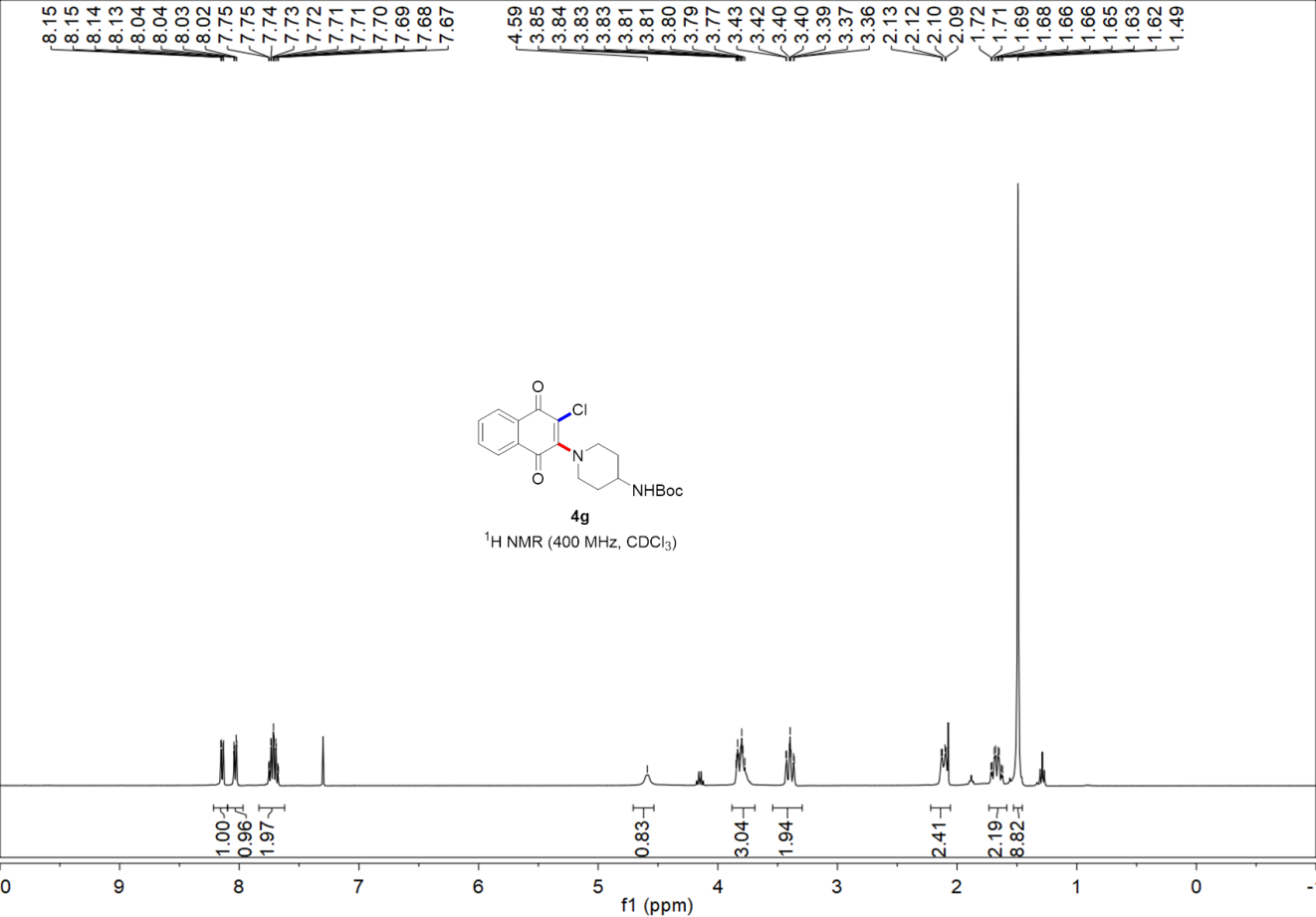
**

**
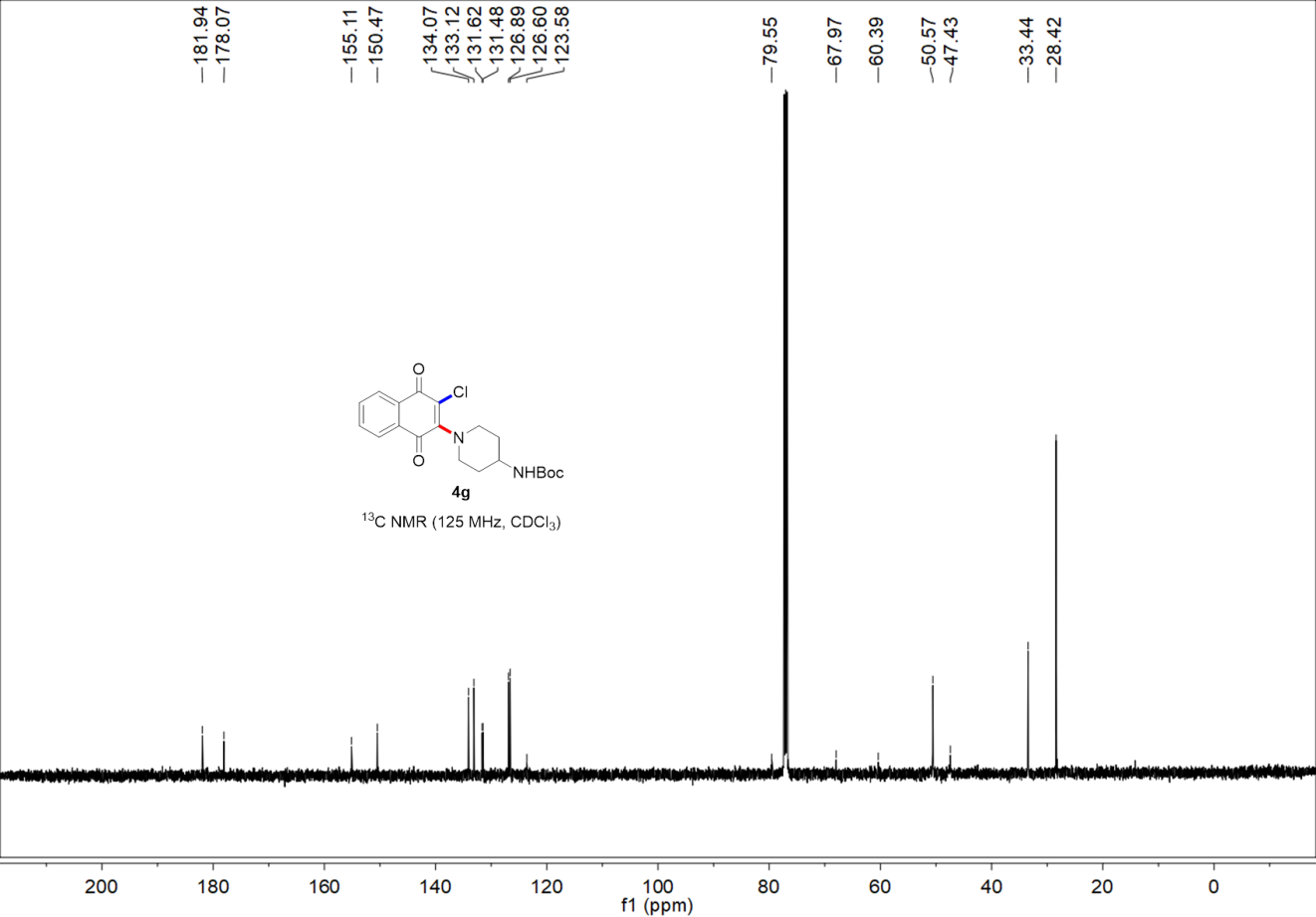
**

**
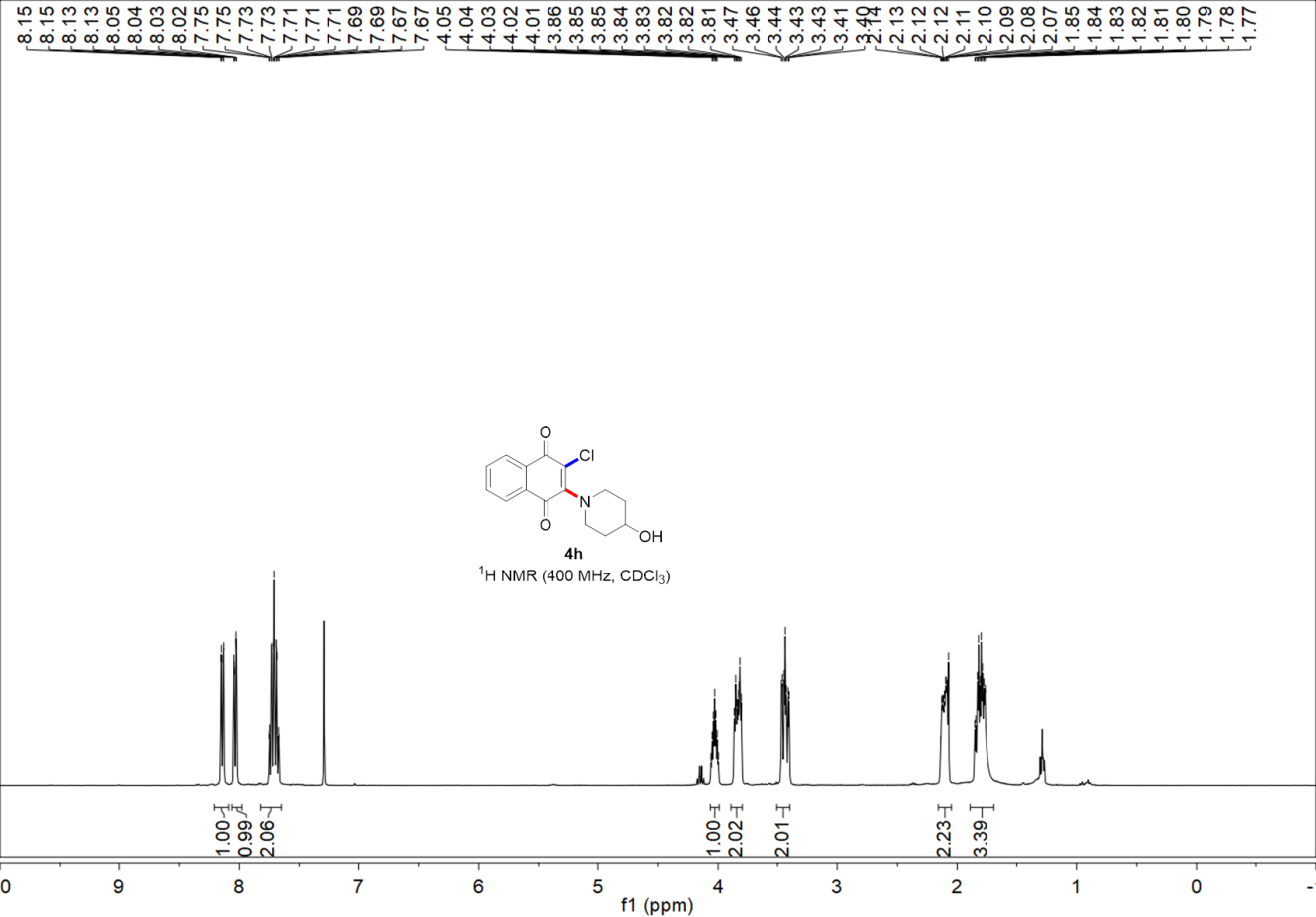
**

**
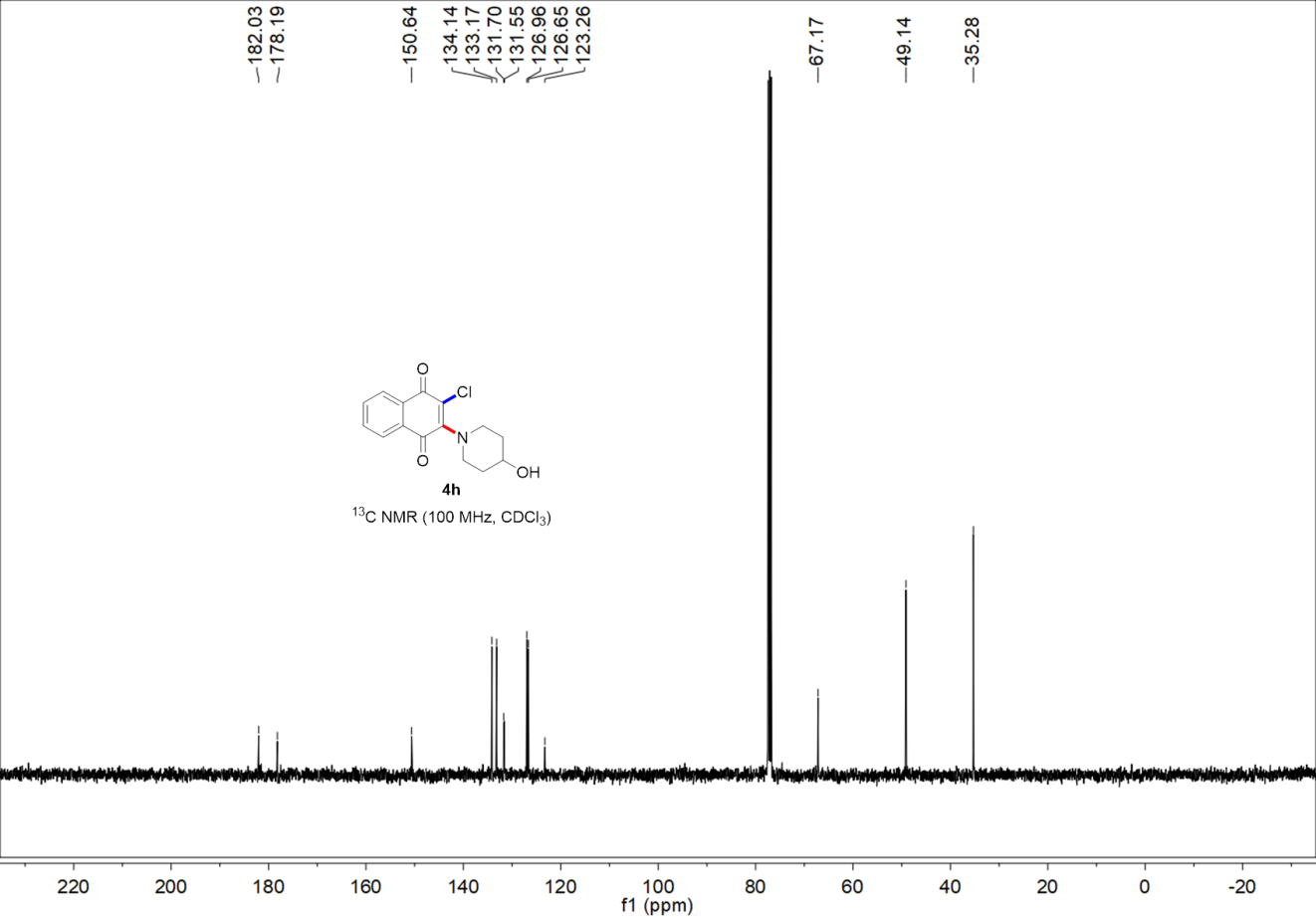
**

**
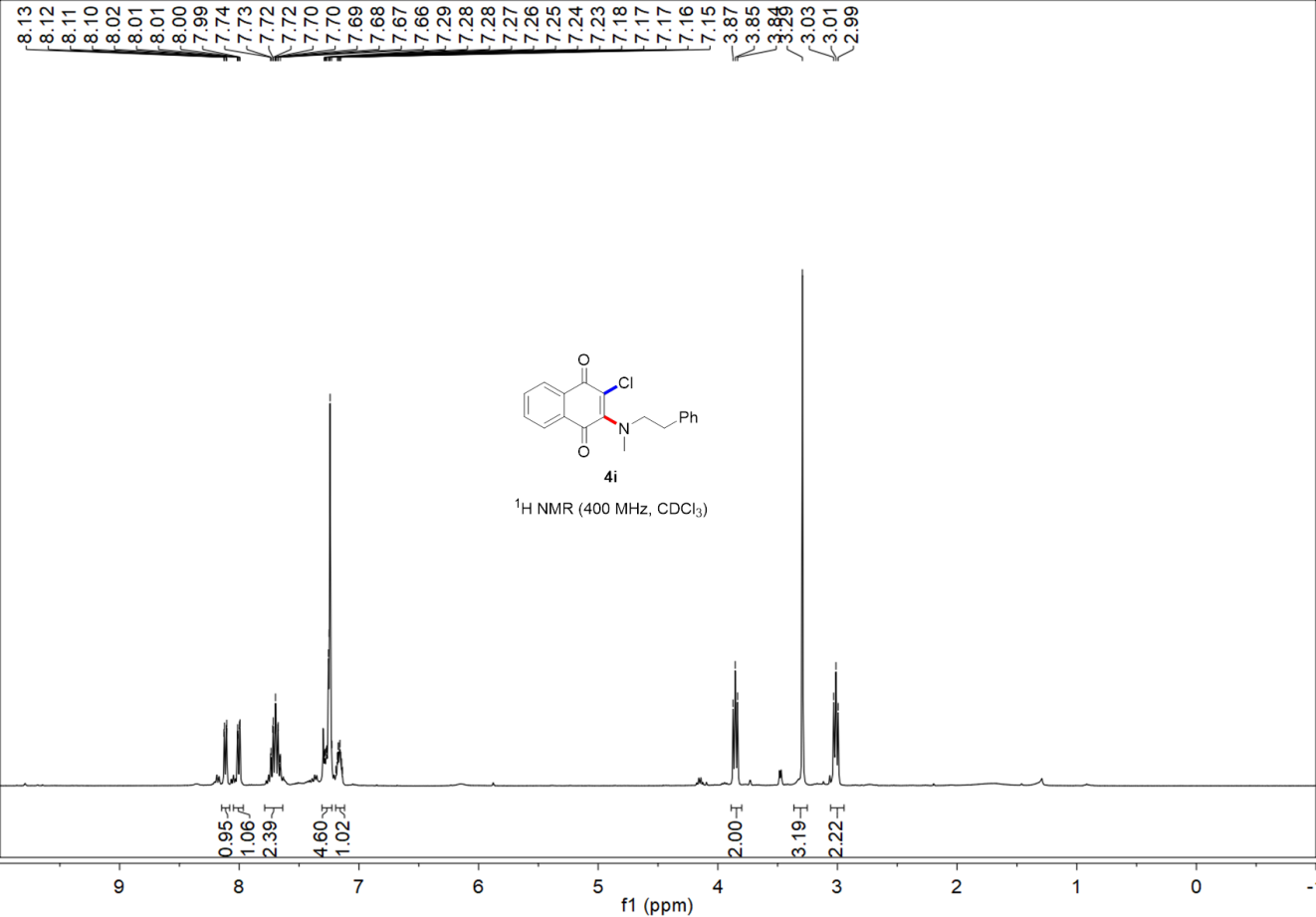
**

**
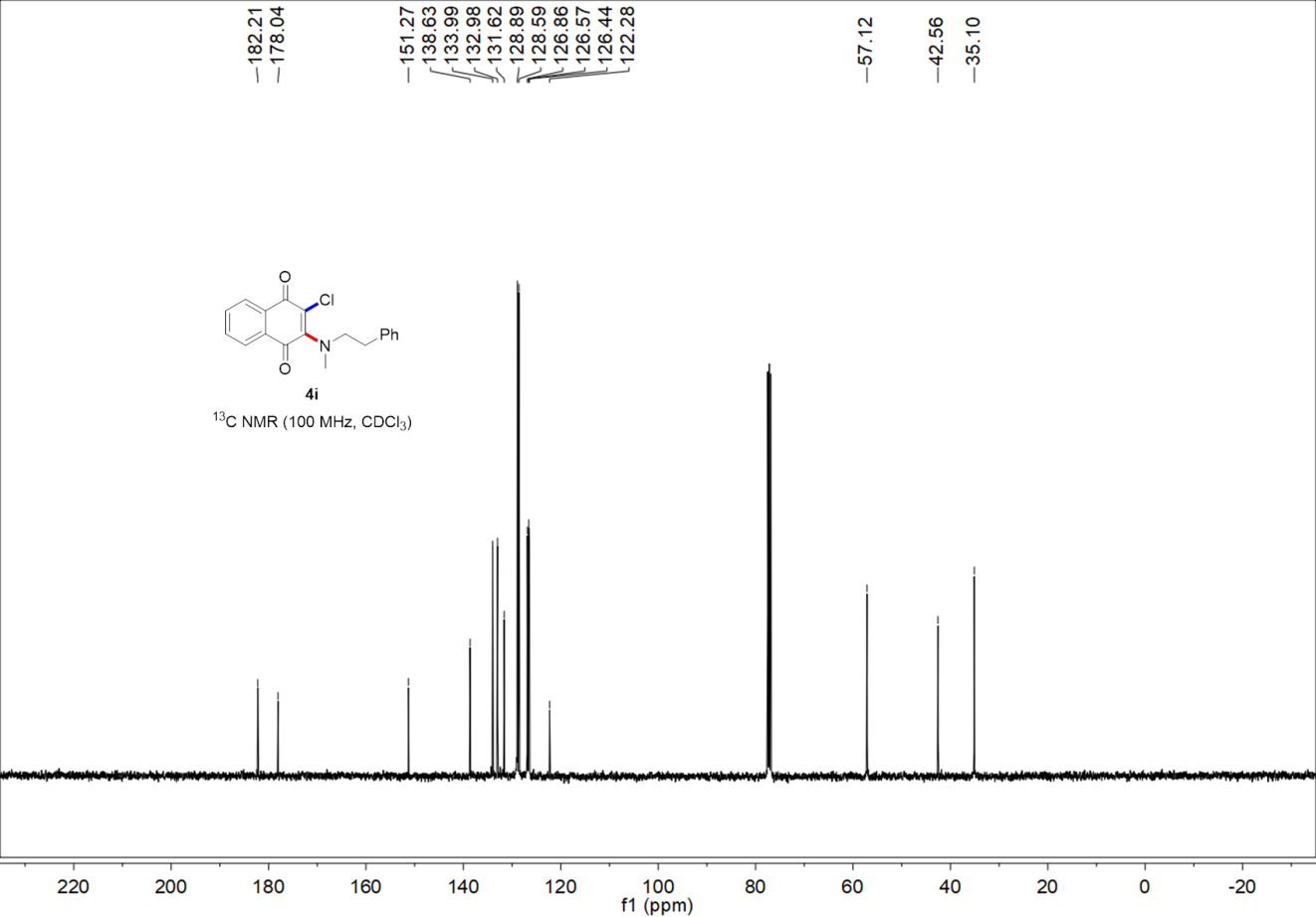
**

**
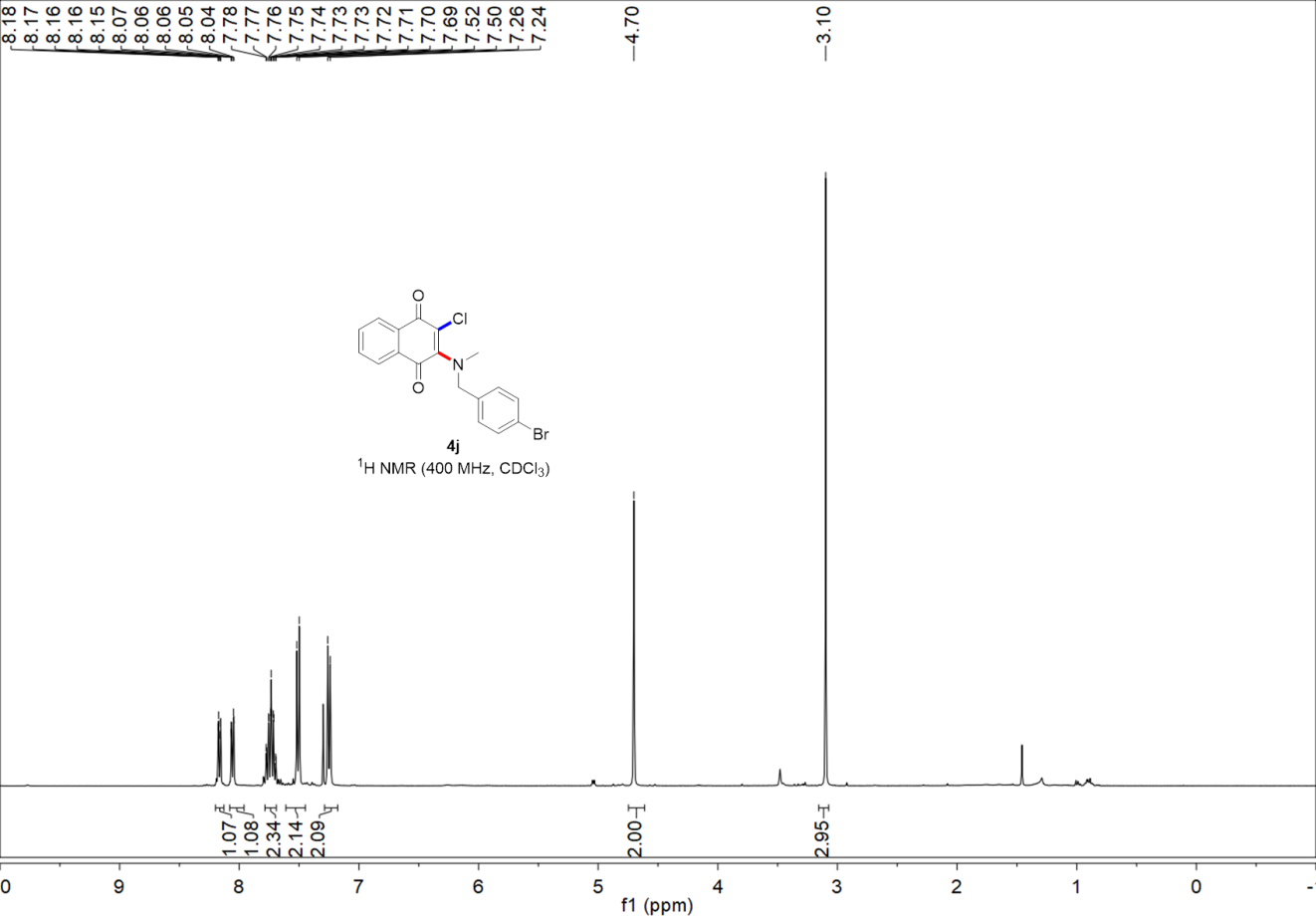
**

**
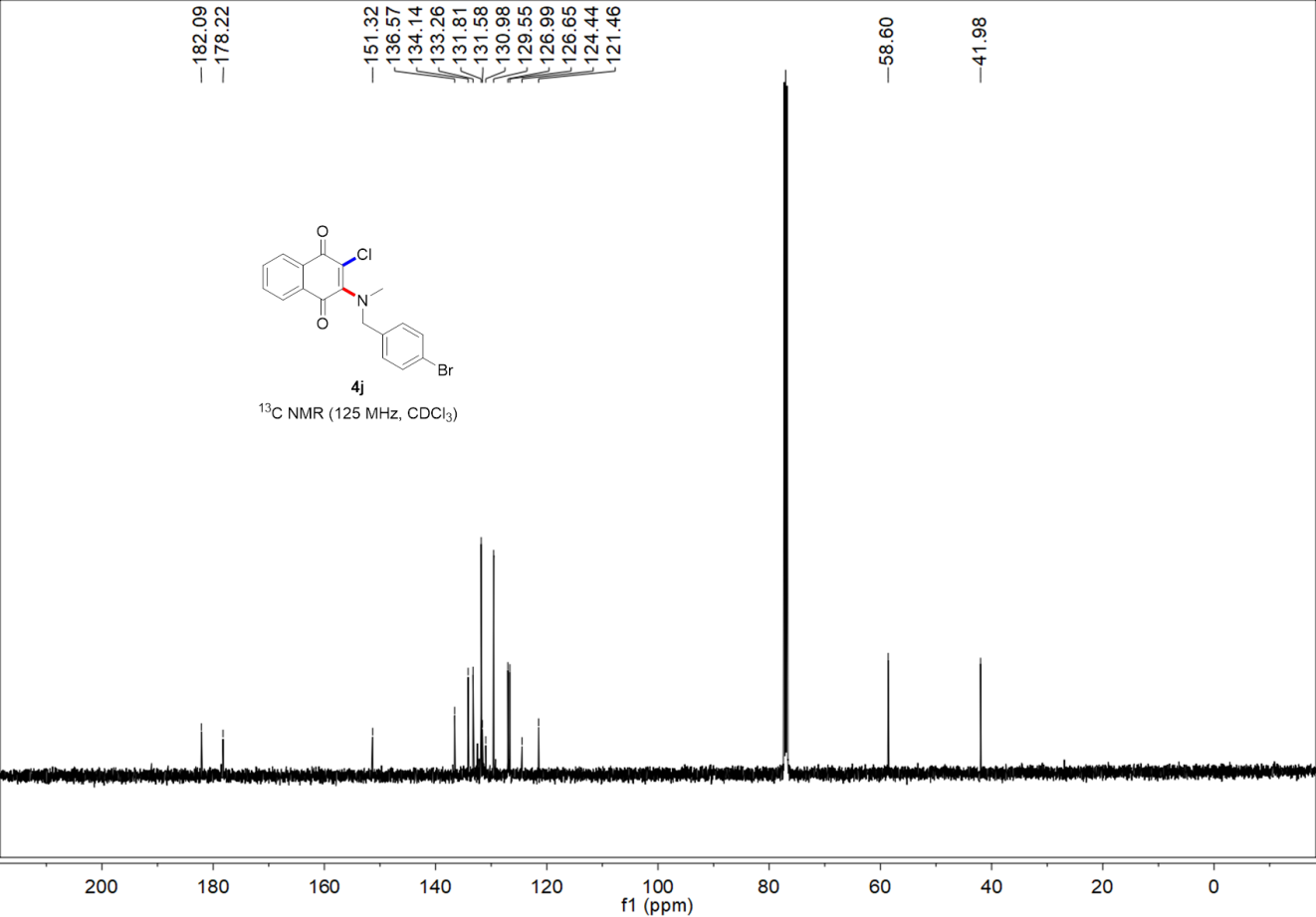
**

**
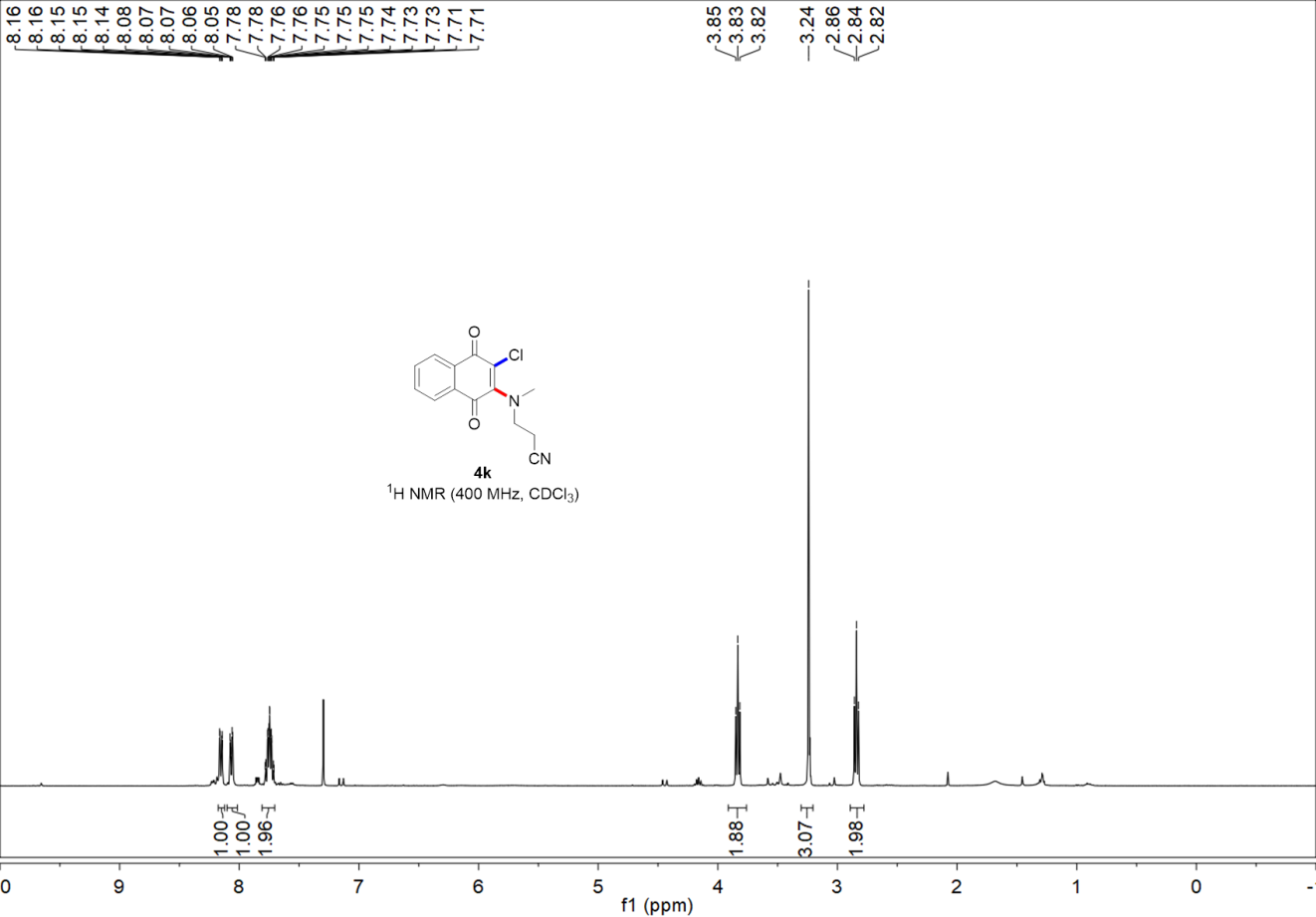
**

**
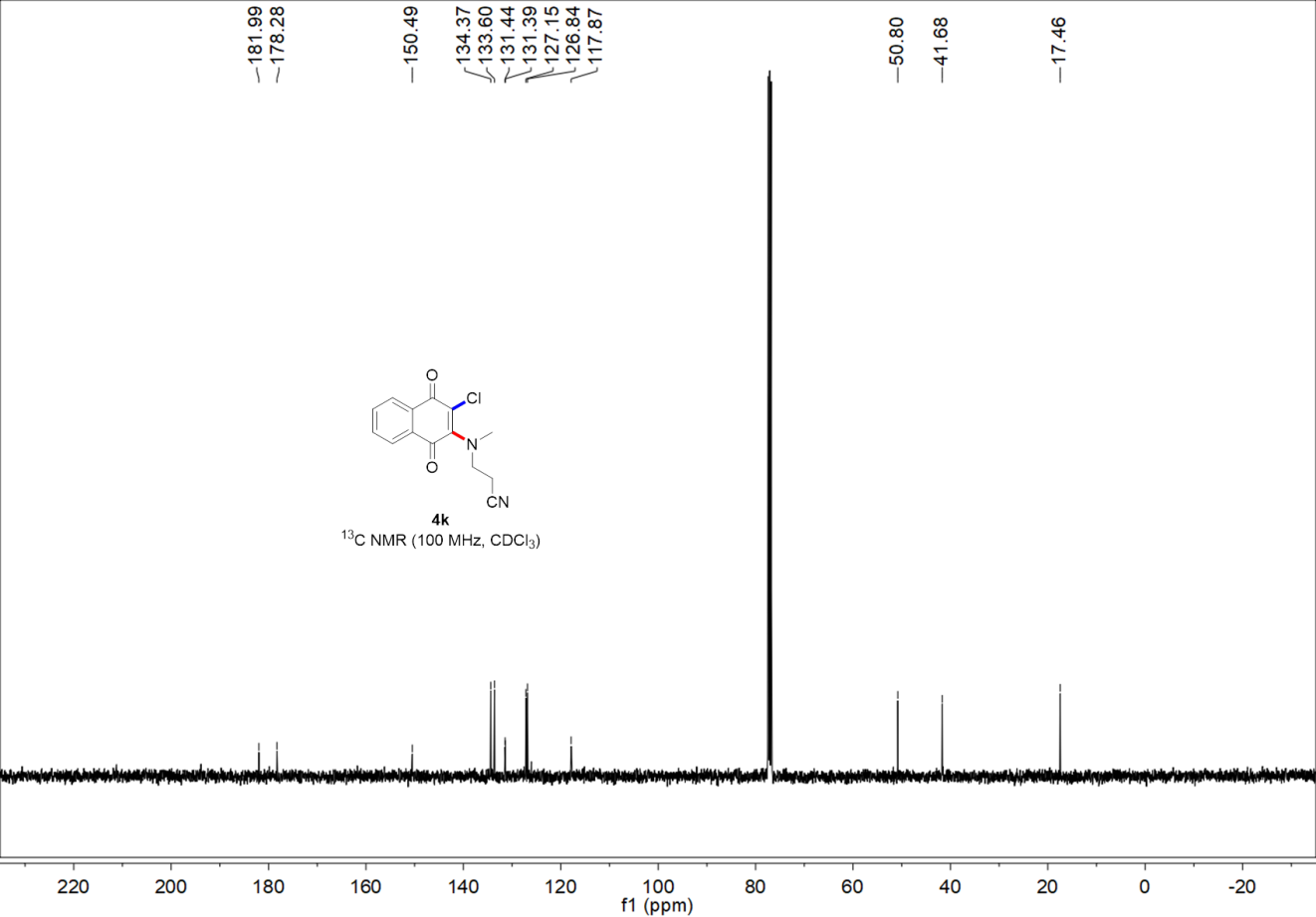
**

**
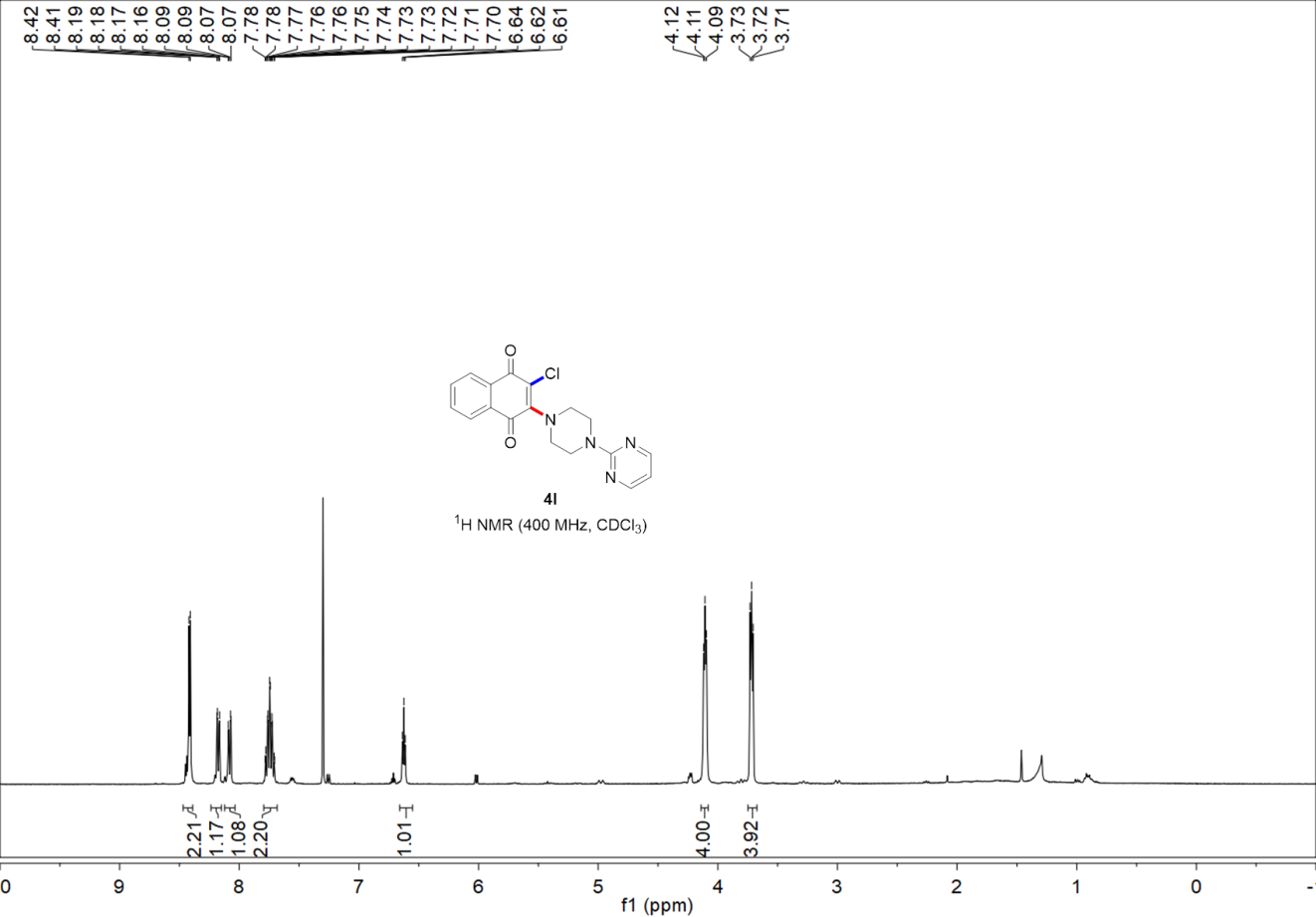
**

**
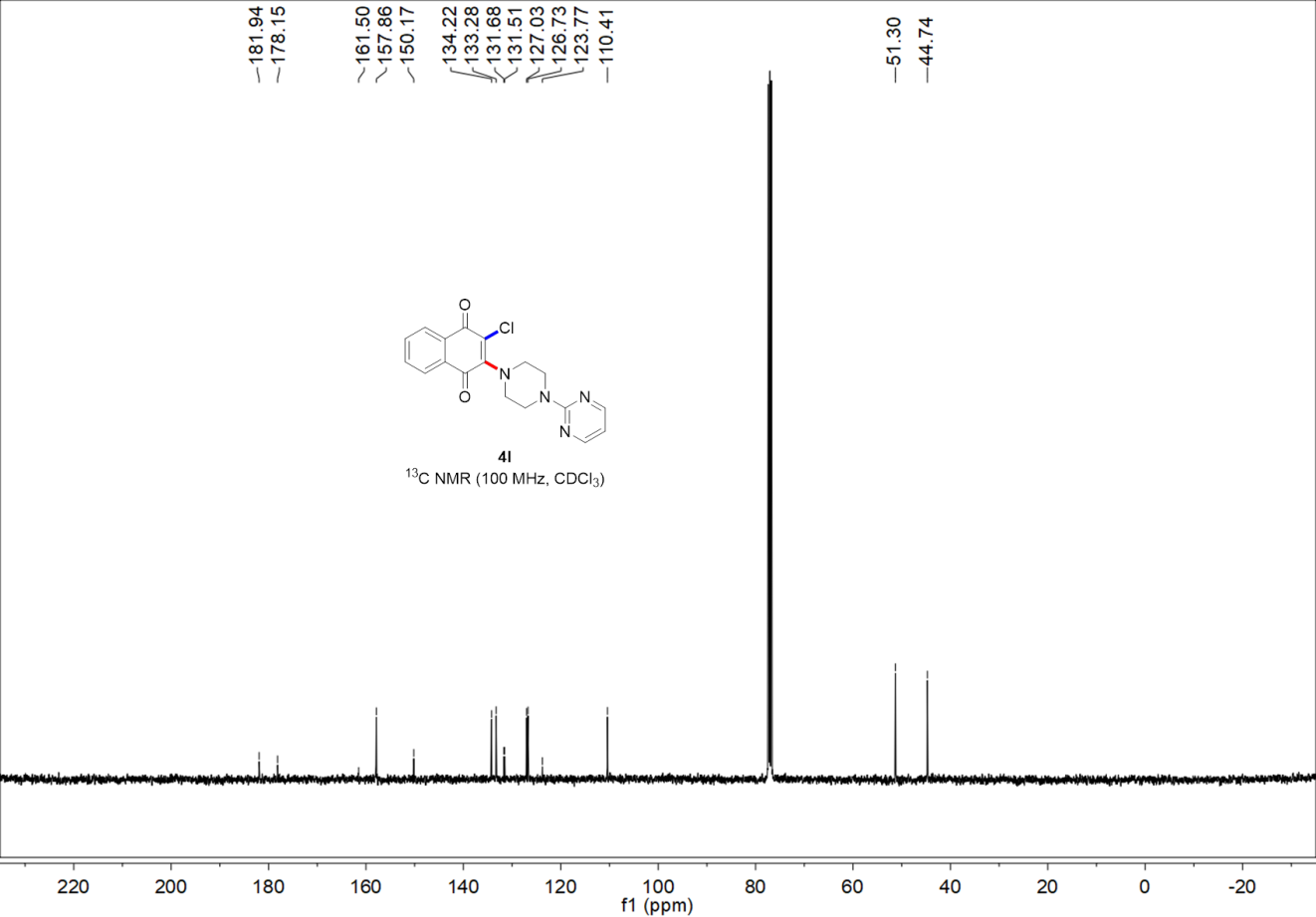
**

**
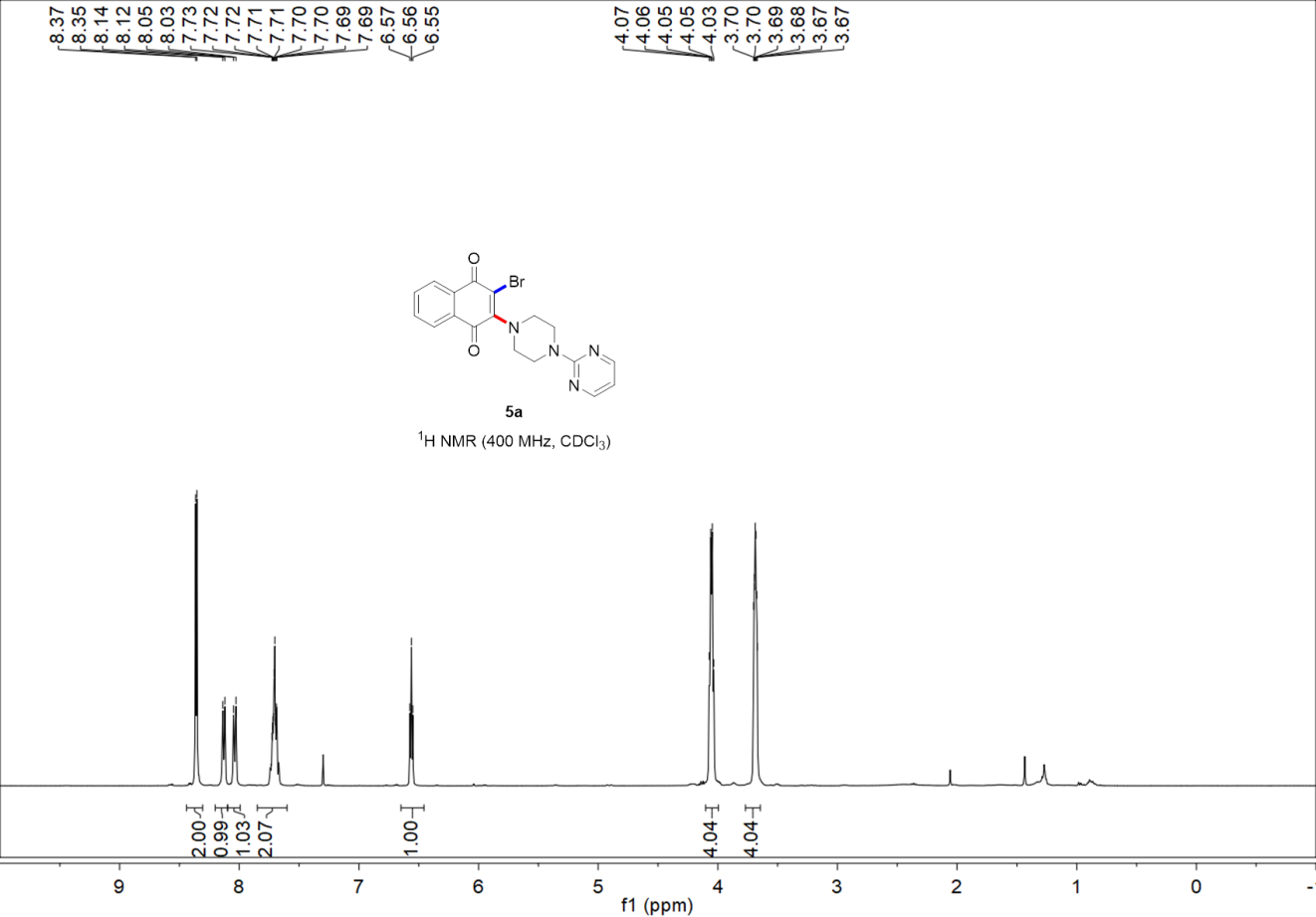
**

**
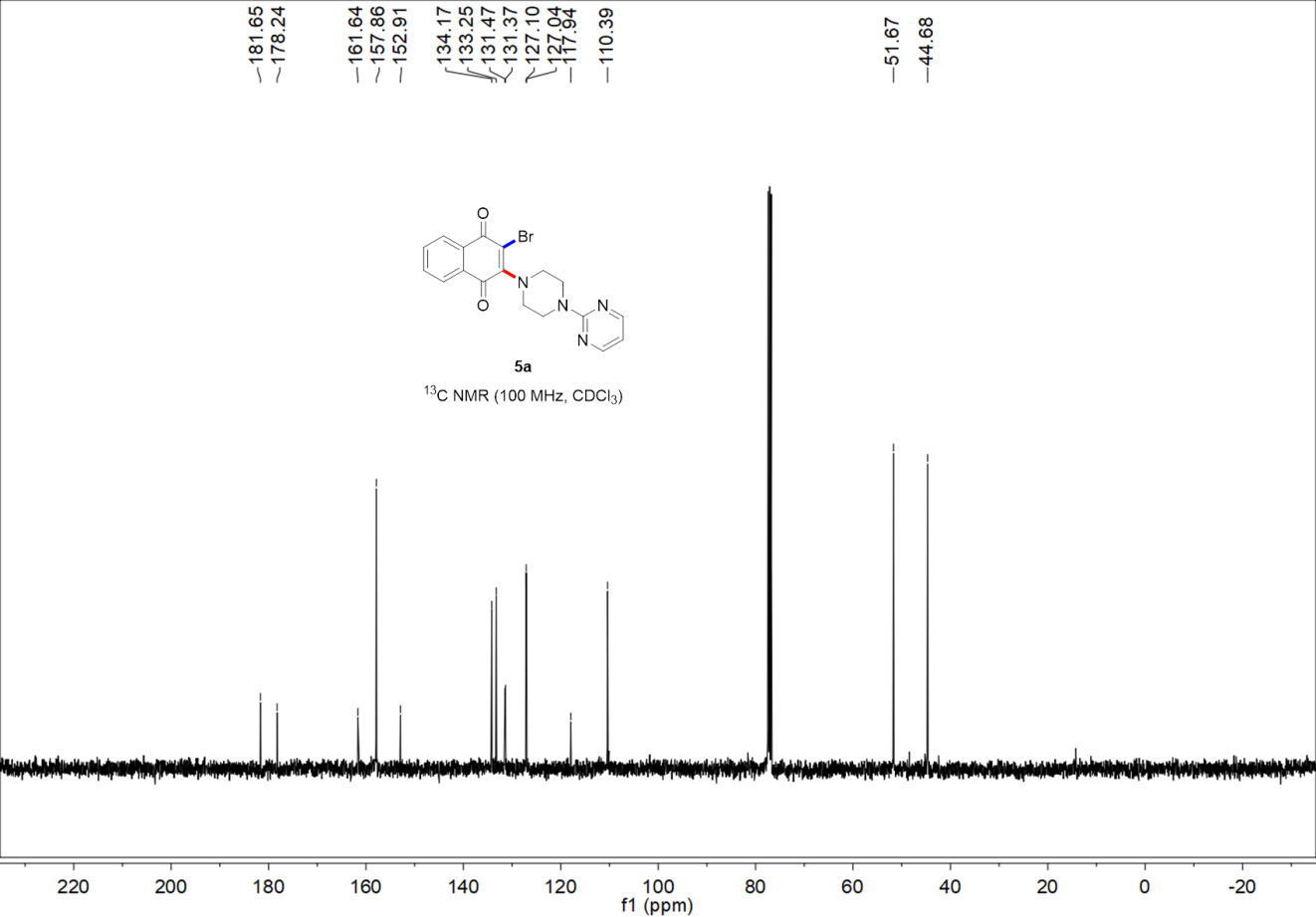
**

**
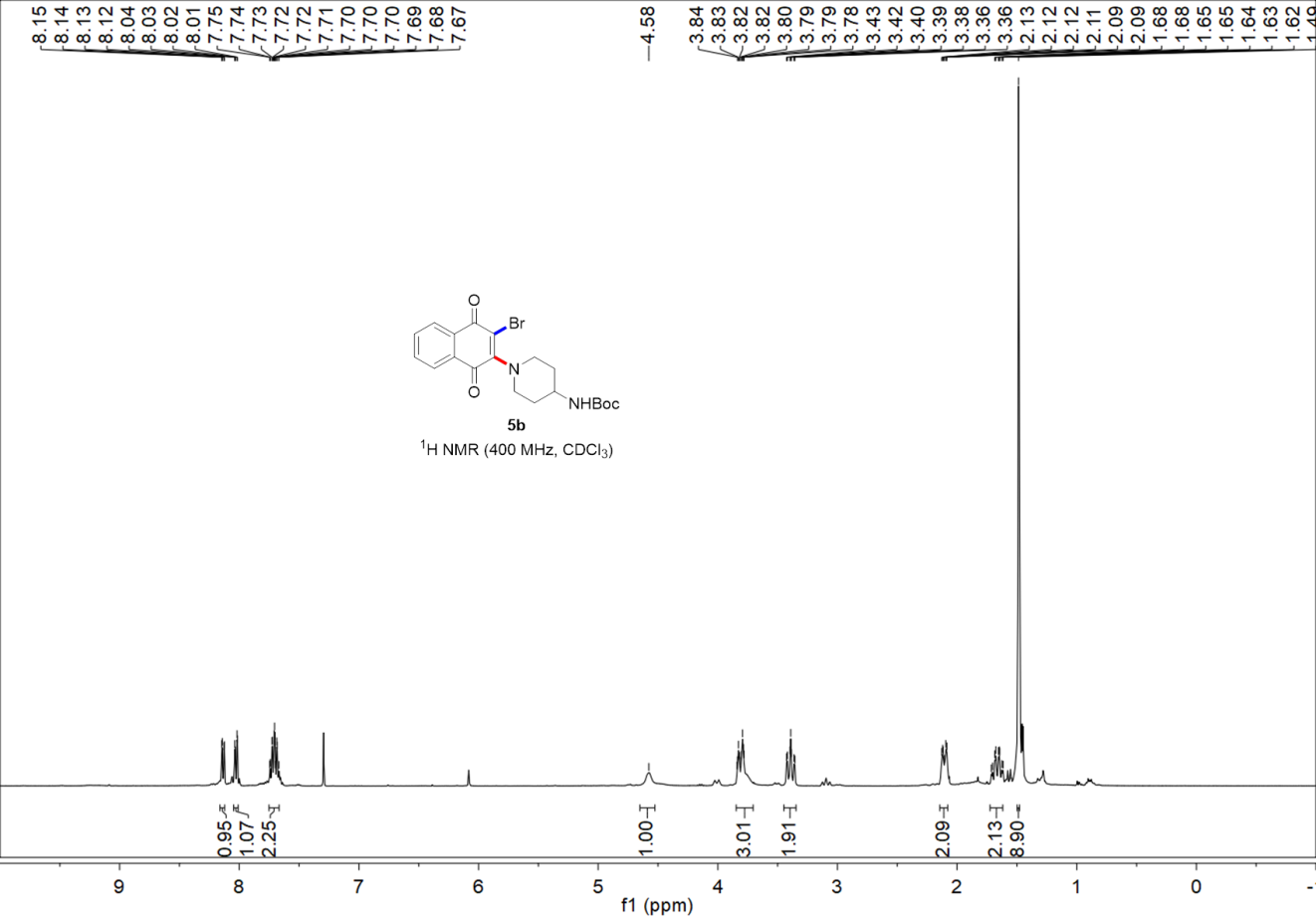
**

**
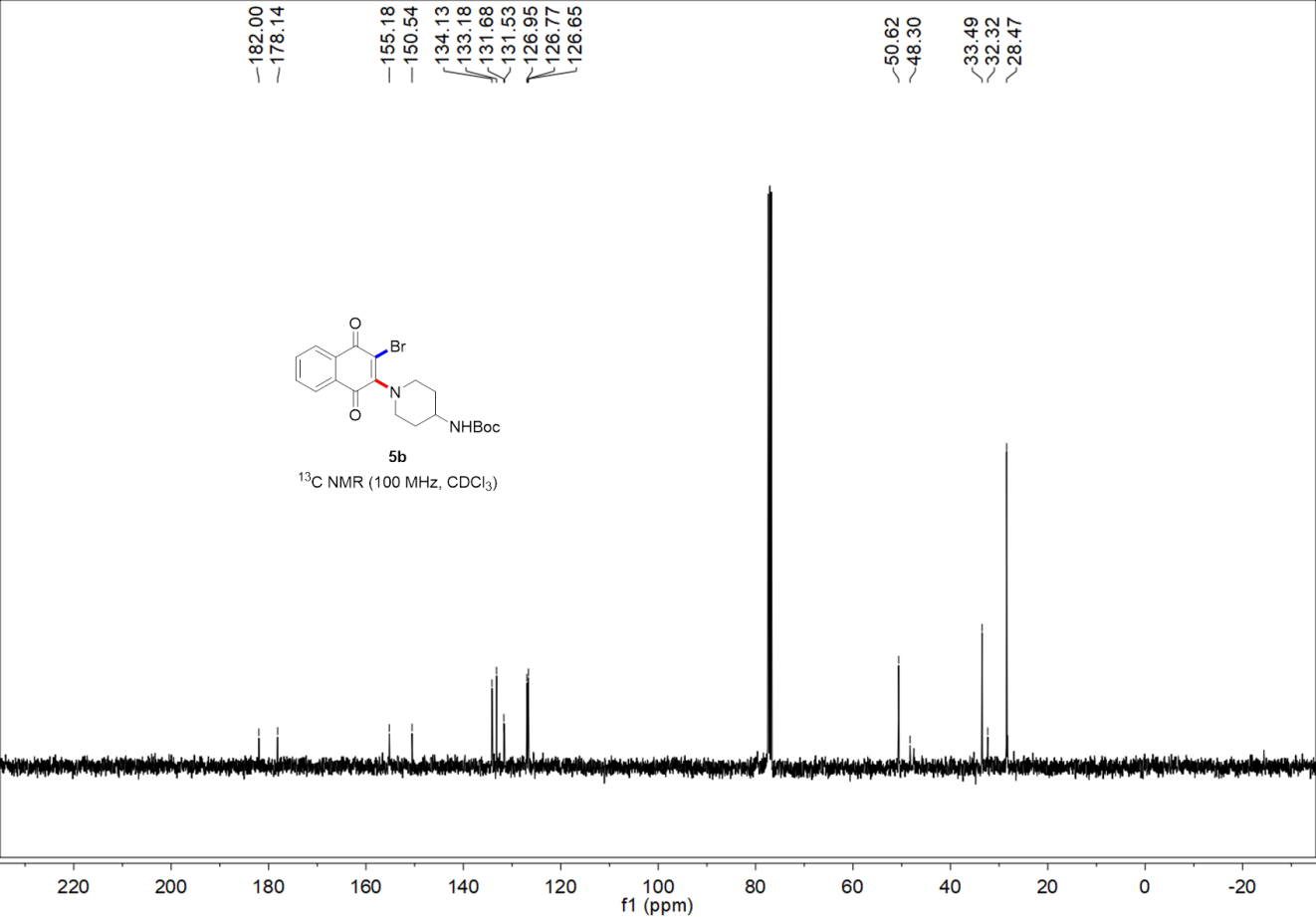
**

**
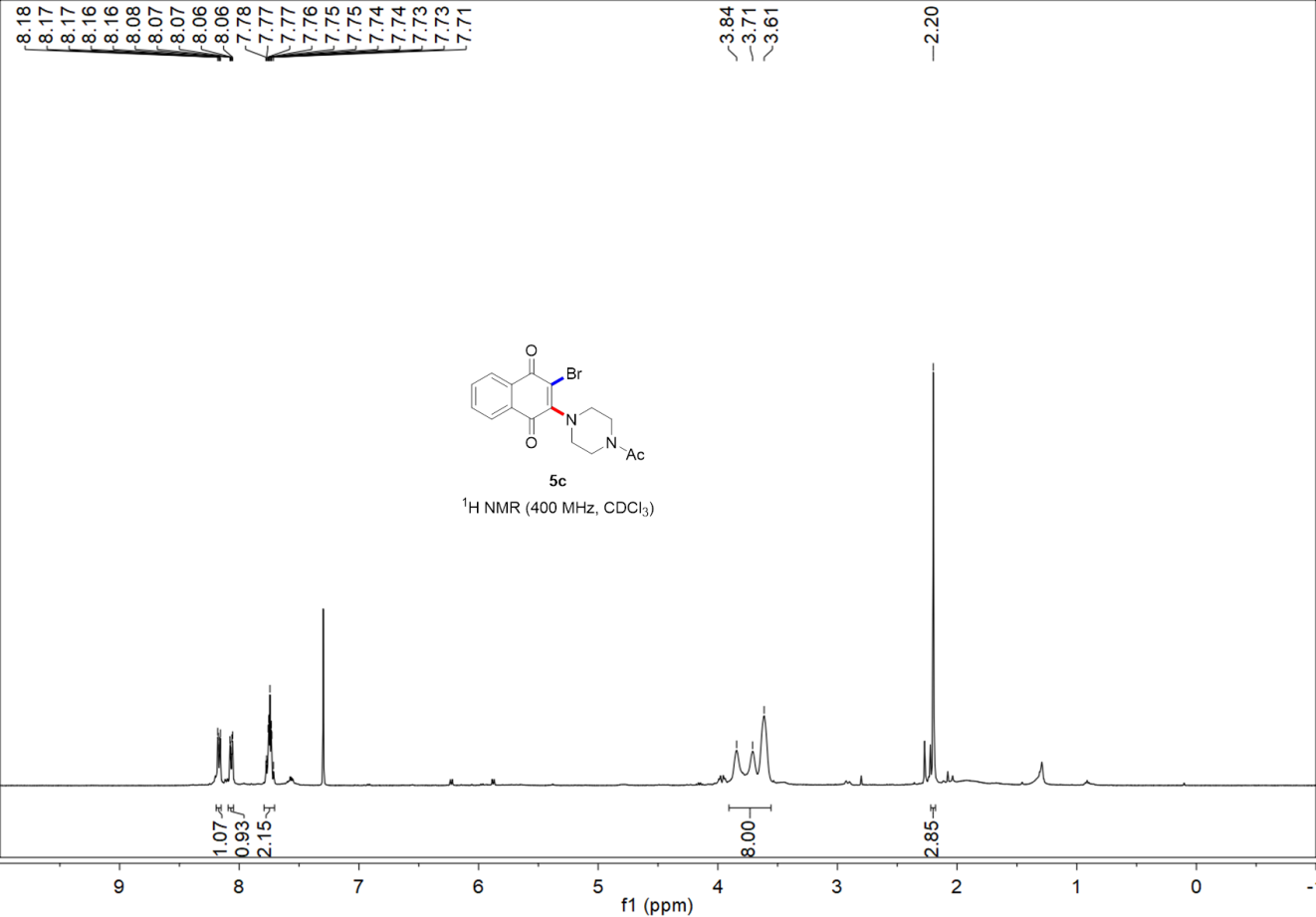
**

**
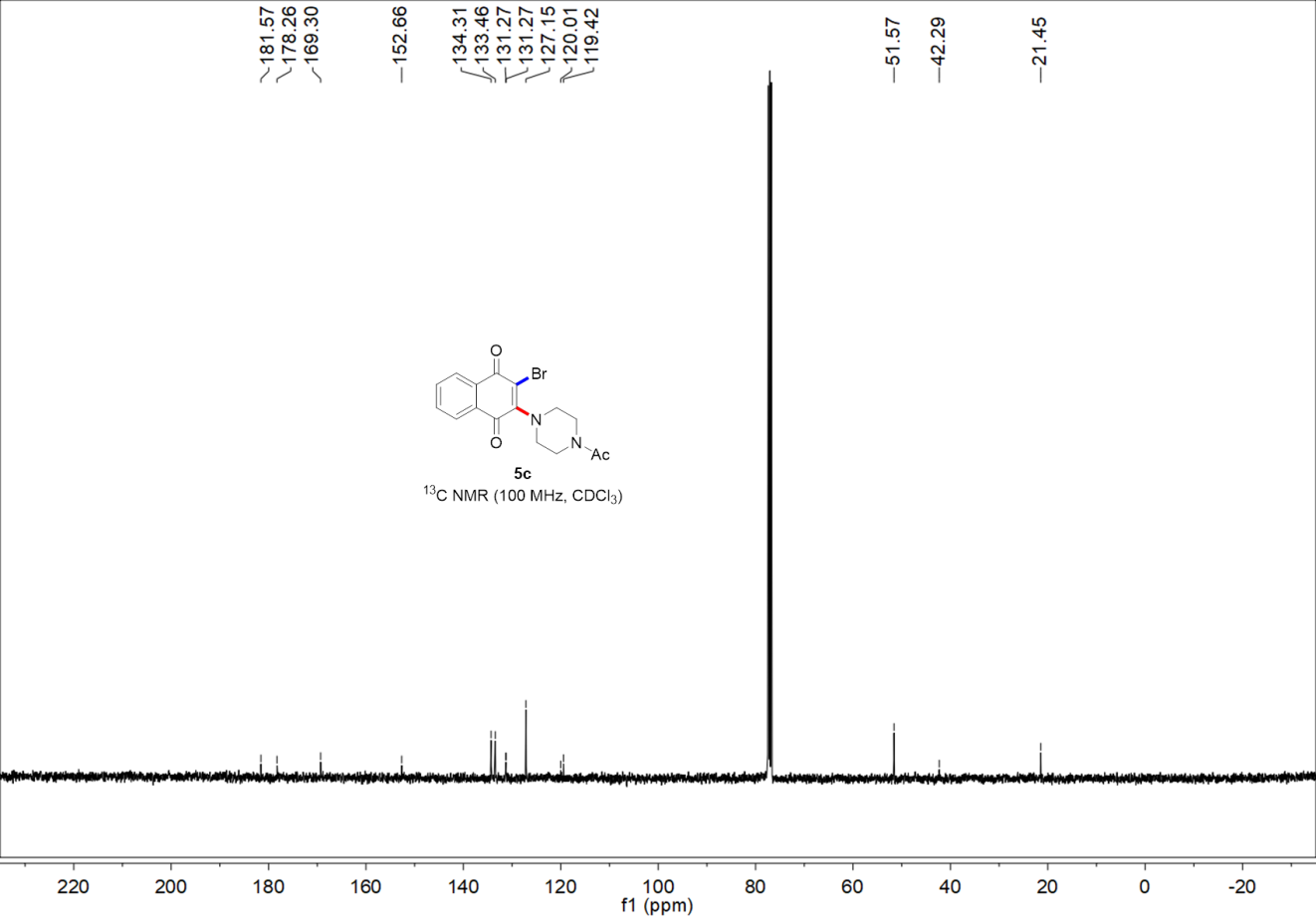
**

**
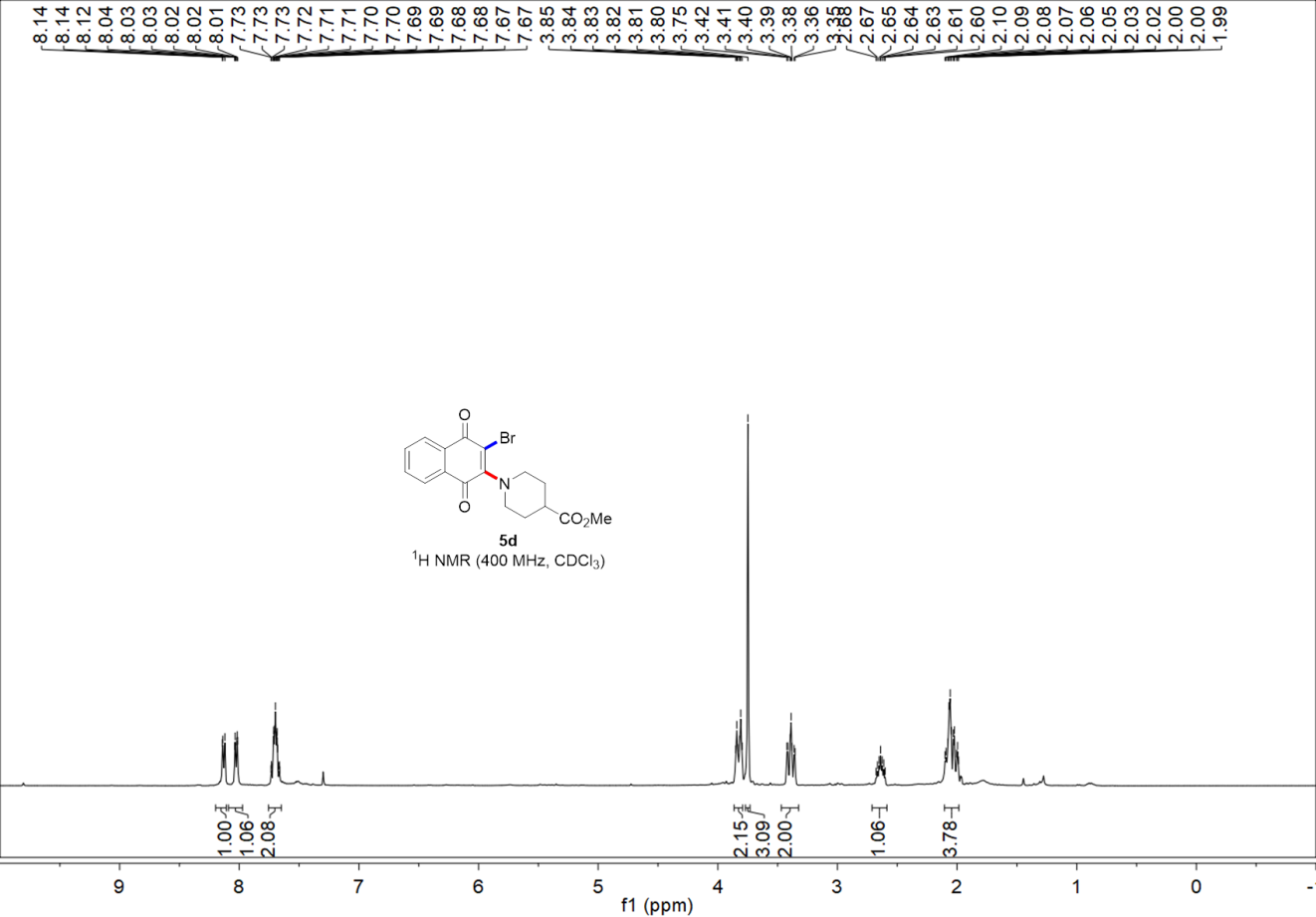
**

**
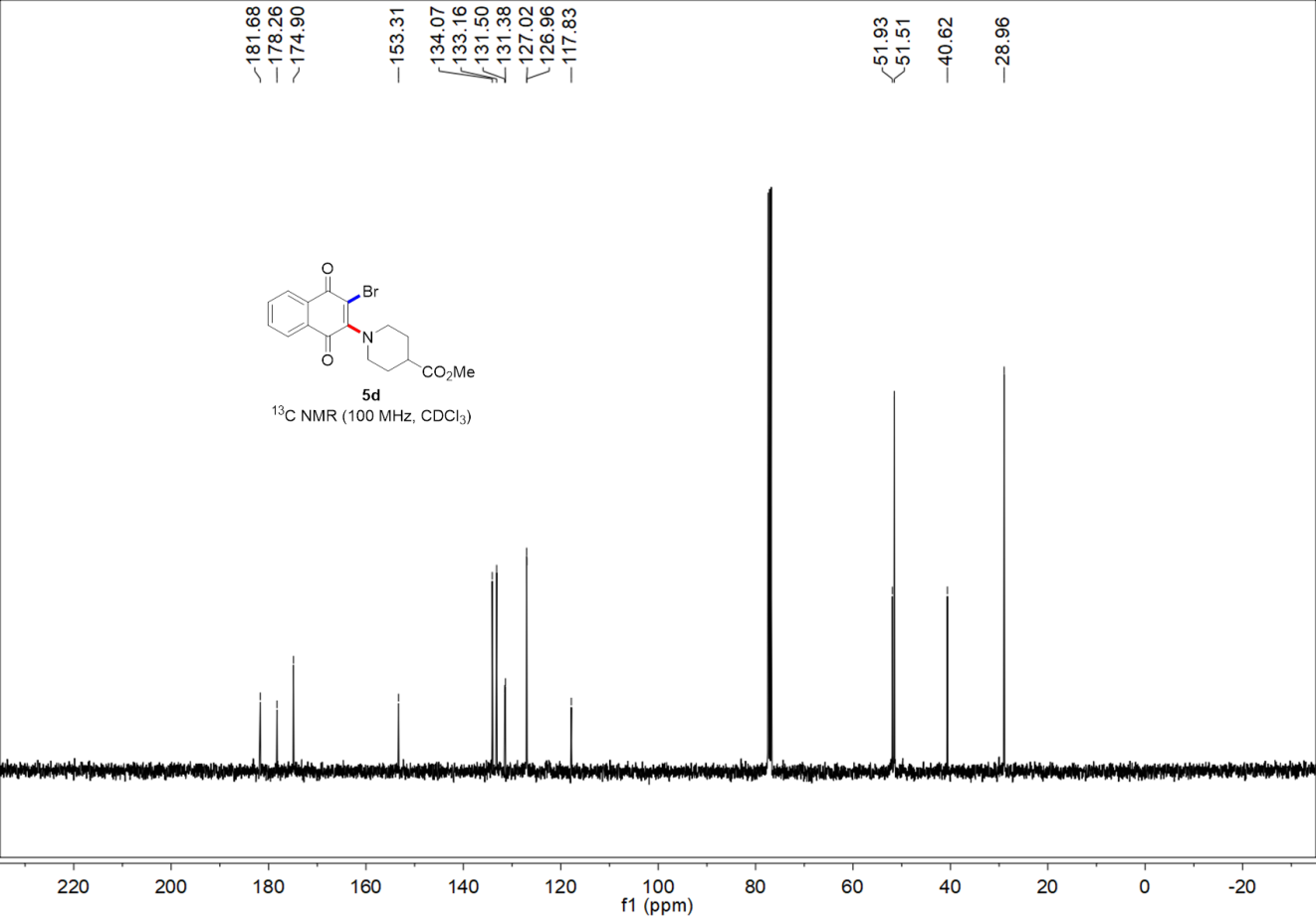
**

**
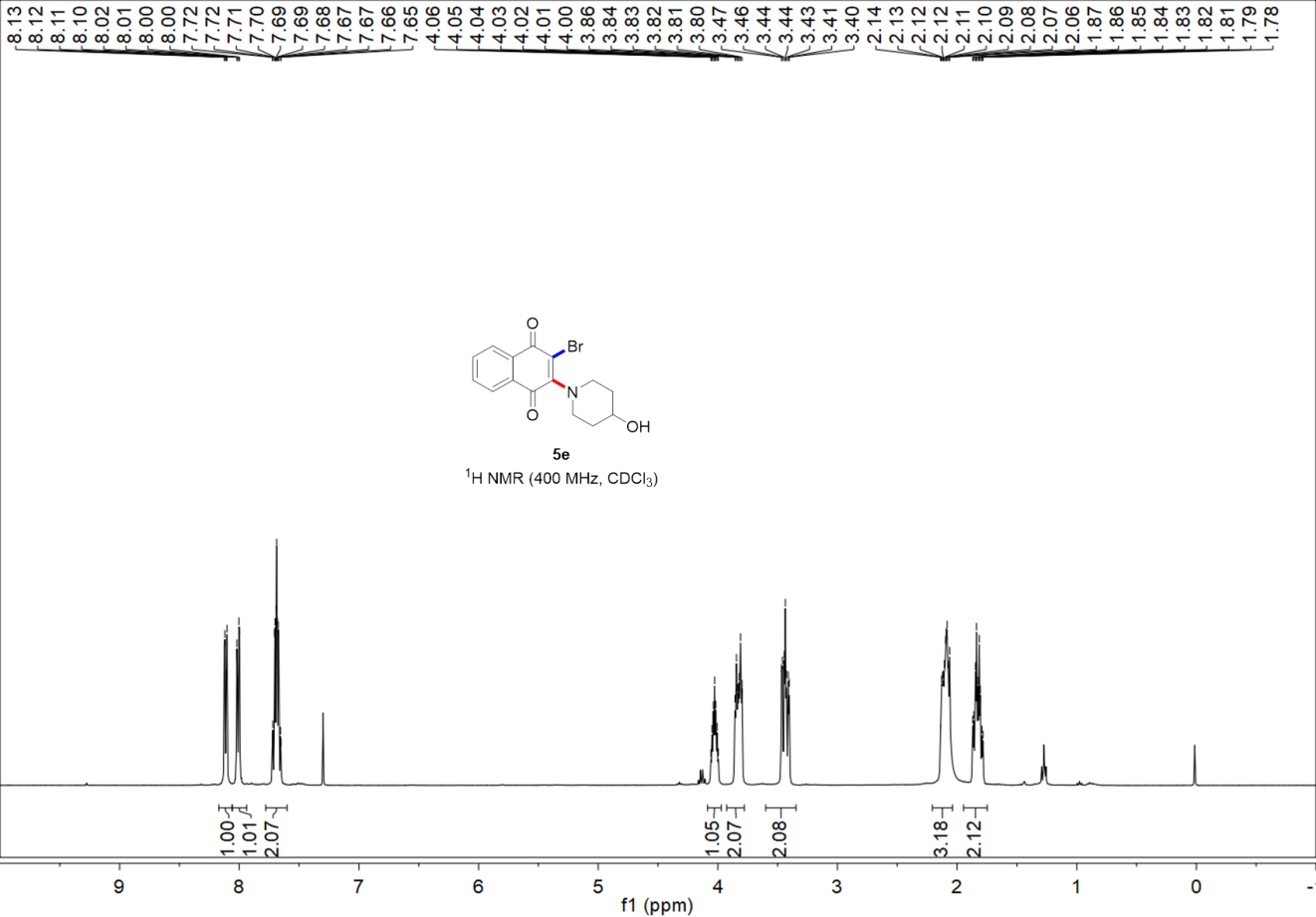
**

**
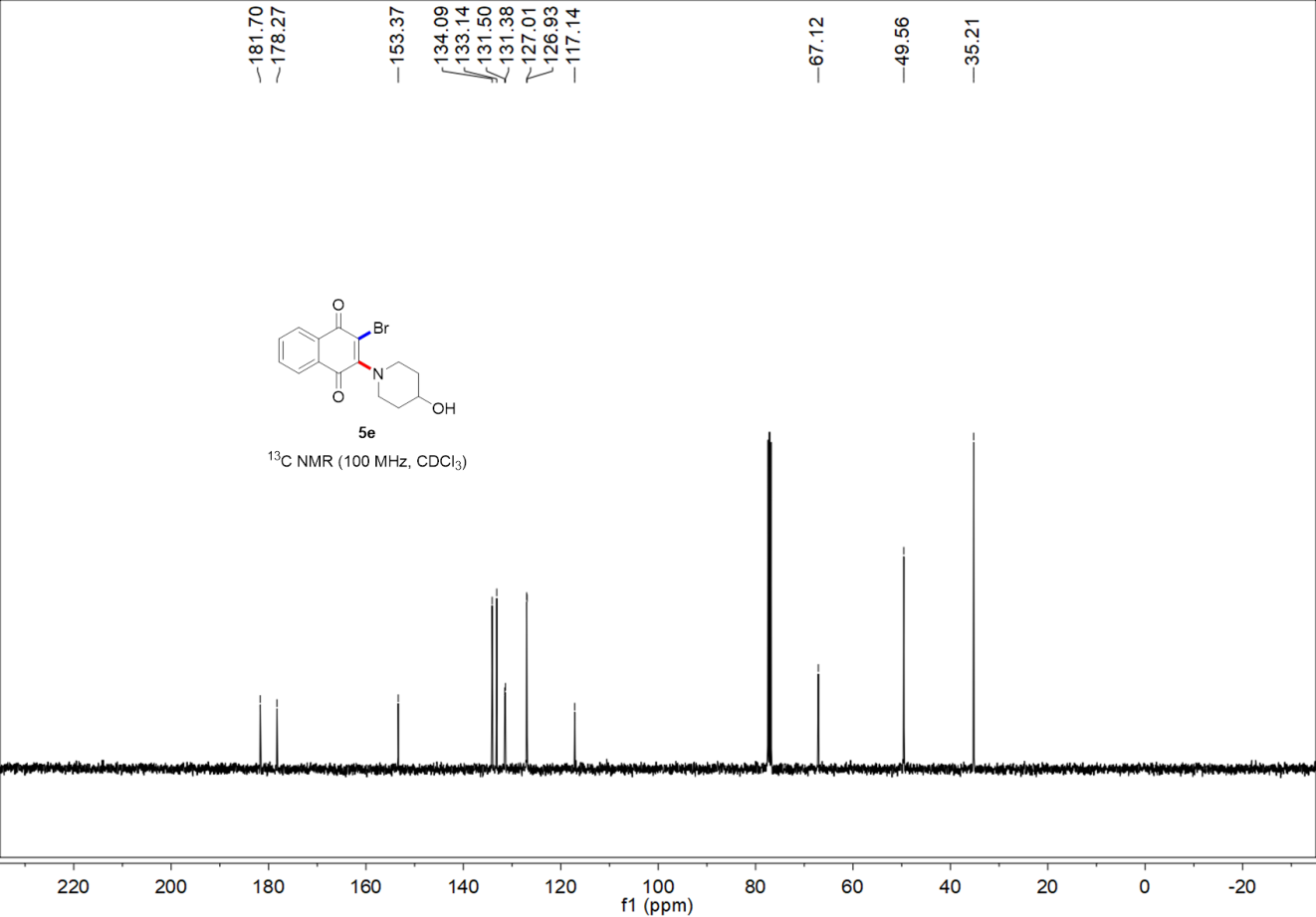
**

**
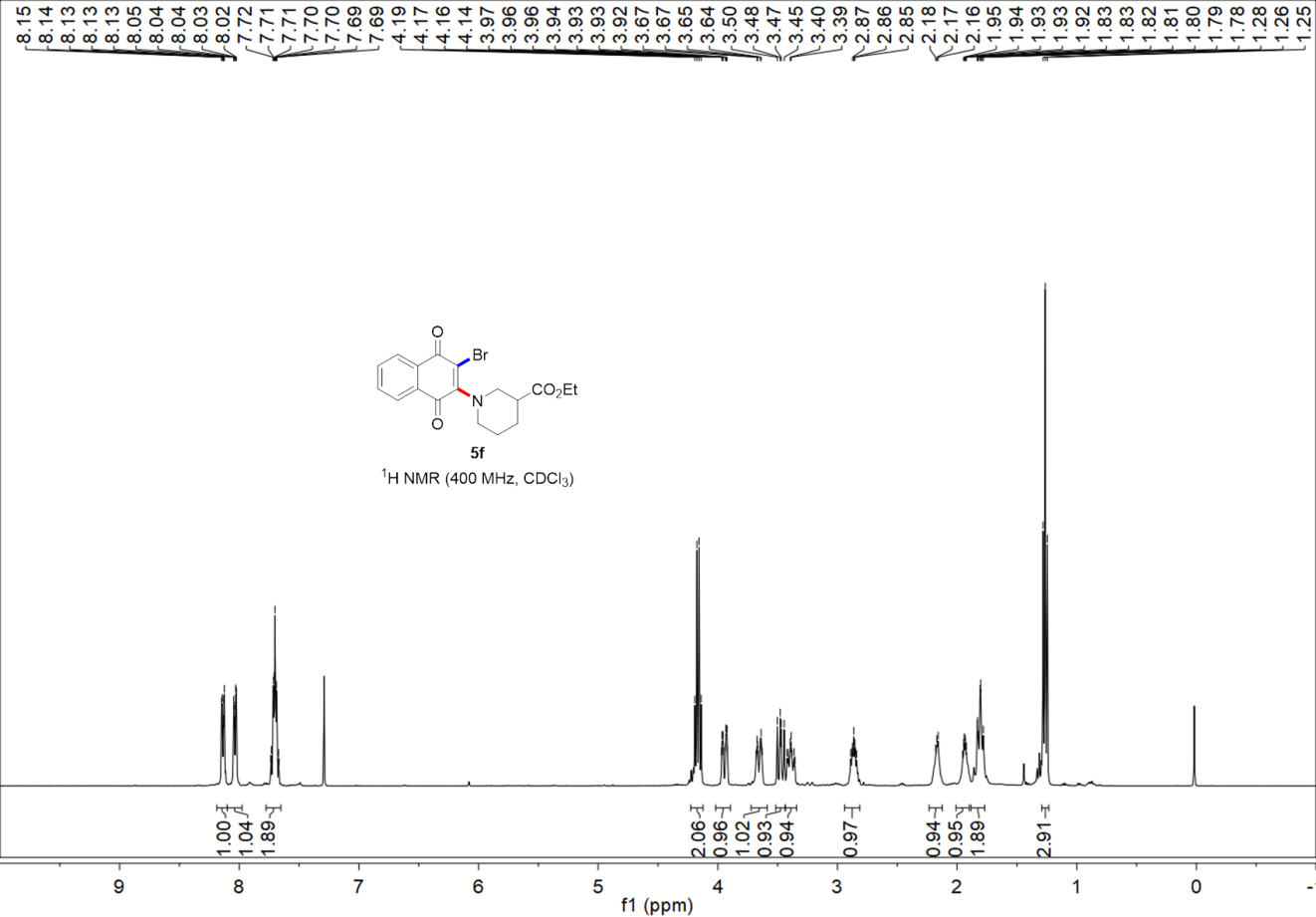
**

**
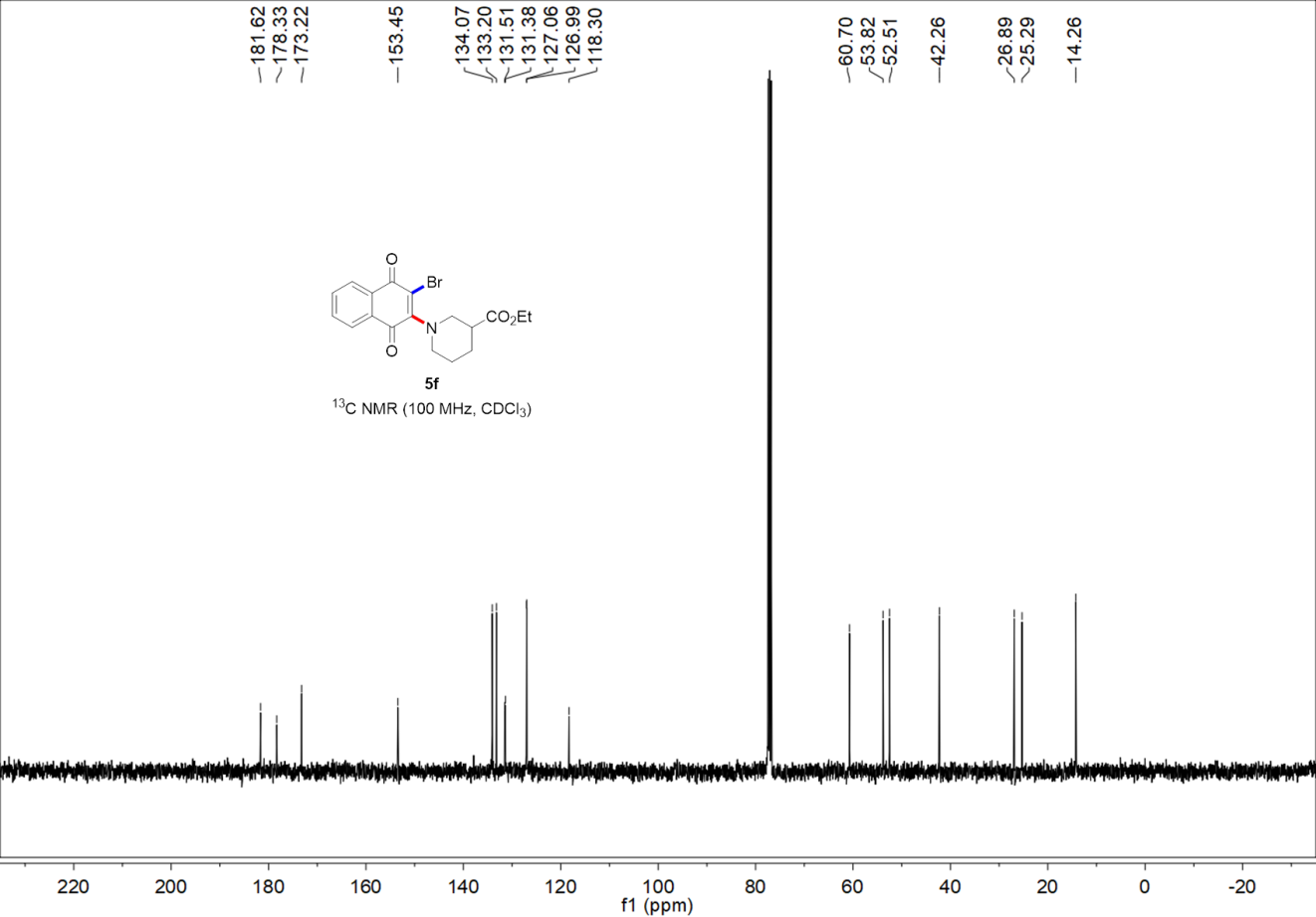
**

**
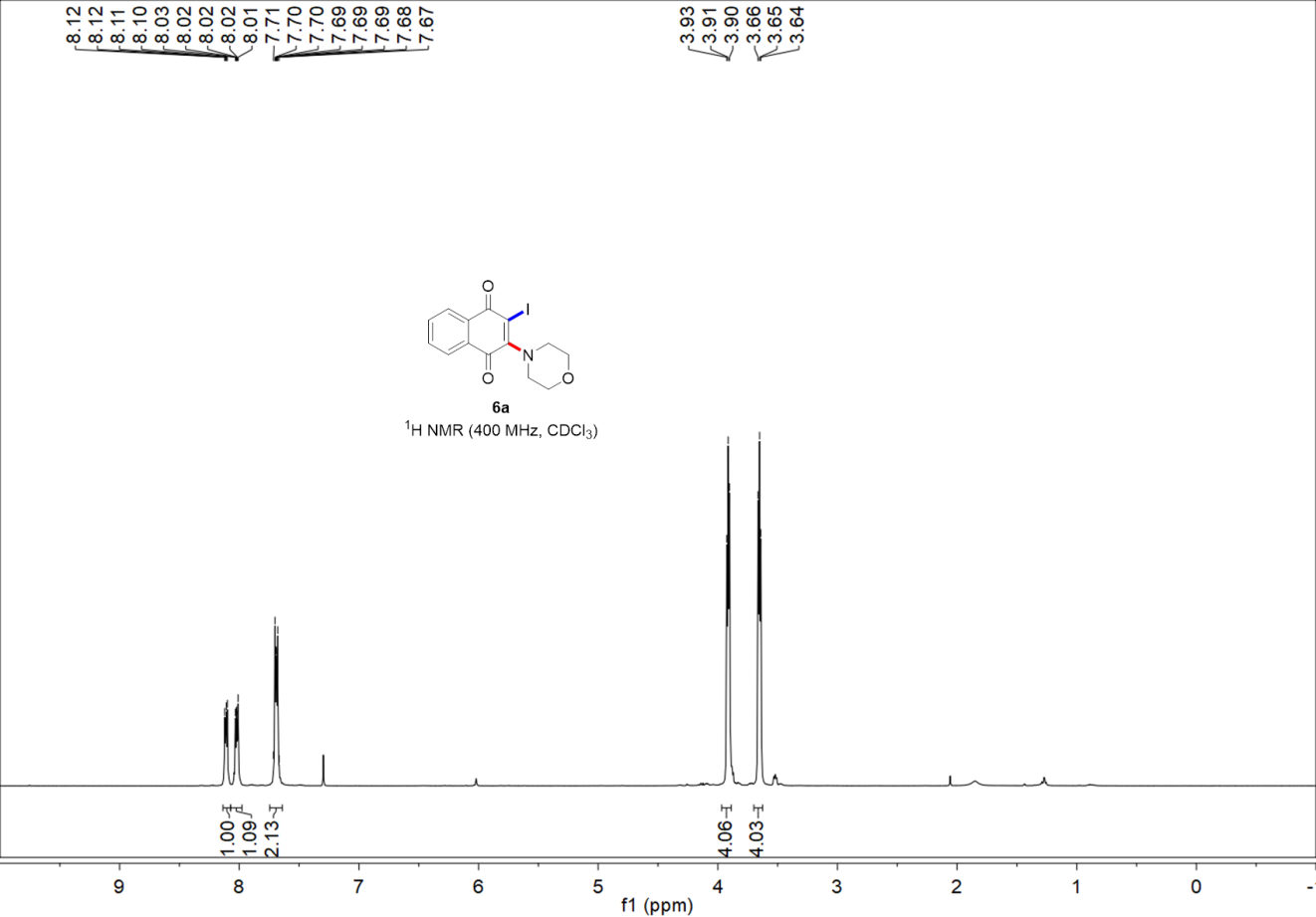
**

**
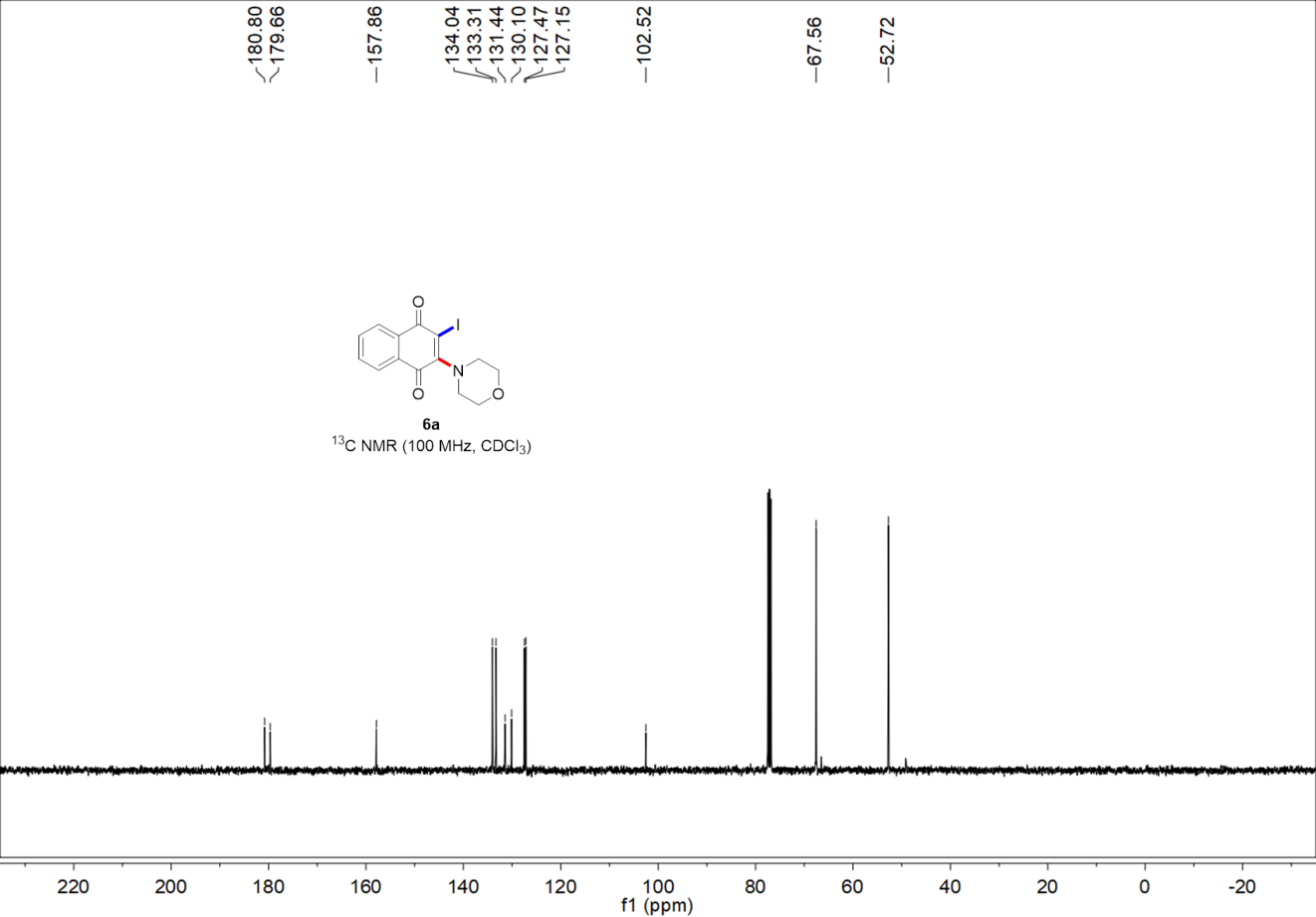
**

**
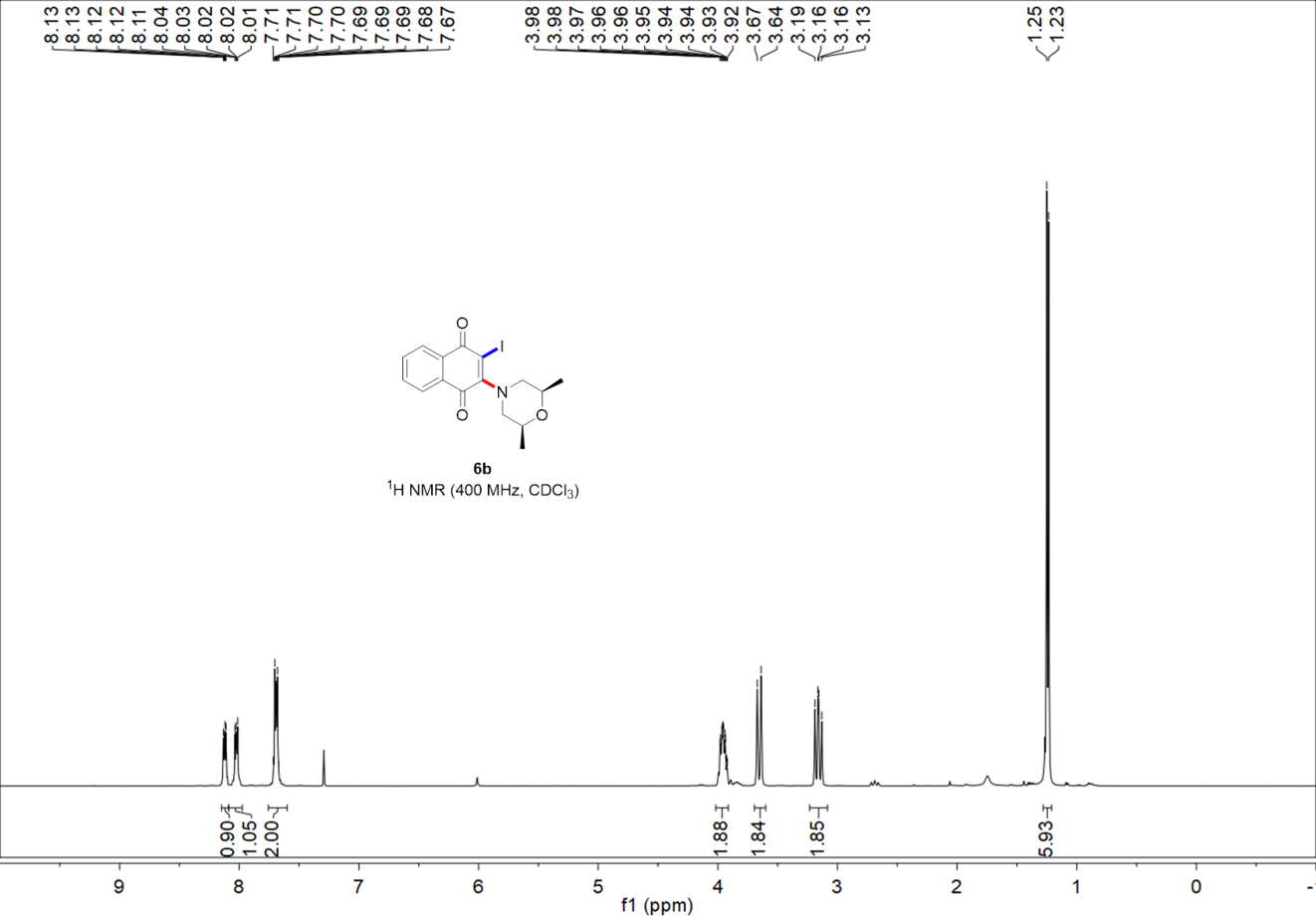
**

**
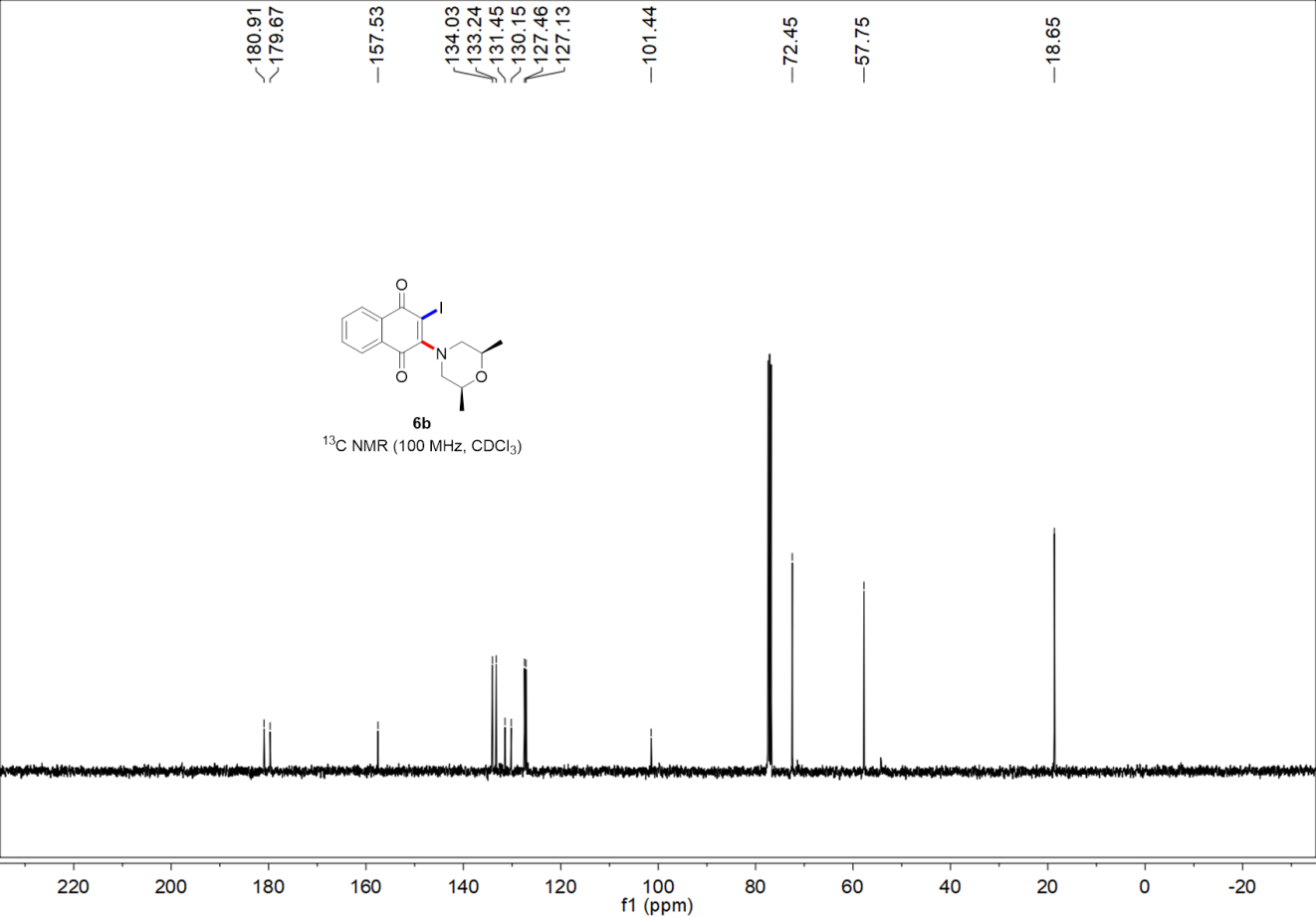
**

**
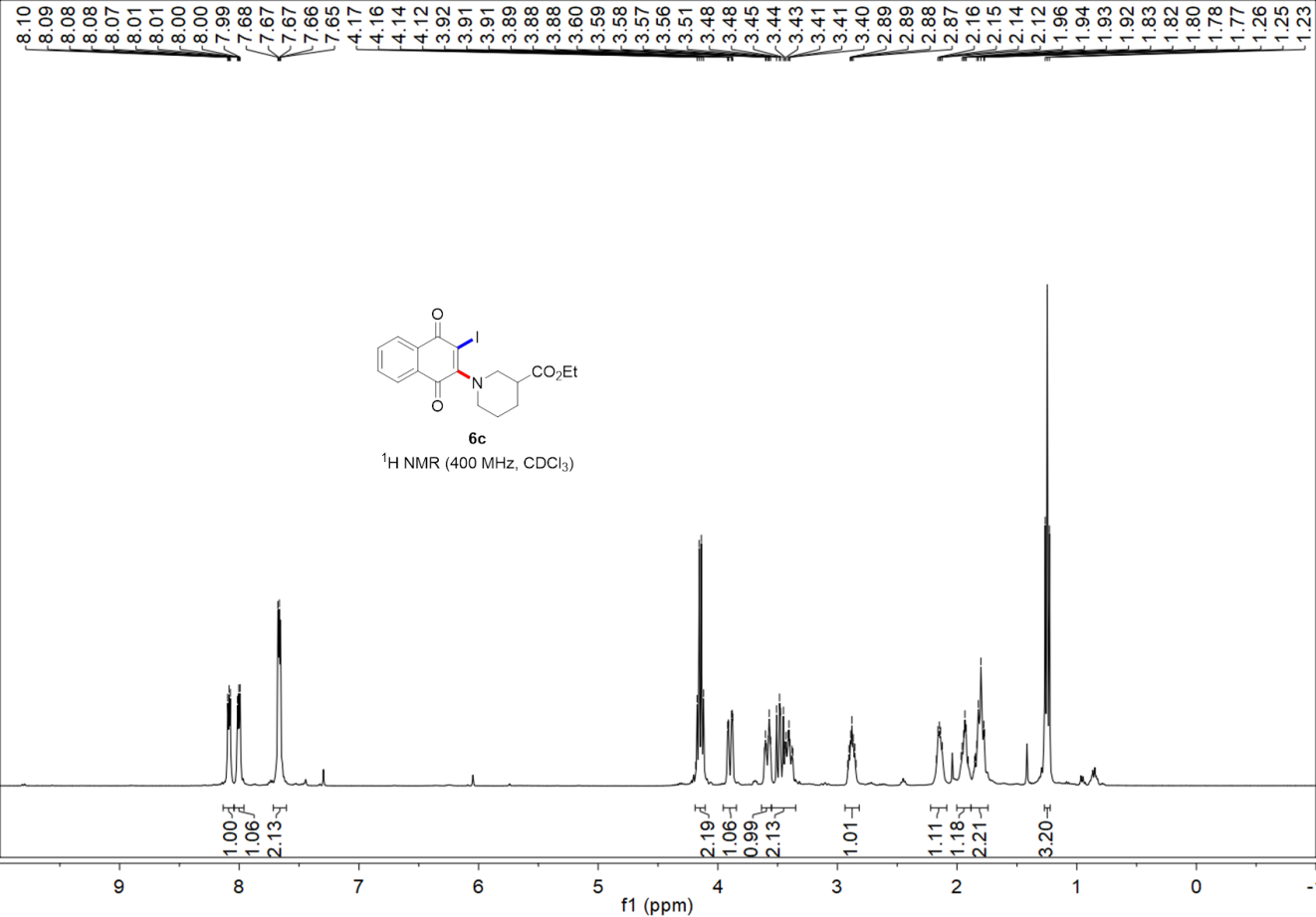
**

**
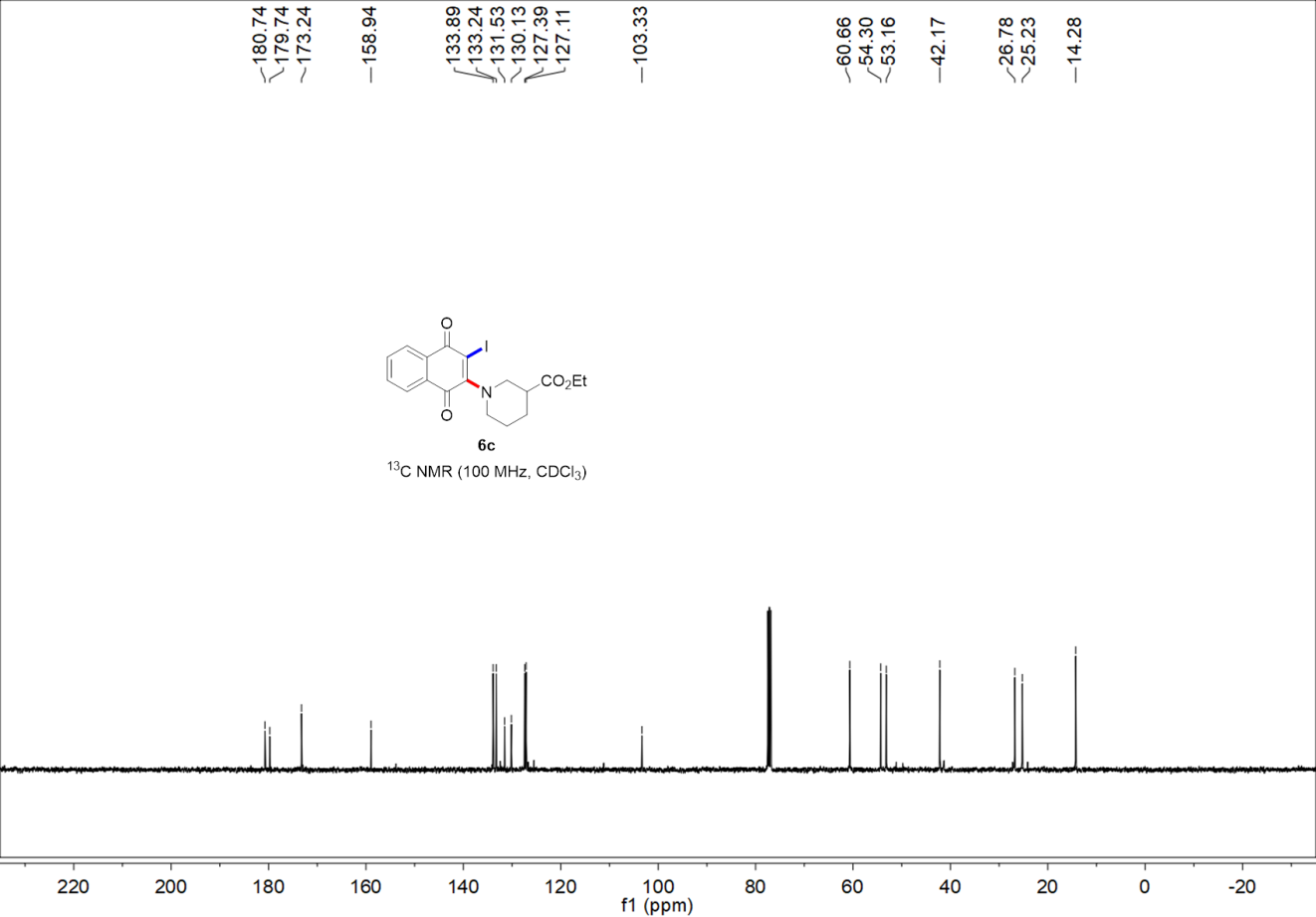
**

**
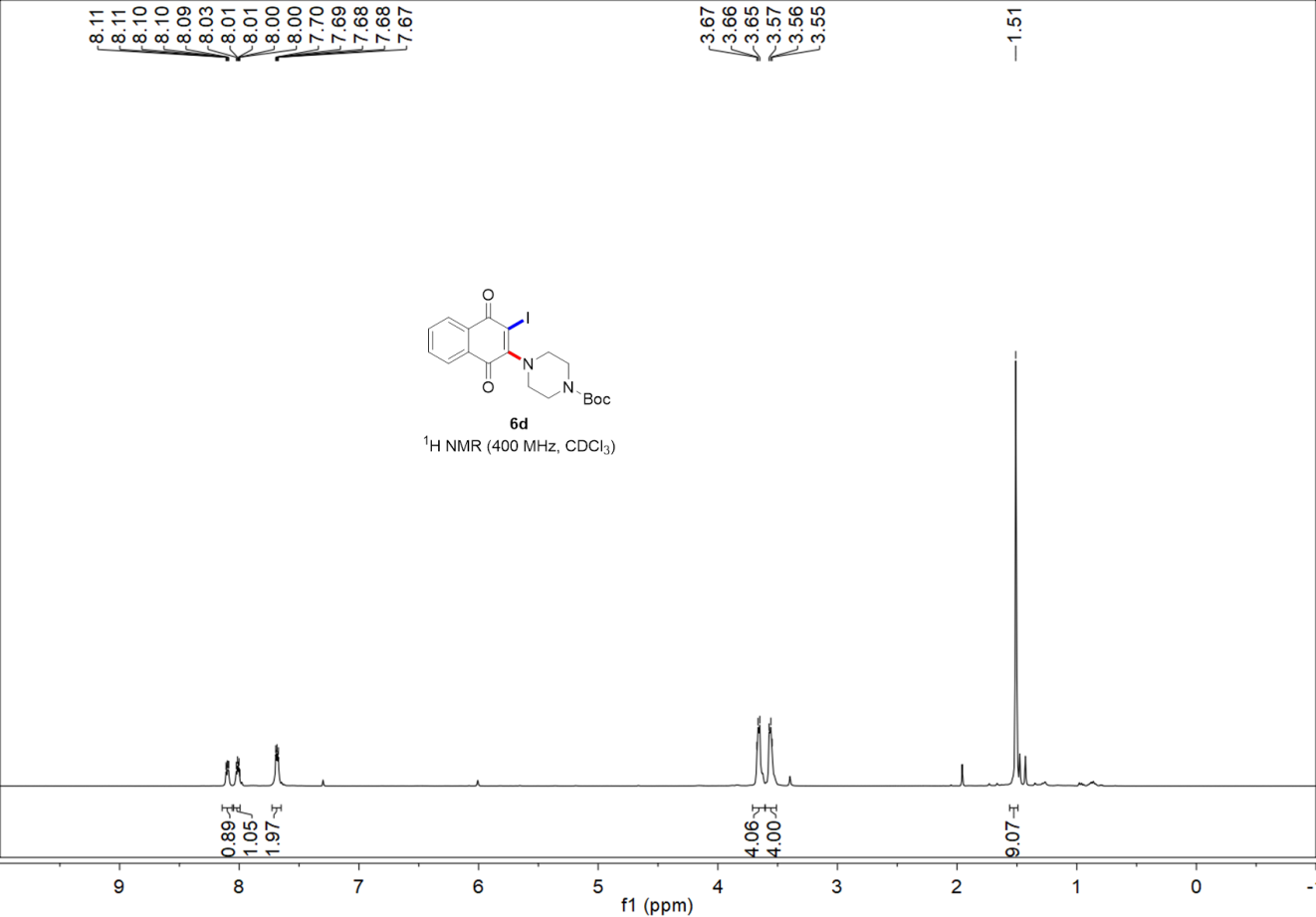
**

**
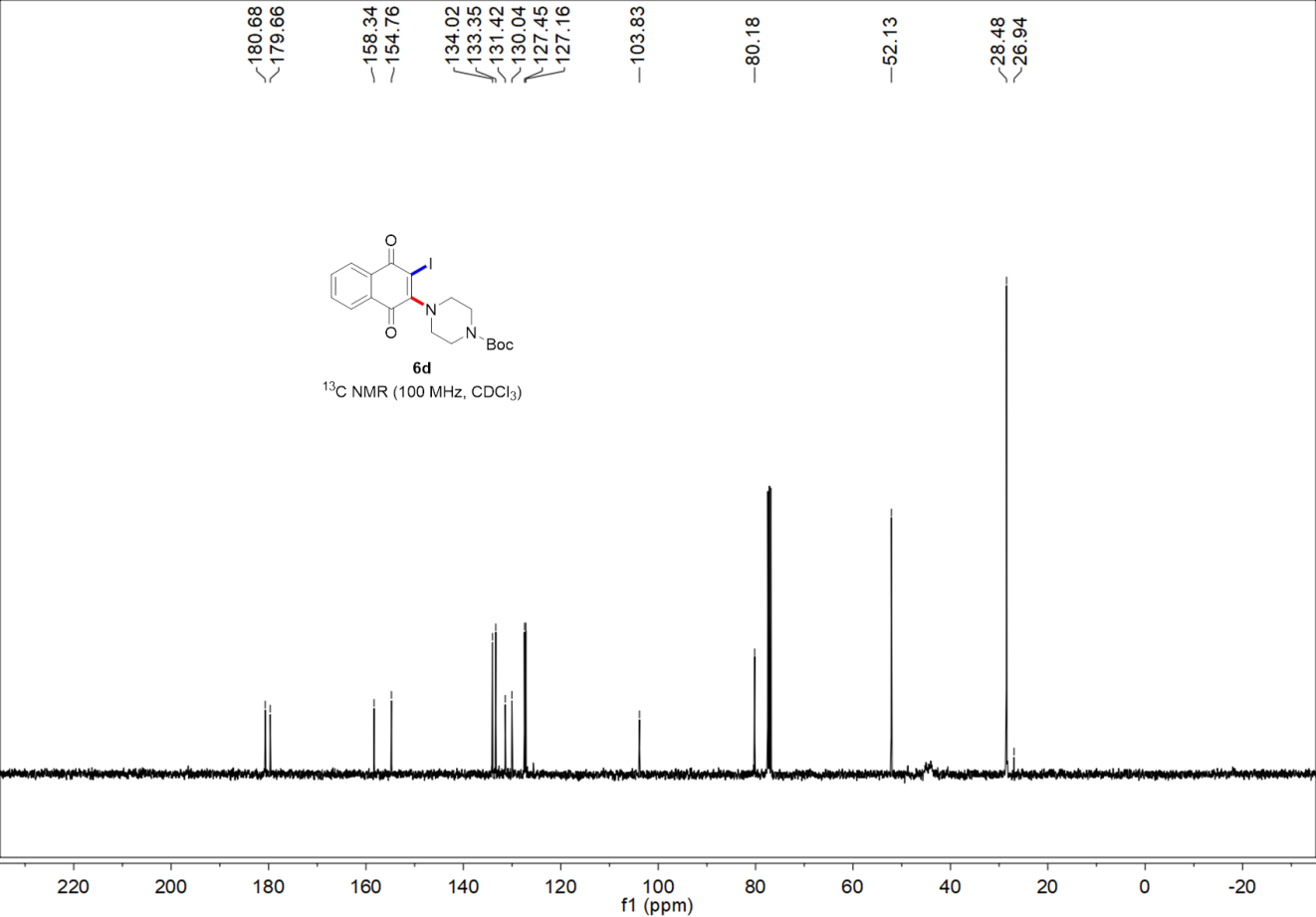
**

**
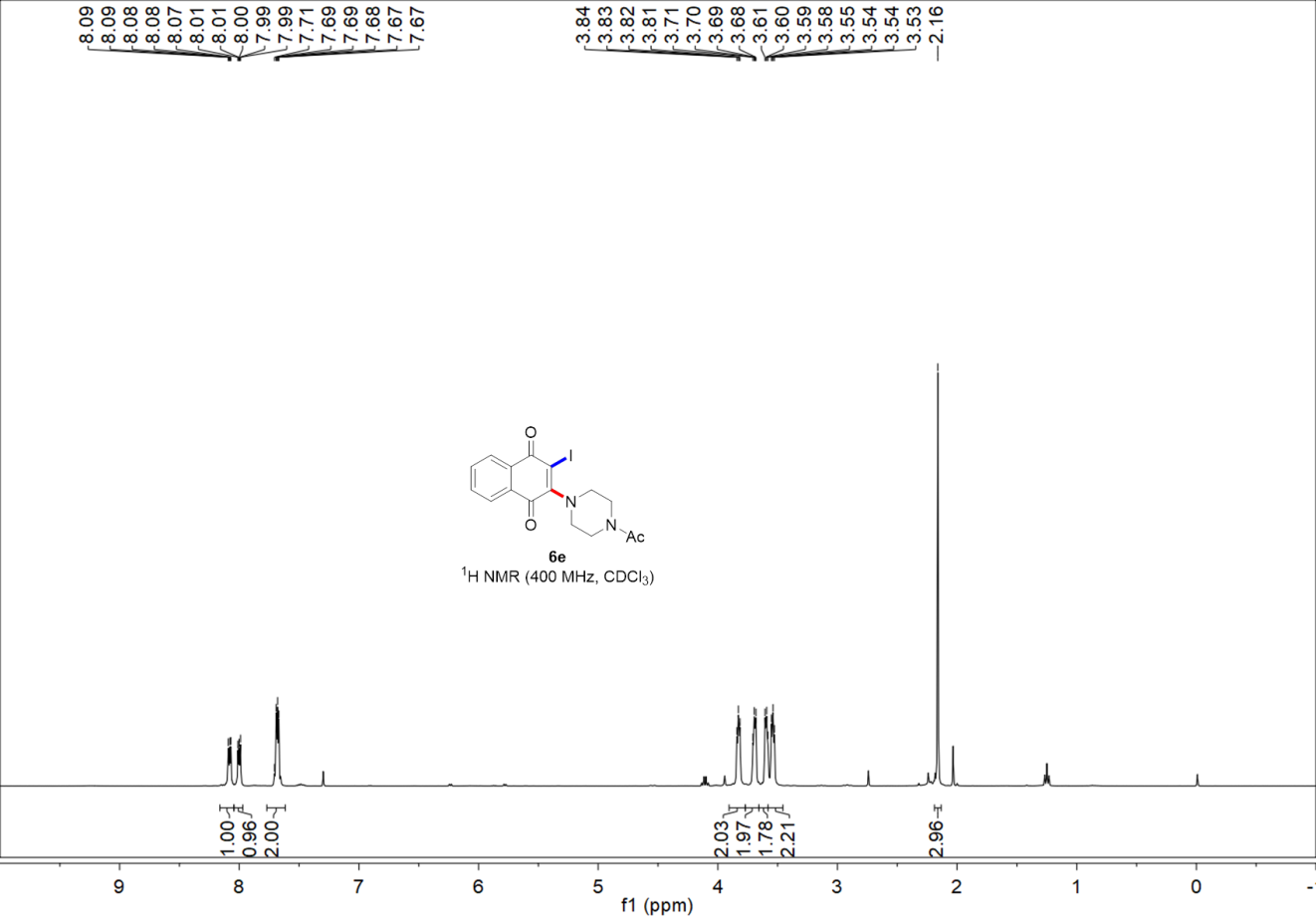
**

**
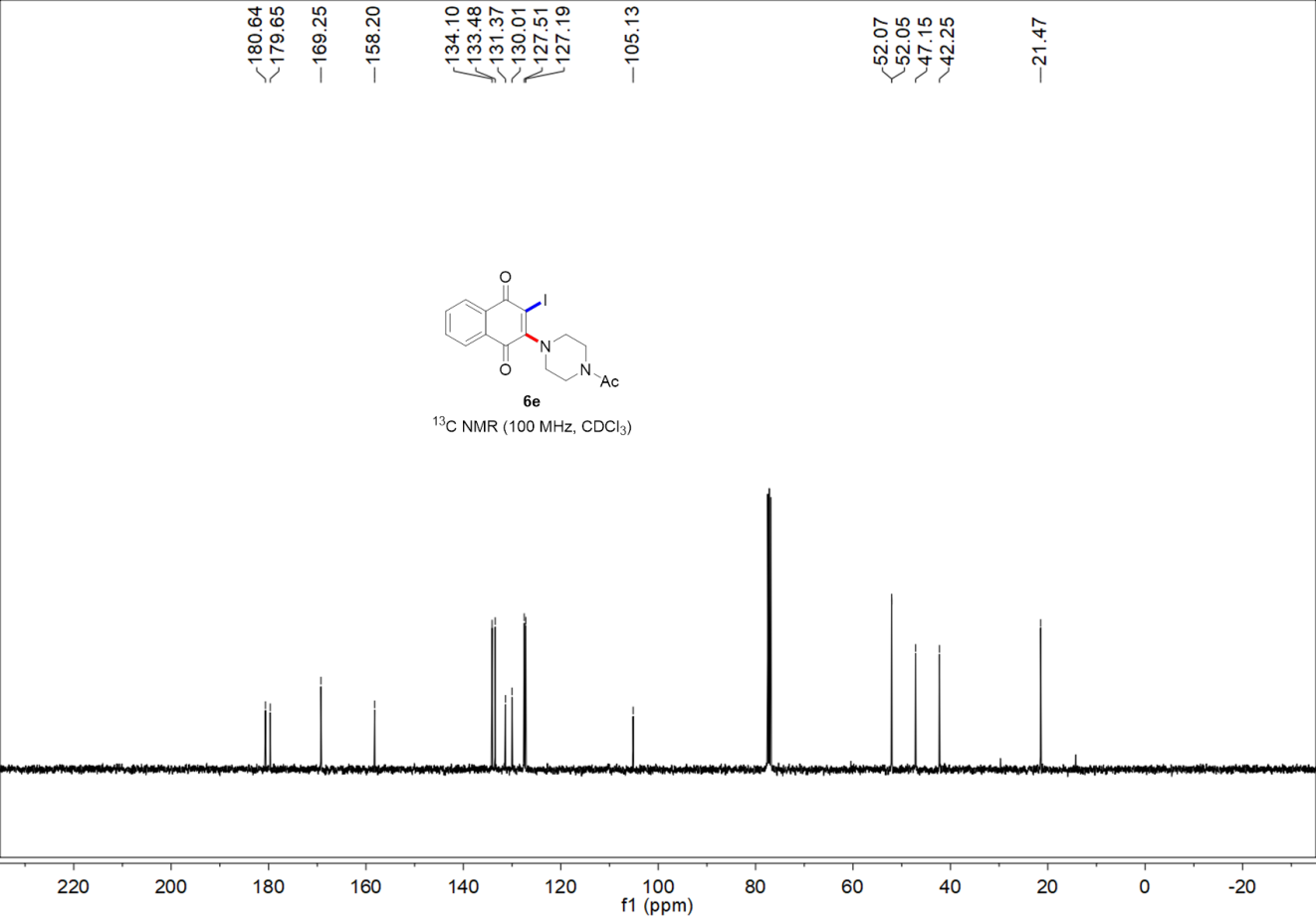
**

**
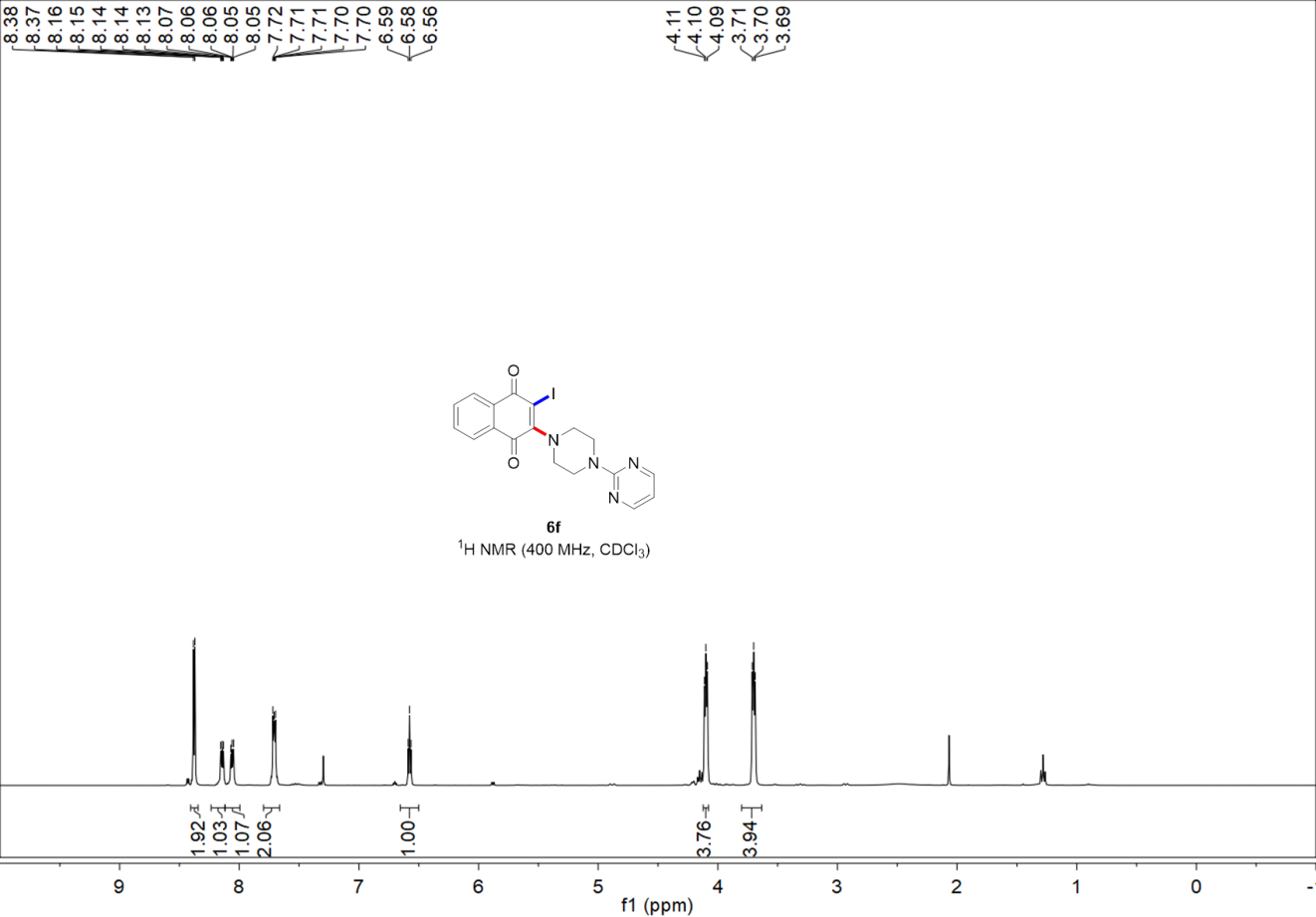
**

**
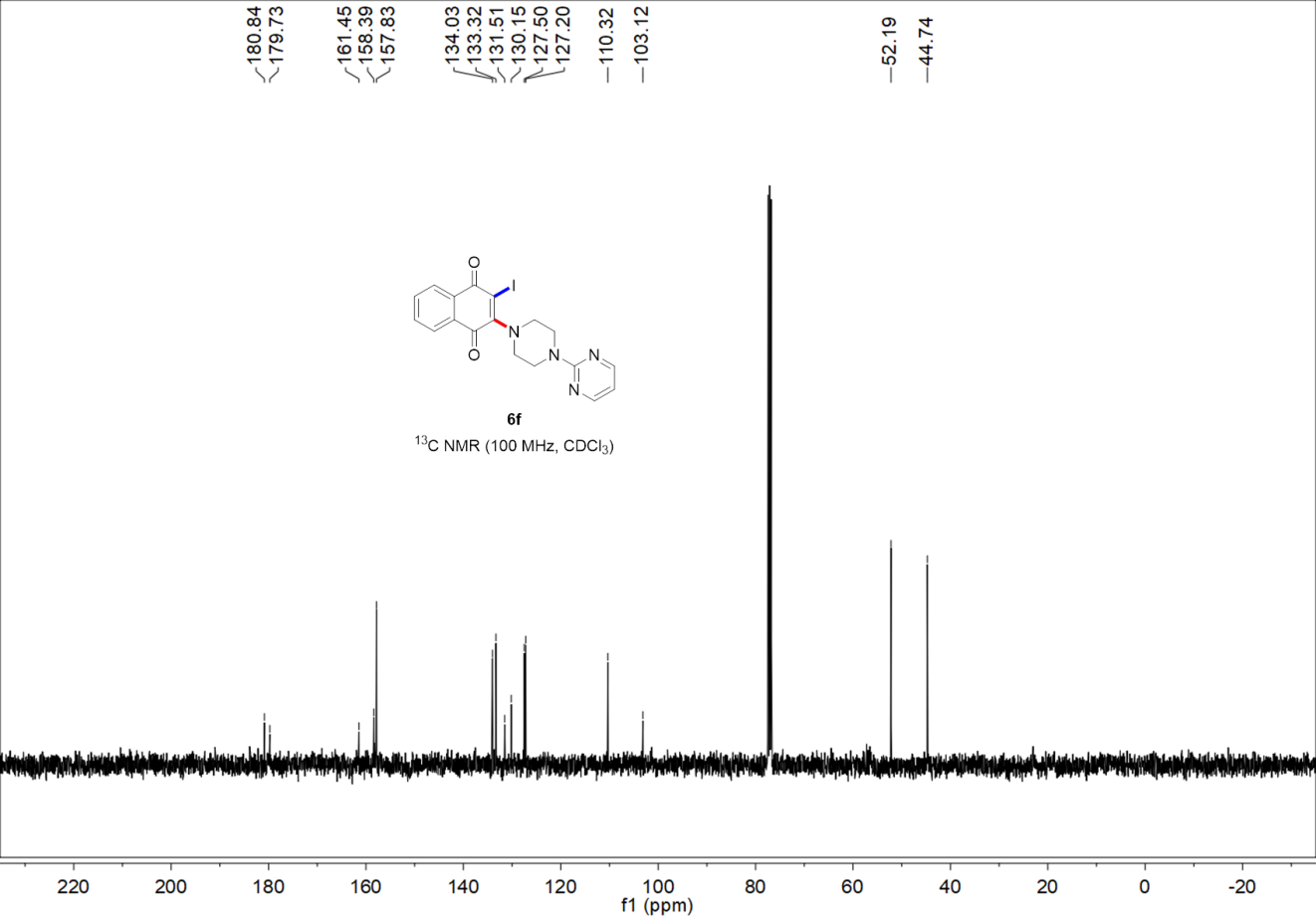
**

**
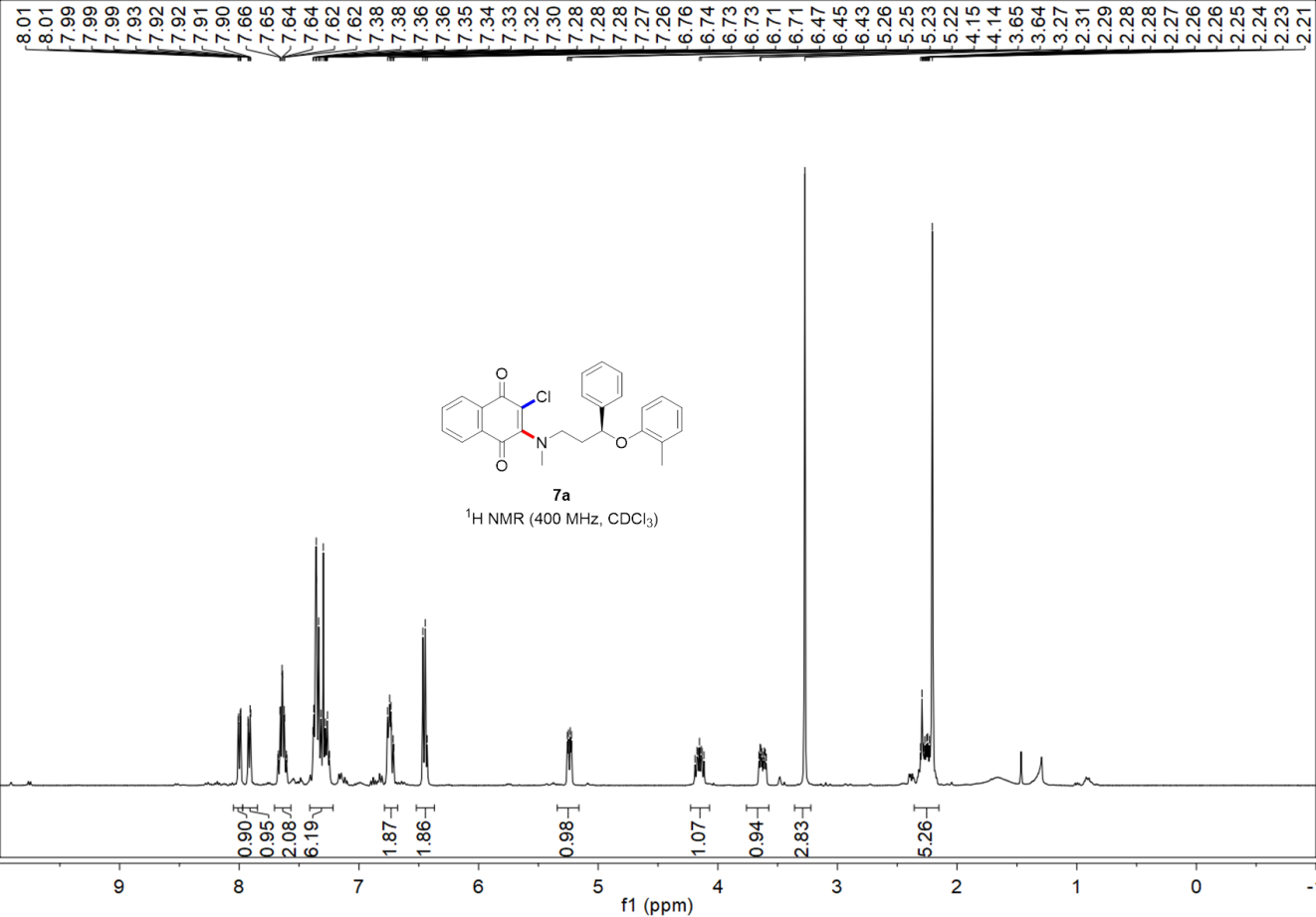
**

**
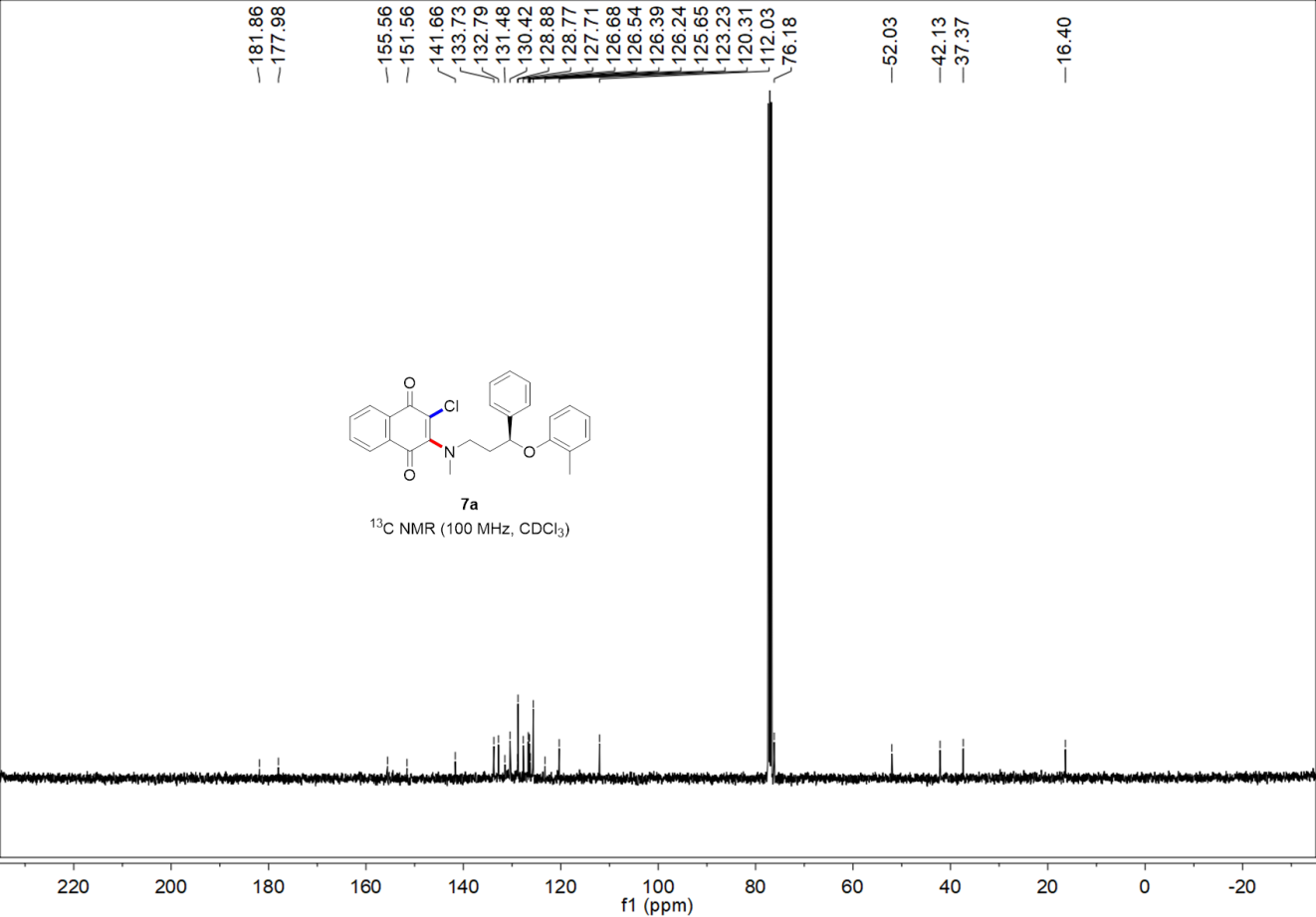
**

**
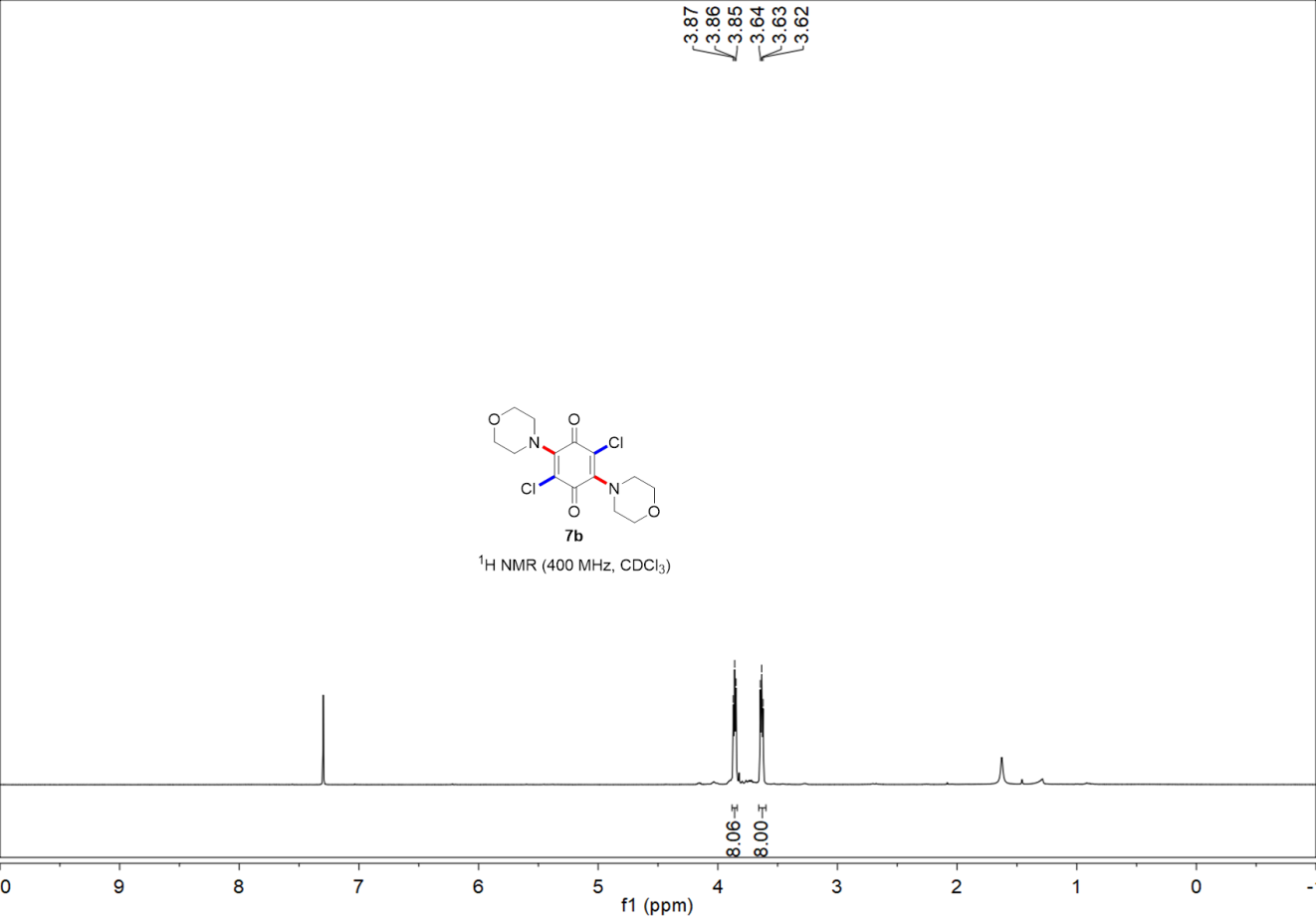
**

**
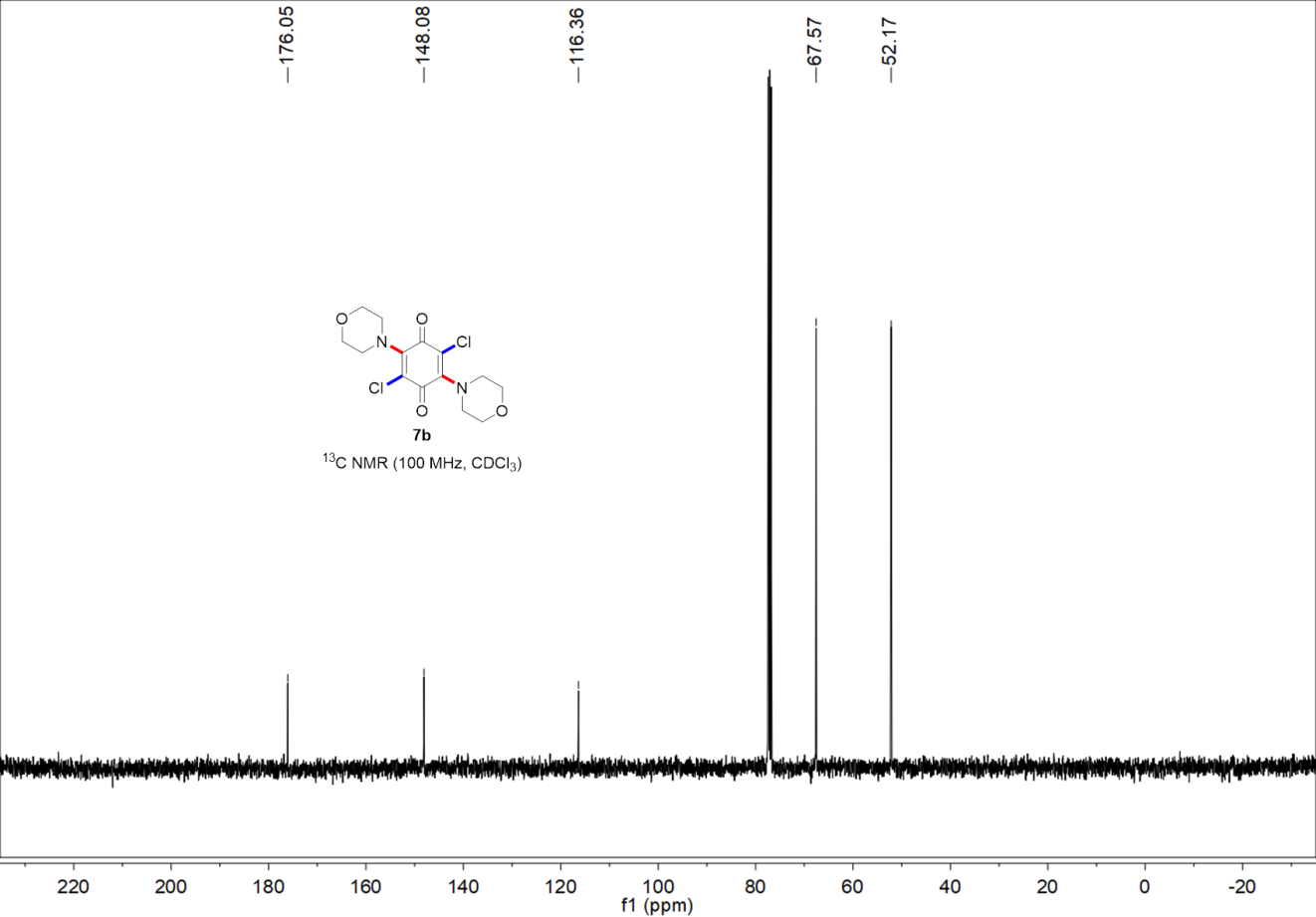
**
